# Human *Survival Motor Neuron* genes generate a vast repertoire of circular RNAs

**DOI:** 10.1093/nar/gkz034

**Published:** 2019-01-30

**Authors:** Eric W Ottesen, Diou Luo, Joonbae Seo, Natalia N Singh, Ravindra N Singh

**Affiliations:** Iowa State University, Biomedical Sciences, Ames, IA 50011, USA

## Abstract

Circular RNAs (circRNAs) perform diverse functions, including the regulation of transcription, translation, peptide synthesis, macromolecular sequestration and trafficking. Inverted Alu repeats capable of forming RNA:RNA duplexes that bring splice sites together for backsplicing are known to facilitate circRNA generation. However, higher limits of circRNAs produced by a single Alu-rich gene are currently not predictable due to limitations of amplification and analyses. Here, using a tailored approach, we report a surprising diversity of exon-containing circRNAs generated by the Alu-rich *Survival Motor Neuron* (*SMN*) genes that code for SMN, an essential multifunctional protein in humans. We show that expression of the vast repertoire of *SMN* circRNAs is universal. Several of the identified circRNAs harbor novel exons derived from both intronic and intergenic sequences. A comparison with mouse *Smn* circRNAs underscored a clear impact of primate-specific Alu elements on shaping the overall repertoire of human *SMN* circRNAs. We show the role of DHX9, an RNA helicase, in splicing regulation of several *SMN* exons that are preferentially incorporated into circRNAs. Our results suggest self- and cross-regulation of biogenesis of various *SMN* circRNAs. These findings bring a novel perspective towards a better understanding of *SMN* gene function.

## INTRODUCTION

Circular RNAs (circRNAs) are a widely expressed class of non-colinear RNAs (NCRs) generated in a diverse set of eukaryotic organisms, including animals, plants, yeasts and protists (1–3). Due to their lack of 5′ and 3′ termini, circRNAs are extremely stable. They are suggested to carry out diverse functions including sponging of microRNAs (miRNAs), sequestration and trafficking of proteins, regulation of transcription and generation of short proteins (4–6). The most common method of circRNA biogenesis is through backsplicing, in which the 5′ splice site (5′ss) of a downstream exon is paired with the 3′ss of an upstream exon (1). Backsplicing can occur in both linear pre-mRNAs and within lariat intermediates harboring skipped exons (7,8). In the absence of backsplicing, lariat intermediates can directly produce circRNAs by degradation of the linear 3′ sequence of the lariat (9). The latter mechanism is predominantly used for producing intron-only-containing circRNAs. There is also evidence to suggest that tissue-specific factors independently regulate biogenesis of circRNAs (10–12). While recent transcriptome-wide analyses of linear RNA-depleted samples reveal a surprising diversity and abundance of circRNAs produced by the human genome (13,14), findings also underscore several limitations of the currently available algorithms in accurately mapping unusual and novel splice site junctions used for the generation of circRNAs (15). Therefore, the current number of circRNAs known to be generated by the human genome in all probability represents an underestimate.

Backsplicing, similar to forward splicing, is likely regulated by a complex combinatorial control in which both cis-elements and transacting factors play important roles. Based on the usage of the same 3′ss for the generation of both linear and circRNAs in most cases, it has been suggested that circRNAs are produced in competition with linear RNAs (16). One of the defining features of backsplicing events appears to be the role of an RNA secondary structure formed by inverted short repeats within intronic sequences upstream and downstream of the 3′ and 5′ss. Among the most common sources of such inverted repeats are short interspersed nuclear elements (SINEs) including primate-specific Alu elements that make up a large portion (∼11%) of the human genome (17–19). Alu elements are ∼300-bp bipartite motifs that have dramatically impacted the evolution of the human genome through their consequential effects on chromatin structure, transcription, DNA repair and pre-mRNA splicing (20–28). Inverted Alu repeats (IARs) promote circRNA generation due to their ability to loop-out sequences by forming stable double-stranded RNA structures (19,29–31). Consistently, depletion of DHX9, an RNA helicase that specifically disrupts the IAR-associated-double-stranded RNA structures, was shown to enhance the generation of a subset of circRNAs (32). Alu elements themselves harbor sequences resembling splice sites and account for ∼5% of alternatively spliced exons in humans (33–35). However, this number could be even larger since linear transcripts carrying Alu exons harboring premature termination codon escape detection due to nonsense-mediated decay (27). Currently, it is not known if Alu-derived exons have a higher representation in circRNAs than in linear transcripts. In addition to promoting circRNA generation, IARs facilitate production of trans-spliced RNAs (tsRNAs) in which exons from two different transcripts of the same or different genes are ligated together (36). There is also evidence to suggest that IARs facilitate generation of trans-spliced circRNAs (ts-circRNAs), albeit with lower frequency (36). As per current estimate, circRNA levels within human cells vary from 2 to 4% of total mRNA and may even be higher in certain cell types (15). However, there is no systematic study capturing the higher limits of diversity of circRNAs and ts-circRNAs generated by a single gene. According to the increasingly accepted transcription-coupled model of splicing, intron removal occurs during transcription and the rate of transcription elongation by RNA polymerase II (pol II) affects the outcome of splicing (37). It has been also suggested that the secondary structure of pre-mRNA affects the rate of pol II elongation and may even cause premature termination of transcription (38). Hence, it is expected that secondary structures associated with IARs regulate circRNA generation through modulation of transcription elongation and/or transcription termination.

Humans carry two nearly identical copies of the *Survival Motor Neuron* genes: *SMN1* and *SMN2* (39). Both *SMN* genes generally contain nine annotated exons (exons 1, 2A, 2B, 3, 4, 5, 6, 7 and 8), with exon 7 being the last coding exon. Hence, exon 8, the final annotated exon, primarily serves as the 3′ untranslated region. Rarely, a portion of intron 6 derived from the left arm of an antisense AluY element is exonized, creating an additional exon called 6B (40). *SMN1* codes for SMN, a multifunctional protein associated with RNA metabolism including small nuclear ribonucleoprotein (snRNP) biogenesis, transcription, translation and RNA trafficking (41). *SMN2* primarily codes for a truncated protein, SMNΔ7, due to predominant skipping of *SMN2* exon 7 (42–44). Low levels of SMN caused by deletion or mutation of *SMN1* leads to spinal muscular atrophy (SMA), one of the leading genetic diseases associated with infant mortality (45). Considering *SMN2* is universally present in SMA patients, correction of *SMN2* exon 7 splicing is considered to be an attractive approach for the treatment of SMA (46,47). Numerous *cis*-elements and transacting factors have been implicated in regulation of *SMN2* exon 7 splicing (48). Our discovery of intronic splicing silencer N1 (ISS-N1) led to the development of an antisense oligonucleotide (ASO)-based drug, Spinraza™ (nusinersen), currently approved for the treatment of SMA (49–51). Engineered U1 snRNPs (eU1s) annealing to ISS-N1 or in its vicinity also promote *SMN2* exon 7 inclusion and could potentially serve as a therapeutic alternative for the treatment of the disease (52,53). ISS-N1 is a complex regulatory element that harbors a cryptic 5′ss (Cr1) overlapping negative cis-elements as well as an inhibitory RNA structure (51,53). Activation of Cr1 by an eU1 extends the length of exon 7 by 23 nucleotides (nt) in *SMN* carrying pathogenic mutations at the 3′ or 5′ss of exon 7 (53). In addition to Cr1, several other cryptic splice sites have been reported within *SMN* genes (53). However, it is not known if any of these sites are also used to generate circRNAs.

**Figure 1.**
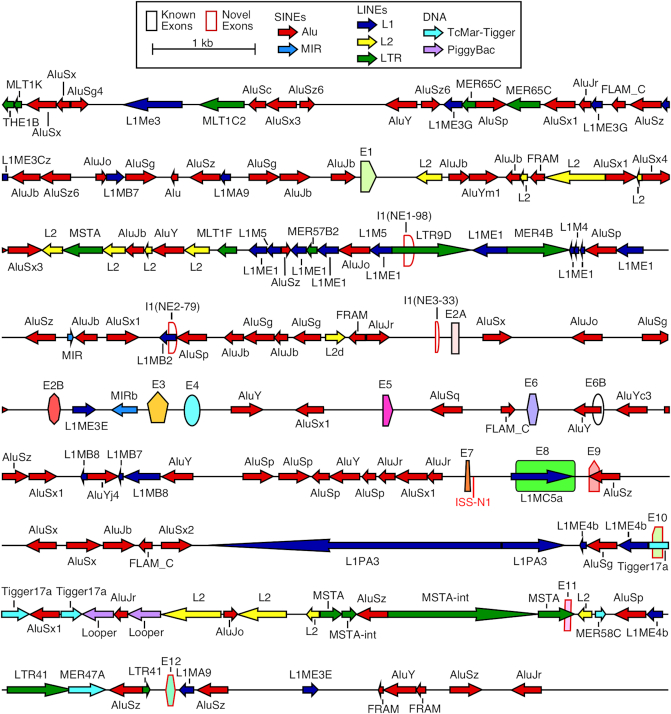
*SMN* genes are rich in Alu repeats. A scale depiction of the *SMN1* gene and surrounding intergenic sequences is shown. Exons are depicted by colored shapes. Previously identified exons are outlined in black, novel exons identified in this study are outlined in red. Repeat sequences as identified by Repeatmasker are depicted by colored arrows. Arrow direction indicates the orientation of the repeat sequence.

The *SMN* genes are highly enriched in SINEs, especially Alu elements that are likely to provide a multilayered control of *SMN* gene expression (Figure 1; 54). More than forty Alu elements occupy ∼39% of the transcribed region of *SMN*. Sequences located upstream and downstream of the *SMN* transcribed region are also rich in Alu elements (Figure 1). Despite an exceptional abundance of Alu elements within *SMN*, exon 6B is the only Alu-derived *SMN* exon that has been reported thus far (40), supporting a notion that there is strong evolutionary pressure against exonization of intronic Alu elements (27). As of now, only two *SMN* circRNAs, one containing exons 2B-to-4 and the other containing exons 5 and 6, have been reported in circBase repository but not yet experimentally validated (54,55). In addition, no trans-splicing event involving endogenous *SMN* sequences has yet been described. We attribute the low number of the currently reported NCRs (circRNAs, tsRNAs and/or ts-circRNAs) of *SMN* to the known technical challenges associated with the amplification of the less abundant NCRs combined with the serious limitations of the existing mapping algorithms that fail to accurately predict NCRs (15). However, given their unusually high SINE content, we reason that the human *SMN* genes present an ideal system to test the higher limits of diversity of NCRs generated from a single gene.

Here we employ a systematic approach to identify and characterize *SMN* circRNAs expressed in various human cell lines and tissues. Our results reveal near universal expression of an immense repertoire of *SMN* circRNAs. Analysis of more than five hundred sequences that we cloned confirmed that nearly all 5′ss of *SMN* participated in backsplicing events. We show usage of several novel splice sites as well as incorporation of three novel exons derived from intronic sequences and four novel exons derived from the intergenic sequences downstream of the final annotated exon of *SMN*. We confirm incorporation of novel intergenic exons in both linear and circular transcripts of *SMN*. Our results also reveal unexpected trans-splicing events leading to the insertion of foreign exonic sequences into several *SMN* ts-circRNAs. A comparison of circRNAs generated by the mouse and human genes suggest a clear impact of Alu elements on shaping the overall repertoire of human *SMN* circRNAs. Depletion of DHX9 supports the role of the IAR-associated-double-stranded RNA structures in the generation of a subset of *SMN* circRNAs and implies competing mechanisms by which specific *SMN* exons are incorporated into linear vs. circular transcripts. Our results suggest self- and cross-regulation of various circRNAs of *SMN*. Our findings substantially expand the size of the *SMN* gene and open the door for exploring additional regulatory mechanisms by which transcription and splicing of *SMN* are governed. Findings also bring a novel perspective towards a better understanding of *SMN* gene function.

## MATERIALS AND METHODS

### Cell culture

All tissue culture media and reagents were purchased from Life Technologies. HeLa, HEK-293 and SH-SY5Y cells were obtained from American Type Culture Collection. Primary fibroblast cells from an SMA type I patient (GM03813) were obtained from Coriell Cell Repositories. Mouse NSC-34 cells were obtained from Dr. Neil Cashman (University of Toronto). HeLa, HEK-293 and mouse NSC-34 cells were cultured in Dulbecco's modified Eagle's medium (DMEM) supplemented with 10% fetal bovine serum (FBS). SH-SY5Y cells were cultured in a 1:1 mixture of minimum essential medium (MEM) and F-12 nutrient mixture supplemented with 10% FBS. GM03813 cells were cultured in MEM supplemented with 15% FBS and 2 mM GlutaMAX.

### RNA isolation and RNase R treatment

Total RNA was isolated from cultured cells using TRIzol reagent (Life Technologies) following the manufacturer's instructions. RNA concentration was determined using a Biomate 3 spectrophotometer (Thermo Scientific). Total RNA was then treated with RQ1 RNase-free DNase (Promega) according to the manufacturer's instructions followed by phenol:chloroform extraction, ethanol precipitation and re-measurement of RNA concentration. Total RNA from human tissues was purchased from TaKaRa Bio. RNase R treatment was performed on 2 μg of total RNA per 10 μl reaction using 0.5 μl (10 U) of RNase R (abm) for 45 min at 37°C. Mock treatments in which the enzyme was omitted were carried out in parallel in order to assess the efficiency of digestion.

### Reverse transcription and PCR (RT-PCR)

cDNA was generated from RNase R-treated or mock-treated RNA using Superscript III reverse transcriptase (RT) (Invitrogen) following the manufacturer's instructions. 0.5 μg RNA was used as template per 5 μl RT reaction. To prime the reactions, 0.5 pmol of exon-specific primers were used (Supplementary Table 1). PCR was performed using Taq DNA polymerase (New England Biolabs, NEB) following the manufacturer's instructions. For circRNA amplification, primers followed a divergent amplification design, in which the 5′ primer was placed at the 3′ end of the exon of interest and the 3′ primer was placed at the 5′ end. For linear RNA amplification, primers followed a standard convergent design. Primer sequences are listed in Supplementary Table S1. PCR products were separated on native polyacrylamide gels and visualized by ethidium bromide staining. To assess splicing of linear *SMN*, we employed a multi-exon-skipping detection assay (MESDA) (56). For radioactive MESDA, the 3′ primer was labeled with [γ-^32^P]ATP (Perkin-Elmer) using T4 polynucleotide kinase (NEB) and desalted using a Micro BioSpin P-30 spin column (Bio-Rad). After completion of electrophoresis, radioactive gels were dried, exposed to phosphorimager screens, and scanned using a FujiFilm FLA-5100 system.

### Quantitative Real-time RT-PCR (qPCR)

0.6 μg of DNase-treated total RNA was reverse transcribed in a 5 μl reaction using SuperScript III RT. The cDNA was further diluted 20-fold with RNase-free water, and 3 μl was then used in a 20 μl of PCR reaction using FastStart Universal SYBR Green Master mix (ROX) (Roche). qPCR was performed using a Stratagene Mx3005 qPCR system (Agilent Technologies). Primer sequences are listed in Supplementary Table S1. Data was analyzed using ΔΔCt method with control set as the reference condition. For DHX9, DDX5 or DDX17 depleted samples, cDNA was generated using oligo(dT)_12–18_ (Invitrogen), and the housekeeping gene, GAPDH, was used for normalization. For DRB (5,6-dichlorobenzimidazole 1-β-D-ribofuranoside) treated samples, cDNA was generated using random primers (Promega), and 18S rRNA was used for normalization.

### Identification of circRNAs

Bands of interest corresponding to RT-PCR products were excised from a 6% native polyacrylamide gel, and DNA was recovered by using ‘crush and soak’ method (56). A pGEM-T Easy vector (Promega) was utilized to clone the recovered DNA following the manufacturer's instructions. After initial screening by blue/white colony selection on indicator plates, colonies were subjected to a secondary screen using colony PCR. Colonies with plasmids that carried inserts of the expected size were propagated and plasmids were purified using the QIAprep Spin Miniprep Kit (Qiagen). Insert identities were determined by Sanger sequencing. All novel linear and circular RNAs sequences of *SMN* and *Smn* can be accessed through the GenBank database at NCBI and are also listed in Supplementary Tables S2 and S3, respectively.

### Polyadenylated mRNA enrichment

After isolation of total RNA, intact poly(A)^+^ RNA was isolated using a Magnetic mRNA Isolation kit (NEB) following the manufacturer's instructions. 40 μg total RNA was used as input, and after enrichment, poly(A)^+^ RNA was eluted in 100 μl of elution buffer. RNA was then concentrated by ethanol precipitation and resuspended in 10 μl of nuclease-free water. An estimated 0.1 μg poly(A)^+^ RNA (2.5 μl) was used as template per 5 μl RT reaction.

### siRNA knockdown

To knock down the protein of interest, 1 × 10^6^ HeLa cells were reverse transfected in six-well plates with 50 nM ON-TARGETplus siRNA (Dharmacon) or a non-targeting pool of siRNA (Dharmacon) using Lipofectamine 2000 (Life Technologies) following the manufacturer's instructions. Target sequences of siRNAs are listed in Supplementary Table S4. Six hours after transfection, the culture medium was replaced with fresh medium. Twenty-four hours after transfection, the cells were trypsinized and transferred to 60 mm dishes in order to prevent overgrowth. At 48 h post transfection, the entire process was repeated for a total of 96 h total transfection time. To collect samples, cells were trypsinized, then transferred to two 1.5 ml microfuge tubes. ∼2/3 of the cells were used for protein extraction and western blotting, ∼1/3 for RNA isolation. Cells were then spun down at 3500 × g for 1 min at 4°C to collect cell pellets. For total RNA isolation, supernatant was removed and cells were lysed immediately in 1 ml of TRIzol reagent and RNA was prepared as described above. For protein extraction, supernatant was removed and cells were resuspended in 1 ml of ice-cold DPBS. The cells were pelleted and the entire procedure was repeated twice for a total of three washes in DPBS. After the final spin, the supernatant was removed and cell pellets were frozen on dry ice and stored at −80°C.

### DRB treatment in cells

DRB was obtained from Sigma. The required amount of DRB was dissolved in DMSO (dimethyl sulfoxide) to prepare 100 mM stock solution. HeLa cells were pre-plated at a density of 0.5 × 10^6^ cells per one well of a six-well plate 24 h prior to treatment. DRB was added with fresh growth medium at a final concentration of 80 μM; control samples were treated with fresh medium alone or with an equivalent amount of DMSO added. Eight hours after treatment initiation, cells were lysed in TRIzol reagent for total RNA isolation and RNase R treatment.

### circRNA knockdown using gapmer ASOs

Modeled on a recent report (57), we designed RNase H-competent gapmer ASOs that anneal to specific splice junctions of circRNAs. All gapmers incorporated phosphorothioate modification throughout the body of the ASO. Terminal 4, 5 or 6 residues on both ends carried 2′-*O*-methyl modified ribonucleotides and the middle 9 or 12 residues carried deoxyribonucleotides. Gapmers were synthesized by Dharmacon, Inc. The sequences of the oligonucleotides used are as follow: CNTRL-9GAP18, 5′-mG*mU*mC*mU*mA*dT*dG*dT*dC*dT*dA*dT*dG*dT*mC*mU*mA*mU-3′; Jn4-2B-9GAP18, 5′-mU*mA*mG*mA*mG*dC*dA*dT*dG*dC*dT*dT*dT*dC*mC*mU*mG*mG-3′; Jn4-2A-9GAP18, 5′-mA*mU*mC*mA*mU*dC*dG*dC*dT*dC*dT*dT*dT*dC*mC*mU*mG*mG-3′; Jn4-3-12GAP23, 5′-mC*mC*mC*mA*mA*mC*dT*dT*dT*dC*dC*dA*dC*dT*dT*dT*dC*dC*mU*mG*mG*mU*mC-3′. In these sequences, the letter ‘m’ represents an *O*-methyl modification at the second position of a sugar residue, the letter ‘d’ represents deoxyribonucleotides, and an asterisk represents a phosphorothioate modification of the backbone. 1 × 10^6^ HeLa cells were reverse transfected in six-well plates with 20 nM gapmer ASOs targeting specific circRNA junctions or a non-targeting control gapmer using Lipofectamine 2000 following the manufacturer's instructions. Six hours after transfection, the culture media was replaced with fresh media. Twenty-four hours after transfection, adherent cells were re-transfected with gapmer at the same concentration. At 24 h post second transfection, cells were collected for RNA isolation using TRIzol reagent for a total of 48 h transfection time.

### Protein extraction and Western blotting

In order to extract proteins from cell pellets, cells were resuspended in 50 μl radioimmunoprecipitation assay (RIPA) buffer (Boston Bioproducts) supplemented with HALT protease inhibitor cocktail (Thermo Scientific). Cells were lysed for 30 min on ice with occasional mixing. Afterwards, lysates were centrifuged at 12 000 × g for 15 min at 4°C and supernatant transferred to fresh pre-chilled 1.5 ml microcentrifuge tubes. Protein concentration was measured by Bio-Rad protein assay (Bio-Rad). An equal volume of 2× SDS loading buffer supplemented with 5% β-mercaptoethanol was added to each sample, followed by boiling for 5 min to denature. Denatured samples were then spun down at maximum speed for 5 min. Then, 20 μg of protein per sample was separated on 10% SDS-PAGE gels and transferred to PVDF membranes using a Transblot Turbo fast transfer system (Bio-Rad). Membranes were blocked in 5% nonfat milk dissolved in Tris-buffered saline containing 0.05% Tween-20 (TBST). Primary antibody incubation was carried out at 4°C overnight. Primary antibody dilutions were as follows: monoclonal mouse anti-DHX9/NDH II 1:80 (Clone E-10, Santa Cruz sc-137183), goat polyclonal anti-DDX5 1:800 (Abcam ab10261), rabbit polyclonal anti-DDX17 1:400 (Santa Cruz sc-86408), rabbit polyclonal anti-β-actin 1:2000 (Sigma A2066), mouse anti-GAPDH 1:4000 (Clone 6C5, Abcam ab8245), mouse anti-α-tubulin 1:3000 (Sigma, T6199). After primary incubation with primary antibodies, membranes were washed thoroughly in TBST and incubated with secondary antibodies conjugated to horseradish peroxidase for 1 h at room temperature. Secondary antibodies and their dilutions were as follows: goat anti-mouse 1:4000 (Jackson 115-035-003), donkey anti-rabbit 1:2000 (GE Healthcare NA934), donkey anti-goat 1:5000 (Santa Cruz sc-2020). After secondary antibody incubations, membranes were washed thoroughly and developed using SuperSignal West Femto Maximum Sensitivity Substrate (Thermo Scientific) or Clarity ECL Blotting Substrate (Bio-Rad). Bands were then visualized using a UVP Biospectrum AC imaging system.

### Computational analysis

To estimate the strength of the 5′ and 3′ss, MaxEntScan scoring algorithm (http://genes.mit.edu/cgi-bin/Xmaxentscan_scoreseq.pl) was used, while HBond score web interface was used to score 5′ss (http://www2.hhu.de/rna/html/hbond_score.php). To locate repetitive elements within genomic sequences, RepeatMasker Web Server (http://www.repeatmasker.org/cgi-bin/WEBRepeatMasker) was adopted. The mfold Web Server (http://unafold.rna.albany.edu/?q=mfold/RNA-Folding-Form) was employed to predict RNA structures.

## RESULTS

### *SMN* generates a diverse set of circRNAs harboring the 5′-terminal exons

In order to identify circRNAs generated by the 5′-terminal exons of *SMN*, we prepared cDNA libraries using RNase R-treated total RNA as template and reverse transcription (RT) primers annealing to the first five internal exons (2A, 2B, 3, 4 and 5). We then performed PCR employing divergent primers annealing to each of these exons. To capture cell-specific differences in the repertoire of *SMN* circRNAs, we conducted these experiments in four cell lines: HeLa, human embryonic kidney-derived (HEK-293), SMA patient fibroblasts (GM03813) and neuronal SH-SY5Y. While HeLa, HEK-293 and SH-SY5Y cells contain both *SMN1* and *SMN2*, GM03813 cells carry only *SMN2* (49,56). We optimized PCR conditions for every primer combination used in this study using total RNA from SH-SY5Y cells and were able to visualize highly expressed circRNAs at 28 cycles of amplification (Supplementary Figure S1B). In contrast, linear *SMN* transcripts were detectable after as few as 22 cycles of amplification, suggesting that each individual circRNA makes up only a very small portion of total RNA expressed from the *SMN* loci (Supplementary Figure S1A). However, due to the sheer number of distinct circRNAs that we observed in this study, we conclude that total expression of circRNAs is non-negligible. Since our goal was to capture the broad spectrum of circRNAs irrespective of their abundance, we performed 35 cycles of PCR amplification. Exon-specific amplifications produced multiple bands for all tested exons. We confirmed the identity of bands by cloning and sequencing. We observed differences in the relative abundance of specific circRNAs when amplified using divergent primers annealing to different exons. This is not unexpected considering the differences in the structural contexts of different exons in a given circRNA. In addition, circRNAs are known to display different levels of RNase R sensitivity that can introduce high variability between replicates even if replicates are generated at the same time (19,58). Therefore, we presume the presence of a circRNA in any one replicate to be affirmative evidence of their formation. However, we consider a circRNA to be a major product only after validation by at least two independent PCR amplifications using divergent primer pairs annealing to two different exons. Unless stated otherwise, all circRNAs reported here were generated employing canonical U2 type (GU/AG) splice sites.

**Figure 2.**
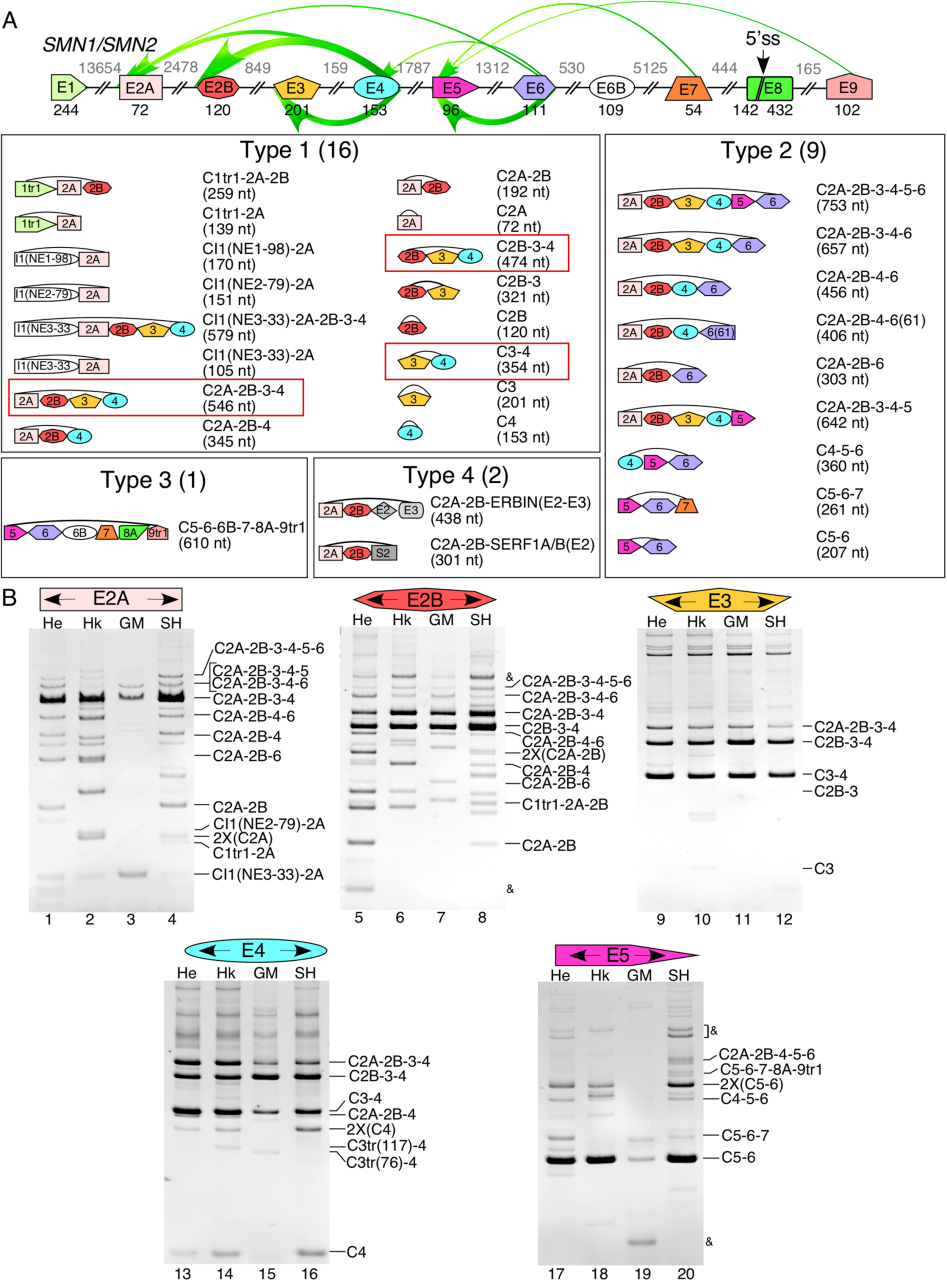
Early exons of *SMN* are involved in formation of a wide variety of circRNAs. (**A**) Top panel: A genomic overview of the *SMN* gene layout is shown. Exons are depicted as colored shapes, and introns are shown as lines/broken lines. Exon sizes are indicated by numbers in black below exons and intron sizes are indicated by numbers in gray above introns. A novel 5′ss in exon 8 is marked with a black arrow. Colored arrows represent backsplicing events, their thickness corresponds to the estimated prevalence of each event. Lower panels: An overview of all identified circRNAs that are produced by canonical backsplicing events. Type 1 circRNAs are produced only by exons 1 through 4; type 2 circRNAs include some early exons and some late exons; type 3 circRNAs include all circRNAs that contain exon 8A and/or downstream sequences. Type 4 circRNAs are produced by trans-splicing events and include non-*SMN* sequences. The most abundant circRNAs are boxed in red. (**B**) Ethidium bromide stained gels of divergent RT-PCR products. Exons to which divergent primers anneal are given at the top of each gel. Cell types used are indicated above each lane. Band identities marked on the right side of each gel were determined by cloning and sequencing. Abbreviations are as follows: He, HeLa; Hk, HEK-293; GM, GM38013; SH, SH-SY5Y. Nomenclature of circRNAs is as follows: ‘C’ followed by all exons in the mature circRNA. ‘I’ indicates exonized intronic sequence. ‘tr’ after the exon number indicates that the exon is 5′ truncated, numbers in parentheses indicate sizes of novel or truncated exons. ‘(2X)’ indicates that the PCR product is produced by rolling circle reverse transcription and PCR amplification across 2 copies of the circRNA. ‘&’ indicates an artefact.

**Figure 3.**
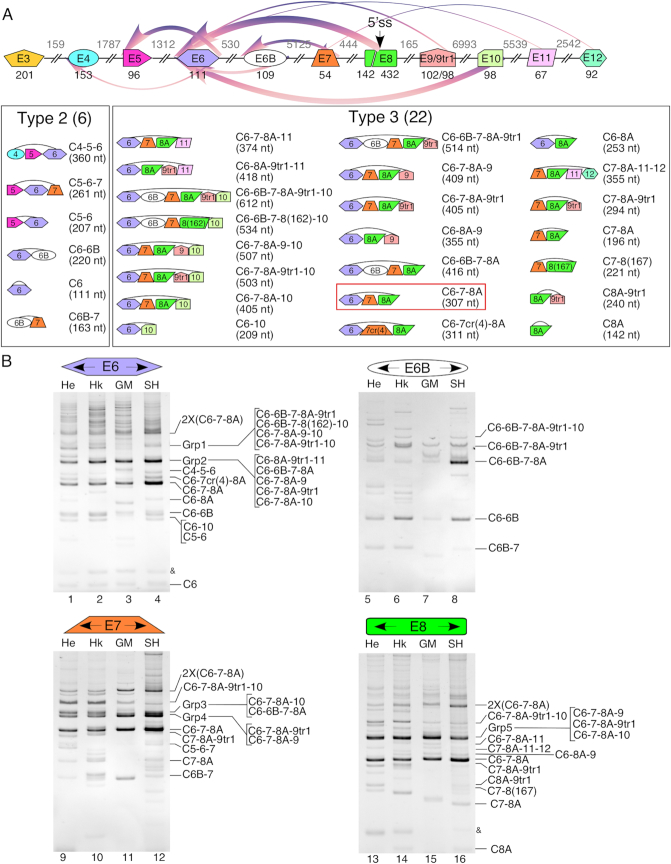
Late exons of *SMN* are involved in formation of a wide variety of circRNAs. (**A**) Top panel: A genomic overview of the 3′ portion of the *SMN* gene layout is shown. Lower panels: An overview of all identified circRNAs that are produced by canonical backsplicing events. Labeling and color coding are the same as in Figure 2. (**B**) Ethidium bromide stained gels of divergent RT-PCR products. Labeling is the same as in Figure 2.

circRNAs exhibit a high degree of diversity, including a wide variety of circRNAs generated from a single gene (13,14). Even so, the sheer number of unique circRNAs generated by *SMN* was surprising. We classified *SMN* circRNAs into four major types based on the nature of the incorporated exons (Figure 2A). Type 1 circRNAs were made using exclusively the early transcribed (5′-terminal) *SMN* region spanning from exon 1 to exon 4. Among the type 1 circRNAs, we identified three novel exons derived from intron 1. We named them I1(NE1-98), I1(NE2-79) and I1(NE3-33) (Figures 1 and 2, Supplementary Figure S2). We considered exons 5, 6, 6B and 7 as middle exons. Type 2 circRNAs were made using at least one middle exon with or without early exons. We considered exon 8 as well as downstream intergenic exons as 3′-terminal exons. Type 3 circRNAs were made using at least one 3′-terminal exon with or without early and middle exons. Exons 9, 10, 11 and 12 are novel exons that are generated from intergenic sequences downstream of exon 8 and are being reported for the first time in this study (Figures 1 and 3, Supplementary Figure S3). All novel exons, including those generated from intron 1 as well as intergenic exons reported in this study use canonical U2 type (GU/AG) splice sites. Type 4 circRNAs of *SMN* harbored at least one exon from a different Alu-rich gene. For the ease of explanation, we adopted a simple nomenclature in which ‘C’ stands for circRNA and numbers in the ascending order represent the exon number of *SMN*. Number following an ‘I’ represents the intron number. The first and the last numbers represent exons and/or introns that provide the 3′ and the 5′ss, respectively. Use of ‘tr’ after the number represents a truncated exon due to usage of a novel 3′ss within that exon. For novel 3′ss that were used in major products, we named the truncated form with a number, e.g. 9tr1. For less commonly used novel 3′ss, we put the new exon size in parentheses after the ‘tr’, e.g. 6tr(61). A capital letter after the number represents usage of a novel 5′ss within that exon, if that 5′ss was used in any major product. For less commonly used novel 5′ss, we added the new exon size in parentheses after the exon number. ‘cr’ after exon name indicates intronic sequence included downstream of 5′ss, while ‘cp’ after exon name indicates intronic sequence included upstream of the 3′ss. For products without canonical splice sites, we adopted a simplified naming system. In these cases, instead of ‘C’, we name each product with ‘T’ followed by the type number, followed by a letter designation. The full exon content and splice site locations of each noncanonical circRNA candidate are given in Supplementary Table S5 and Supplementary Figure S9.

Our results revealed inclusion of early exons into 28 circRNAs, 16 of which belonged to type 1 (Figure 2A, Supplementary Figure S4, Supplementary Table S3). We identified 9, 1 and 2 circRNAs belonging to types 2, 3 and 4, respectively. Out of 28 circRNAs, 12 circRNAs shared the 3′ss of exon 2A in combination with different 5′ss. Detection of C2A, C2B, C3 and C4 in one or more cell lines confirmed single exon circularization for the first four internal exons including the 72-nt long exon 2A, which is one of the shortest exons of *SMN*. However, we did not detect single exon circularization for the 96-nt long exon 5. Out of four two-exon circRNAs we observed, C3-4 was the most abundantly expressed in all cell types we examined (Figure 2B). C3-4 was also one of the most abundant type 1 circRNAs, suggesting that backsplicing of the 5′ss of exon 4 with the 3′ss of exon 3 is a frequent event. Interestingly, the predicted secondary structure of intron 4 revealed very strong internal stems stretching over a ∼900-nt region (Supplementary Figure S5). It is likely that this structure facilitates backsplicing. Out of five three-exon circRNAs we observed, C2B-3-4 was the most abundantly expressed. The relative expression of C2B-3-4 was similar to that we observed for C3-4 (Figure 2B, lanes 9–16), suggesting that the 5′ss of exon 4 backspliced with the 3′ss of exons 2B and 3 with similar efficiency. The predicted secondary structure of a transcript in which introns 2B and 3 have been removed revealed internal stems formed between complementary sequences in intron 2A and exon 4 (Supplementary Figure S6). This structure brings the 5′ss of exon 4 in close proximity to the 3′ss of exons 2B and likely facilitates backsplicing, leading to the generation of C2B-3-4. Between two circRNAs with four exons we captured, C2A-2B-3-4 showed high expression, particularly when amplified using divergent primers annealing to exon 2A (Figure 2B). Interestingly, the 3′-end of intron 1 seemed to base pair extensively with exon 4 when the intervening introns have been removed (Supplementary Figure S7). This arrangement brings the 5′ss of exon 4 closer to the 3′ss of exon 2A, facilitating generation of C2A-2B-3-4. However, levels of C2A-2B-3-4 appeared substantially lower when amplified using divergent primers annealing to exons 2B and 3. This could be due to poor annealing of RT primer to exons 2B and 3 owing to rigid RNA structures. We also detected two circRNAs with five exons and one circRNA with six exons (Figure 2A). However, these circRNAs were expressed at low levels in all cell types we tested (Figure 2B).

Currently it is not known if the Alu-rich intron 1, the largest intron of *SMN*, harbors a cryptic exon. In this study, we identified four type 1 circRNAs encompassing cryptic exons from intron 1 (Figures 1 and 2, Supplementary Figures S2 and S4). One of these exons, I1(NE3-33), was found in two circRNAs, suggesting its frequent usage. Additional two type 1 circRNAs were generated using a novel 3′ss within exon 1 (Figure 2, Supplementary Figure S4). We identified two circRNAs harboring a truncated exon 3 (Figure 2B, lanes 14–15, Supplementary Figure S8). In one type 2 circRNA a novel 5′ss within exon 6 was used to backsplice to exon 2A. C5-6-7-8A-9tr1, a type 3 circRNA, contained a truncated exon 8 due to usage of a cryptic 5′ss (GUAACU) at the 142^nd^ position of this exon (Figure 2, Supplementary Figure S9). We call this truncated exon 8 as exon 8A. C5-6-7-8A-9tr1 also incorporated exon 9tr1, a truncated version of the novel exon 9 downstream of exon 8 (described later). We observed several type 1 circRNAs with truncated exons 1, 2A, 2B, 3 and 4 (Supplementary Figure S9). Other surprising findings were the identification of two type 4 circRNAs generated by trans-splicing with sequences from *SERF1* and *ERBIN*, respectively (Supplementary Figures S4 and S10). Both of these type 4 circRNAs used the 3′ss of exon 2A and the 5′ss of the foreign exons.

### Inclusion of novel intergenic exons expands the diversity of *SMN* circRNAs

To identify circRNAs produced by the 3′-terminal exons of *SMN*, we generated cDNAs using RNase R-treated RNAs and RT primers annealing to exons 6, 6B, 7 and 8. We then performed PCR employing divergent primers annealing to these exons. Here again, we conducted these experiments in four cell lines to capture the broad spectrum of circRNAs generated in a cell-specific manner. Our results revealed 28 circRNAs harboring the 3′-terminal exons, 22 of which belonged to the type 3 category (Figure 3, Supplementary Table S3). The remaining six circRNAs belonged to the type 2 category. Surprisingly, 15 circRNAs incorporated one or two of four novel exons derived from intergenic sequences downstream of exon 8. We named these exons as 9, 10, 11 and 12. Out of four intergenic exons, only exon 9 was derived from an Alu element (Figure 1, Supplementary Figure S3). Hence, exon 9 becomes the second *SMN* exon after exon 6B to be derived from an Alu element. In contrast to exon 6B that maps to the left arm of the antisense sequence of an Alu element, exon 9 maps to the right arm of the antisense sequence of an Alu element (Figure 1). Exons 10 and 11 are derived from DNA and LTR repeats, respectively (Figure 1, Supplementary Figure S3). In several instances, we observed circRNAs incorporating a truncated version of exon 9 (9tr1) due to usage of a cryptic 3′ss located 4-nt downstream of the 3′ss of exon 9. Interestingly, we recorded more circRNAs with exon 9tr1 than with exon 9, suggesting that the 3′ss of exon 9tr1 is stronger than the 3′ss of exon 9. This matches the results of splice sites strength scoring, according to which the 3′ss of exon 9tr1 is stronger than the 3′ss of exon 9 (Supplementary Table S6). We captured 9, 7, 3 and 1 circRNAs harboring exons 9/9tr1, 10, 11 and 12, respectively, suggesting that early intergenic exons were more frequently incorporated into circRNAs than later intergenic exons. It is likely due to high probability of transcription termination as RNA pol II moves into the intergenic region. Transcription of the intergenic region until exon 12 did not necessarily guarantee inclusion of all preceding intergenic exons. For instance, C7-8A-11-12 that harbored exons 11 and 12 lacked intergenic exons 9 and 10. Also, we did not capture any circRNA incorporating both exons 10 and 11, suggesting that these two intergenic exons are included in a mutually exclusive manner. Notably, we did not capture any circRNA harboring all four intergenic exons. We observed two circRNAs that used cryptic 5′ss within intron 7 (Figure 3, Supplementary Figure S9). Interestingly, one of these cryptic 5′ss (GUCUGC) is located 4-nt downstream of the natural 5′ss of exon 7. This cryptic site overlaps an inhibitory RNA structure as well as an 8-nucleotide GC-rich motif (CUGCCAGC), sequestration of which promotes *SMN2* exon 7 inclusion (59–61). The other cryptic 5′ss used was Cr1, located 23-nt downstream of the natural 5′ss of exon 7. Cr1 is located upstream of the previously reported TIA1 binding site and shows the highest potential to be activated by an eU1 when the native 5′ss of exon 7 is mutated (53,62). While most type 3 circRNAs incorporated 142-nt long exon 8A, we also observed circRNAs with varied lengths of exon 8. Of these, only two circRNAs utilized canonical U2 type (GU/AG) 5′ss located at 20- and 25-nt downstream of the 5′ss of exon 8A. Other extremely low abundant circRNAs appeared to use unusual 5′ss within exon 8 (Supplementary Figure S9). However, manifestation of these products could be an artefacts of the background amplification.

We observed six circRNAs harboring exon 6B (Figure 3A). Four of these circRNAs also contained exons 6 and 7. Generation of these circRNAs confirmed that both the 5′ and 3′ss of exon 6B are used for circularization. Exons 6, 7 and 8 provided the 3′ss for the generation of 16, 4 and 2 type 3 circRNAs, respectively. The 3′ss of exons 4 or earlier exons were not used for the generation of type 3 circRNAs. Overall, our experimental approach of employment of divergent primers annealing to exons 6, 6B, 7 and 8A cross validated the generation of various circRNAs. In particular, our results confirmed C6-7-8A as the most abundant type 3 circRNA. Interestingly, computational folding of the region spanning from intron 5 to exon 8 revealed a predicted secondary structure that brings the 5′ss of exon 8A in close proximity of 3′ss of exon 6 when all of the intervening introns are removed (Supplementary Figure S11). Such a structural arrangement is likely conducive for the generation of C6-7-8A. Analysis of 10 clones from HeLa cells confirmed that both *SMN1* and *SMN2* generate C6-7-8A with equal frequency. Except for exon 10-containing circRNAs that were preferentially expressed from *SMN1*, all other type 3 circRNAs we analyzed were generated from both *SMN1* and *SMN2* (not shown). We observed five bands bearing a variety of different circRNAs of similar sizes, which we named as Group (Grp) 1–5 (Figure 3B). In GM03813 cells, Grp 1 and Grp 3, which include a mixture of exon 6B- and exon 10-containing circRNAs, were present at noticeably lower levels.

While PCR amplification of the RNase R-treated samples employing divergent primers is the most commonly accepted practice for the detection of circRNAs (15,63), this approach is not foolproof due to artefacts associated with the reverse transcription and PCR amplification steps. Hence, we used RNase protection assays (RPAs) to independently validate the nature of the backsplicing events used to generate the most abundant circRNAs of *SMN*. We chose RPA because of its high precision and increased sensitivity compared to northern blotting (64,65). In an initial survey of all major backsplice junctions as well as linear *SMN*, we were able to identify strong signal using probes complementary to the exons 2A-2B junction (identifying linear *SMN*), as well as the backsplicing junctions between exon 4 and exons 2A, 2B and 3, which recognize the three most abundant type 1 circRNAs, C2A-2B-3-4, C2B-3-4 and C3-4 (Supplementary Figure S12A, lanes 1–12). We also identified weak signal using a probe complementary to the backsplicing junction between exon 6 and exon 5, detecting C5-6 (Supplementary Figure S12A, lanes 13–15). We did not observe any signal from RPA using probes complementary to any of the type 3 circRNA backsplicing junctions, suggesting their extremely low abundance (Supplementary Figure S12A, lanes 16–24). Also, identification of C6-7-8A was complicated by the presence of a nonspecific band of the similar size (Supplementary Figure S12A, lanes 17 and 18). Of note, an Alu element that promotes generation of a circRNA can in principle also favor the generation of a trans-backspliced linear RNA involving the similar splice site pairing (36). Supporting the occurrence of the trans-backsplicing events, RNase R-treated samples consistently showed reduction in the intensity of the bands amplified using divergent primers (not shown). However, available methods do not reliably provide a quantitative estimate of the backsplicing and trans-backsplicing events involving specific splice site pairings. To confirm the backsplicing junctions in the three most abundantly expressed circRNAs (C2A-2B-3-4, C2B-3-4 and C3-4), we performed RPAs in RNase R-treated samples. We expected low signal in RNase R-treated samples due to several reasons, including removal of the trans-backspliced linear transcripts, nicking of circRNAs during RNA isolation and the presence of the contaminating RNases in the commercially available RNase R preparation. Indeed, the residual signal after RNase R digestion was greatly reduced compared to the mock-treated samples. However, the signal for each of the backsplicing junctions was greater than that for the linear *SMN* (Supplementary Figure S12B), confirming the presence of the backsplicing junctions leading to the generation of the all three abundantly expressed *SMN* circRNAs. In addition, the probe used for detection of linear *SMN* can also anneal to C2A-2B-3-4, meaning that the amount of residual linear RNA left after RNase R treatment is likely to be overestimated.

### Novel intergenic exons are incorporated into linear transcripts of *SMN*

**Figure 4.**
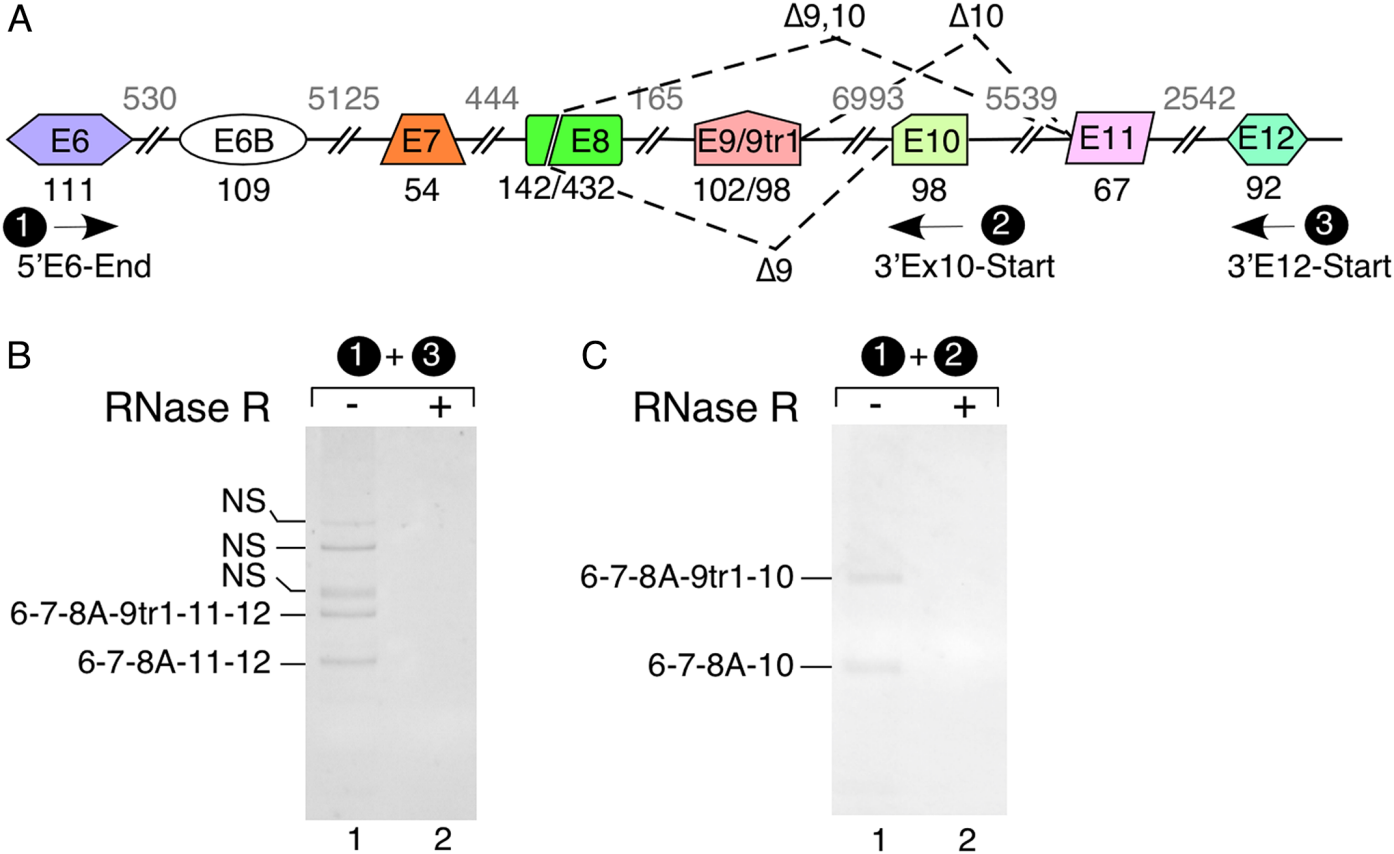
Linear *SMN* transcripts showing inclusion of novel exons 9, 10, 11 and 12. (**A**) An overview of the 3′ portion of the *SMN* pre-mRNA. Splicing events are indicated by dotted lines. Exons are depicted as colored shapes, and introns are shown as lines/broken lines. Exon sizes are indicated by numbers in black below exons and intron sizes are indicated by numbers in gray above introns. Names, assigned numbers and the locations of primers used to amplify linear *SMN* transcripts are presented. ‘Δ’ indicates *SMN* transcripts with skipped exon(s). (**B, C**) Detection of novel exons 9, 10, 11 and 12 without (−) and with (+) RNase R treatment. Primers used are shown at the top of each gel. After purification of total RNA, we isolated intact poly(A)^+^ RNA using magnetic beads and generated cDNA employing oligo(dT)_12–18_ primer. The identities of bands marked on the left of each gel were determined by cloning and sequencing. Abbreviations: NS, non-specific.

To validate that intergenic exons are incorporated into the linear polyadenylated transcripts of *SMN*, we conducted a series of experiments. We first enriched polyadenylated transcripts from total RNA of HeLa cells using oligo-dT beads and then generated cDNA using an oligo(dT)_12–18_ primer. As a control, we performed a parallel experiment using RNase R-treated sample. We subsequently performed PCR using forward and reverse primers annealing to exons 6 and 12, respectively (Figure 4A). We determined the identities of the amplified products by sequencing. Confirming incorporation of intergenic sequences in the linear transcripts, we observed two equally intense *SMN*-specific bands in the RNase R-untreated sample (Figure 4B). One of these bands was generated by skipping of exon 10 of *SMN1*, whereas the other band was generated by skipping of exons 9 and 10 of *SMN1*. To further confirm that exon 10 is also included into the linear transcripts of *SMN*, we repeated the above experiment using forward and reverse PCR primers annealing to exons 6 and 10, respectively. Here again, we observed two equally intense bands in the RNase R-untreated sample (Figure 4C). One of these bands was generated by inclusion of intergenic exon 9tr1 of *SMN1*, whereas the other band was generated by skipping of exon 9/9tr1 of *SMN1*. Although each of the sequences derived from linear transcripts that we analyzed came from *SMN1*, we originally observed circRNAs containing exons 11 and 12 in GM03813 cells, which only contain *SMN2*. Thus, we conclude that both *SMN1* and *SMN2* are capable of utilizing these exons. Our results confirmed incorporation of all intergenic exons of *SMN* into linear transcripts.

### *SMN* circRNAs are universally expressed in human tissues

**Figure 5.**
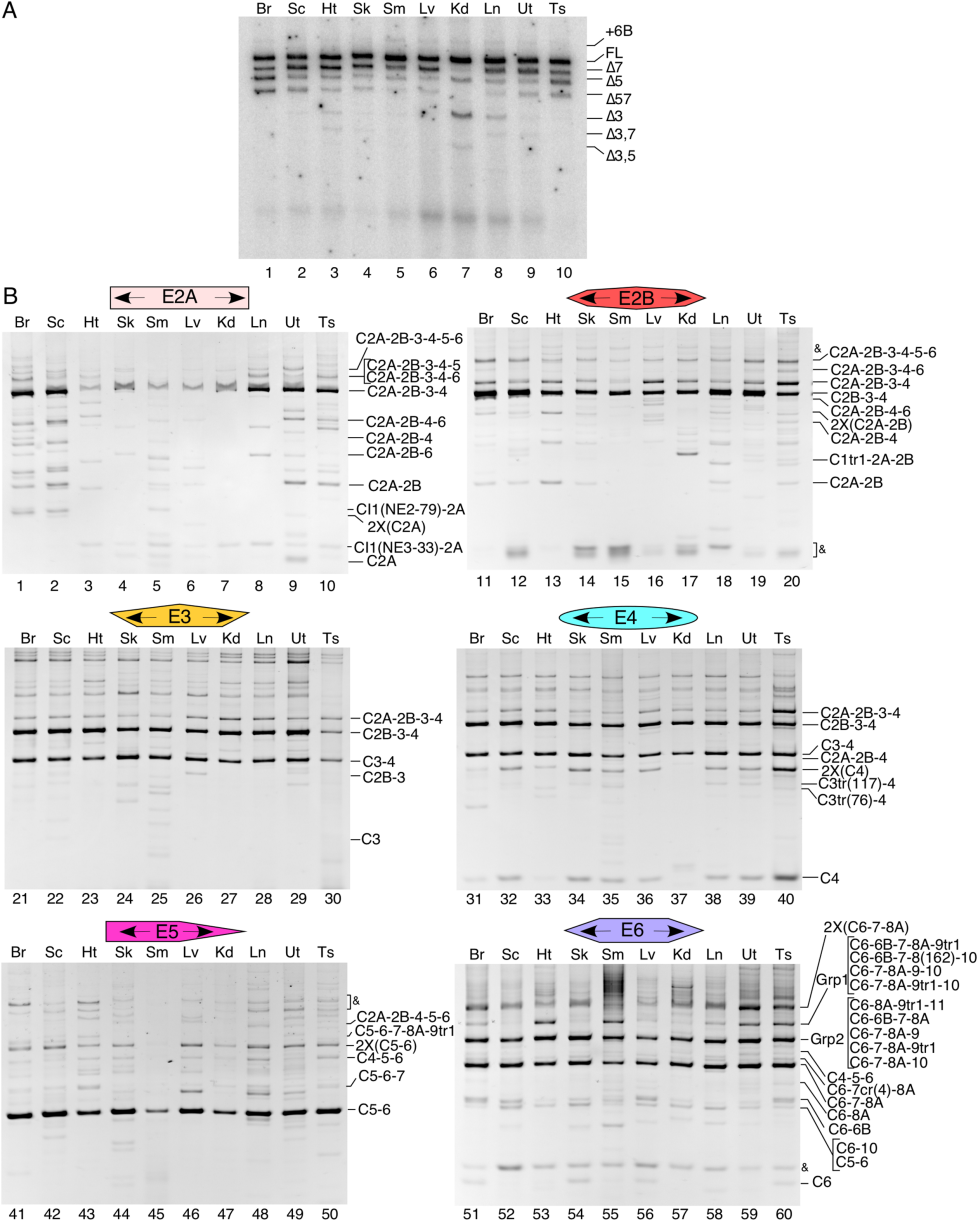
*SMN* circRNA production in human tissues. (**A**) MESDA depicting the splicing pattern of linear *SMN* pre-mRNA in different human tissues. Band identities are given on the right side of the gel. ‘Δ’ indicates *SMN* transcripts with skipped exon(s). ‘+6B’ indicates a full-length *SMN* transcript that also contains Alu-derived exon 6B. Tissue type is given at the top of each lane. Abbreviations: Br, brain; Sc, spinal cord; Ht, heart; Sk, skeletal muscle; Sm, smooth muscle; Lv, liver; Kd, kidney; Ln, lung; Ut, uterus; Ts, testis. (**B**) Ethidium bromide stained gels of divergent RT-PCR using human-tissue-derived RNA. Labeling is the same as in Figure 2 and in (A).

To determine the expression of *SMN* circRNAs in vivo, we examined 10 human tissues including brain, spinal cord, heart, skeletal muscle, smooth muscle, liver, kidney, lung, uterus and testis. We prepared cDNAs using RNase R-treated RNAs as template and RT primers annealing to exons 2A, 2B, 3, 4, 5 and 6. We amplified circRNAs by PCR employing divergent primers annealing to the above exons. Our approach was designed to capture the entire spectrum of circRNAs. Consistent with the results observed with the cultured cells, C2B-3-4 and C3-4 were the most abundant type 1 circRNAs generated in all tissues examined (Figure 5B, lanes 21–40). Similar to the results obtained with the cultured cells, C6-7-8A was the most abundant type 3 circRNA generated in all tissues examined (Figure 5B, lanes 51–60). These results validated the universal usage of the novel 5′ss within exon 8 and incorporation of the truncated exon 8A into circRNAs. Overall, our findings confirmed that C2B-3-4, C3-4 and C6-7-8A are universally expressed and constitute the most abundant circRNAs generated by *SMN*. Our results also confirmed expression of several low abundance circRNAs including those generated using novel cryptic 3′ss within intron 1 and exon 1 (Figure 5B). Similar to the observations we made using cultured cells, we captured differences in the expression of a handful of circRNAs when amplified using RT/PCR primers annealing to different exons. For instance, C5-6, a type 2 circRNA, was expressed >5-fold higher than C4-5-6 in all tissues when examined using exon 5-specific RT/PCR primers. However, the expression of C5-6 was comparable to that of C4-5-6 when examined using exon 6-specific RT/PCR primers. Here again, we attribute these differences to the different structural contexts of a given exon in different circRNAs. We captured tissue-specific differences in the expression of various low abundance *SMN* circRNAs. For instance, we observed enhanced expression of C4 in testis compared to all other tissues. Interestingly, C4 appeared to be absent in kidney. C2A-2B-3-4 showed high expression in testis based on exon 2B- and exon 4-specific amplification strategies. However, similarly to cultured cells, in human tissues C2A-2B-3-4 was poorly amplified using exon 3-specific primers.

Similar to the results obtained using cultured cells in which we observed cell-type specific amplification of Grp1 circRNAs (Figure 3), we observed poor amplification of the Grp1 population in brain, spinal cord, skeletal muscle, liver, kidney and lung (Figure 5B). However, the Grp1 circRNAs were amplified better in heart, smooth muscle, uterus and testis (Figure 5B). While these results confirmed inclusion of the intergenic exons into *SMN* circRNAs, they also suggested tissue-specific regulation of transcription of the intergenic region. Similar to the results we observed with the cultured cells (Figure 3), the Grp2 circRNAs were university expressed in all ten tissues examined (Figure 5B).

### Mouse *Smn* generates a distinct set of circRNAs

**Figure 6.**
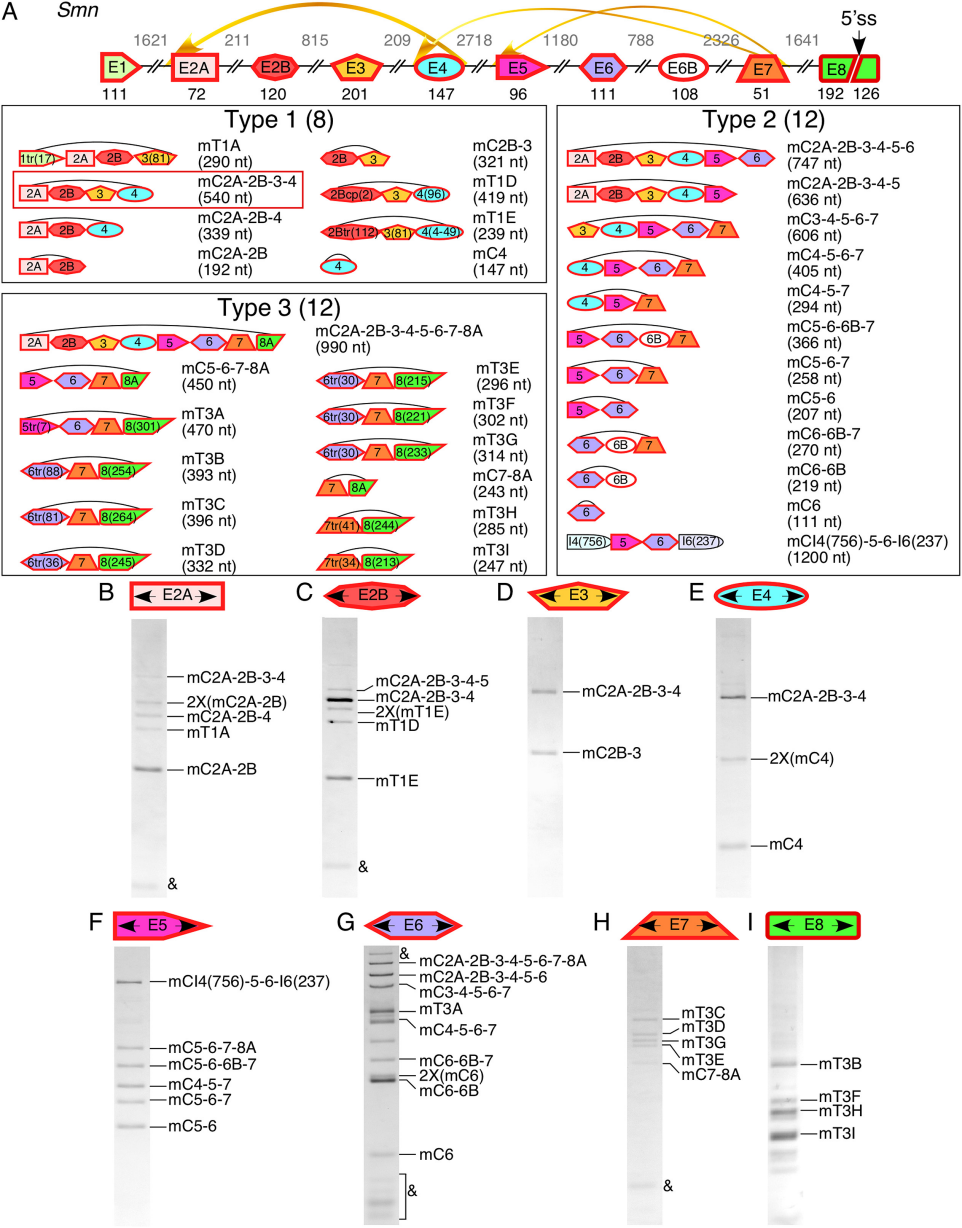
Exons of mouse *Smn* are involved in formation of a wide variety of circRNAs. (**A**) Top panel: A schematic presentation of the mouse *Smn* locus. Exons are depicted by colored shapes with red outlines, and introns are shown as lines/broken lines. Exon sizes are indicated by numbers in black below exons and intron sizes are indicated by numbers in gray above introns. The location of a novel 5′ss within exon 8 is shown with a black arrow. Curved yellow arrows represent backsplicing events, their thickness corresponds to the estimated prevalence of each event. Lower panels: An overview of the identified circRNAs with their sizes given in nucleotides. The most abundant circRNA is boxed in red. (**B**–**I**) Ethidium bromide stained gels of divergent RT-PCR products. The exon to which each divergent primer pair anneals is given at the top of each gel. Band identities marked on the right side of each gel were determined by cloning and sequencing. Nomenclature of circRNA is the same as in Figure 2, with the exception that all circRNAs are labeled with ‘m’ to denote that they are derived from mouse cells. ‘&’ indicates an artefact.

Rodent specific SINEs including inverted B1 elements have been implicated in the promotion of circRNA formation (32). Unlike the high abundance of primate-specific SINEs present within human *SMN*, the occurrence of SINEs in mouse *Smn* is low (Supplementary Figure S13). To determine whether mouse *Smn* generates circRNAs, we used mouse motor-neuron-like NSC-34 cells. We prepared cDNA libraries using RNase R-treated RNA as a template and RT primers annealing to all internal exons as well as exon 8, which is the last annotated exon. We then performed PCR employing divergent primers annealing to all internal exons as well as in exon 8. For clarity, we adopted a nomenclature in which we used prefix ‘m’ for mouse circRNAs. As expected, we captured a less diverse repertoire of mouse *Smn* circRNAs as compared to those produced by human *SMN*. For example, compared to 16 type 1 circRNAs generated by human *SMN*, we observed only five type 1 circRNAs generated by mouse *Smn* utilizing canonical splice sites (Figure 6). We observed an additional three candidate circRNAs, mT1A, mT1D and mT1E, that appeared to use unusual splice sites for backsplicing (Figure 6, Supplementary Figure S14). It is likely that RT slippage led to the deletion of the backsplicing junction of these three circRNAs. Interestingly, five type 1 circRNAs (mC2A-2B, mC2A-2B-4, mC2A-2B-3-4, mC2B-3 and mC4) were similar to those generated by human *SMN*. Out of these circRNAs, mC2A-2B-3-4 was the most abundantly expressed (Figure 6). Similar to human *SMN*, the predicted secondary structure involved RNA-RNA interaction between intron 1 and exon 4 (Supplementary Figure S15). Such an arrangement is likely to facilitate backsplicing between the 5′ ss of exon 4 and the 3′ ss of exon 2A. Unlike human *SMN* that produces C2B-3-4 and C3-4 as the major circRNAs, we did not detect analogous circRNAs generated by mouse *Smn*. We attribute this difference to the complete absence of SINEs within introns 2A and 2B of mouse *Smn* (Supplementary Figure S13).

We detected twelve type 2 circRNAs generated by mouse *Smn* (Figure 6). Only five of these circRNAs (mC2A-2B-3-4-5-6, mC2A-2B-3-4-5, mC5-6, mC5-6-7 and mC6) were similar to those generated by human *SMN*. Interestingly, three circRNAs incorporated a novel exon derived from an antisense strand of an ID4 element located within intron 6 (Supplementary Figure S16). We term this 108-nt long novel exon as exon 6B, although it should be noted that it shares no resemblance to that of human *SMN* exon 6B (Supplementary Figure S16). We confirmed that mouse *Smn* exon 6B is also incorporated into linear transcripts (Supplementary Figure S16). In contrast to human *SMN* that generated only two circRNAs (C5-6-7 and C6B-7) using the 5′ss of exon 7, about half of the type 2 circRNAs of mouse *Smn* were generated using the 5′ss of exon 7. We detected three type 3 circRNAs (mC2A-2B-3-4-5-6-7-8A, mC5-6-7-8A and mC7-8A) that incorporated a truncated exon 8 that we term exon 8A. Inclusion of exon 8A was facilitated by activation of a novel 5′ss (GUGCGU) at the 192nd position of exon 8. Of note, exon 8A of mouse *Smn* bears no resemblance to exon 8 of human *SMN*. Also, the score of the 5′ss of mouse *Smn* exon 8A was lower than the human *SMN* exon 8A (Supplementary Tables S6 and S7). Unlike in humans, where the longest circRNAs incorporated 6 exons (C2A-2B-3-4-5-6, C5-6-6B-7-8A-9tr1 and C6-6B-7-8A-9tr1-10), in mouse NSC-34 cells we identified circRNA that contained eight exons (mC2A-2B-3-4-5-6-7-8A): all internal ones plus exon 8A (Figure 6G). Importantly, mouse *Smn* did not produce circRNAs incorporating novel exons derived from the intergenic region. We detected nine extremely low abundant circRNAs that used unusual splice sites within exon 8 (Supplementary Figure S14). However, appearance of these products could be an artefact of background amplification.

Interestingly, we also identified one circRNA, mCI4(754)-5-6-I6(237) which incorporated exons 5 and 6, as well as partial introns 4 and 6 immediately upstream and downstream from them, respectively (Figure 6F). We did not detect any canonical splice site for backsplicing that likely generates this product. Analysis revealed deletion of sequences between a common 7-nt motif, (CUGGUCU), that was present in both introns 4 and 6 (Supplementary Figure S17). It is likely that mCI4(754)-5-6-I6(237) is generated from a lariat intermediate using the 5′ss of exon 4 and a branch point in intron 6. We speculate that a unique structural context brings the common 7-nt motif in close proximity, allowing RT slippage (Supplementary Figure S17).

### Depletion of DHX9 triggers perturbations of *SMN* pre-mRNA splicing and circRNA generation

**Figure 7.**
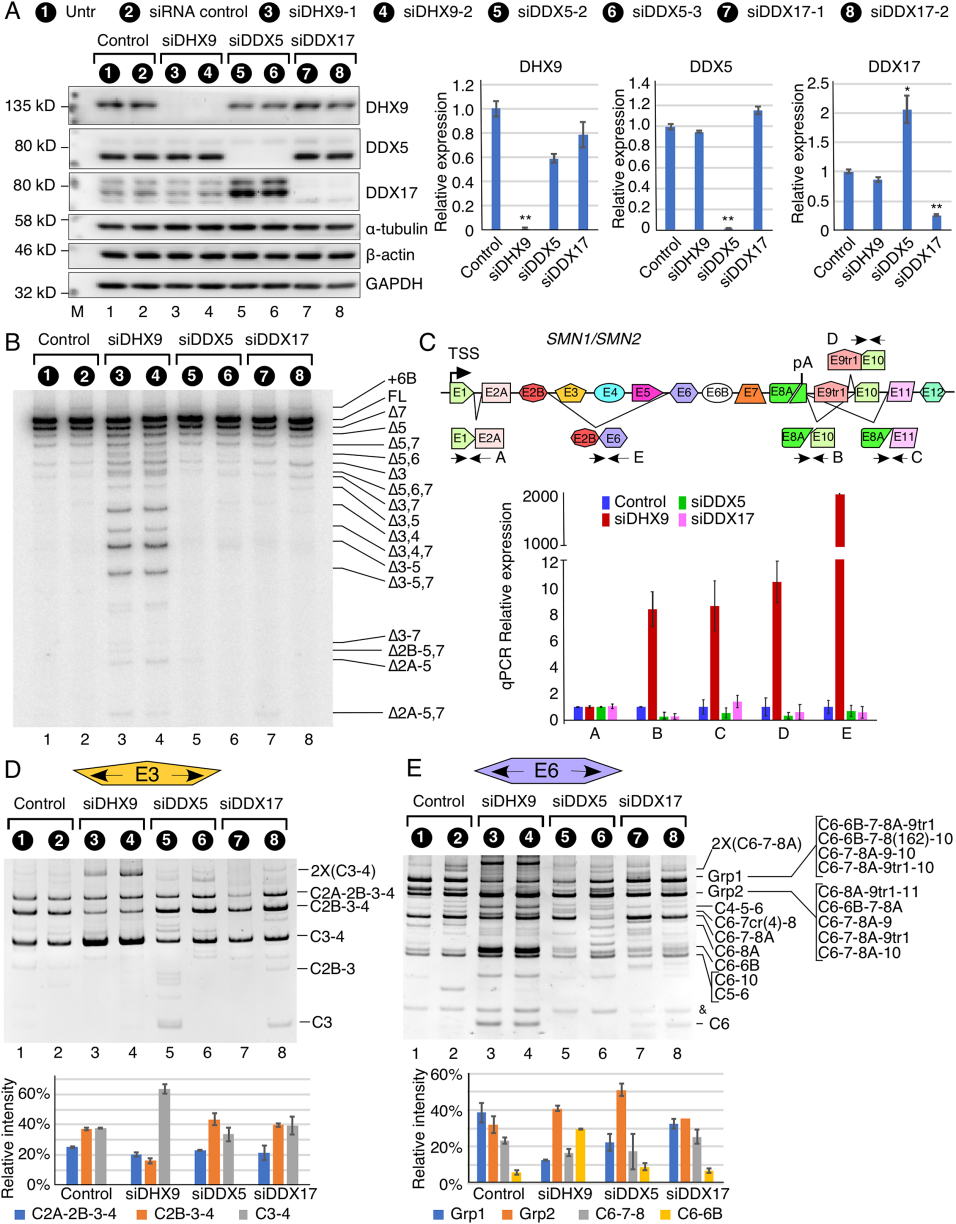
Knockdown of DHX9 impacts specific circRNAs formed by *SMN*. (**A**) Left panel: Western blots indicating levels of DHX9, DDX5 and DDX17 with and without siRNA transfection. α-tubulin, β-actin and GAPDH are used as loading controls. Sample treatment is indicated at the top of the panel. Antibody used is indicated at the right and nearby molecular weight markers are indicated at the left. Right panel: quantification of western blot results. For each band, background signal was subtracted and then signal was normalized by β-actin. Quantification and statistical analysis was performed on two replicates using two independent siRNAs targeting each gene (*n* = 4). Error bars represent standard error of the mean (SEM). *P < 0.05, **P<0.01 compared to untransfected and siRNA control samples. (**B**) MESDA depicting the splicing pattern of the linear *SMN* pre-mRNA. Treatment type is indicated at the top of the gel. Band identities are marked on the right side of the gel. ‘Δ’ indicates *SMN* transcripts with skipped exon(s). (**C**) Diagrammatic representation of exon/intron organization and splicing of *SMN1/SMN2*. Exons are depicted as colored boxes, and introns are shown as lines/broken lines. Transcriptional start site (TSS), polyA (pA) and location of primers used for qPCR are indicated. (**D**) Top panel: Ethidium bromide stained gel of divergent RT-PCR using primers annealing to exon 3, which produces mostly type 1 circRNAs. Treatment type is indicated at the top of the gel. Band identities are marked on the right side of the gel. Bottom panel: Quantification of the three most predominant bands from RT-PCR. Error bars represent SEM. (**E**) Top panel: Ethidium bromide stained gel of divergent RT-PCR using primers annealing to exon 6, which produces mostly type 3 circRNAs. Treatment type is indicated at the top of the gel. Band identities are marked on the right side of the gel. Bottom panel: Quantification of the four most predominant bands from RT-PCR. Error bars represent SEM.

DHX9, a member of the DEAH-containing family of RNA helicases, specifically disrupts IAR-associated-double-stranded RNA structures (32). To find a potential correlation between splicing/backsplicing of various *SMN* exons and IAR-associated-double-stranded RNA structures, we monitored levels of linear and circular transcripts upon siRNA-mediated depletion of DHX9. In parallel, we also monitored levels of linear and circular transcripts upon siRNA-mediated depletion of DDX5 and DDX17. Of note, DDX5 and its paralog DDX17 are highly conserved DEAD box helicase family members that are known to modulate transcription, pre-mRNA splicing and miRNA biogenesis through structural rearrangement (66). We performed these experiments after confirming by western blot that proteins remain depleted at 96 hours post siRNA transfection. We used two different siRNAs per gene to make sure that the results are not skewed by any specific siRNA sequence. Indeed, we observed near identical results with both siRNAs against all three of the genes we examined. Depletion of DDX5 caused a significant upregulation of DDX17 (Figure 7A, lanes 5 and 6). However, we did not capture a reciprocal upregulation of DDX5 upon depletion of DDX17 (Figure 7A, lanes 7 and 8). Depletion of DHX9 had no effect on levels of DDX5 or DDX17 or vice versa (Figure 7A). We employed MESDA to capture the relative abundance of linear *SMN* transcripts generated by alternative splicing (Figure 7B). Depletion of DDX5 and DDX17 showed no appreciable effect on alternative splicing of *SMN* exons (Figure 7B, lanes 5–8). However, depletion of DHX9 showed an enhanced skipping of exon 3 with or without skipping of other exons (Figure 7B, lanes 3 and 4). The most unusual splice variants were Δ3,4, Δ3–5, Δ3,4,7, Δ3–5,7, Δ5–7, Δ2B-5,7 and Δ2A-5,7 indicating that DHX9 depletion caused a general perturbation of linear splicing of all internal exons of *SMN*.

To monitor the levels of *SMN* transcripts in helicase-depleted cells, we performed qPCR using various primer combinations (Figure 7C). Since we prepared cDNAs using DNase-treated RNAs as template and oligo(dT)_12–18_ as primer, our approach allowed amplification of all polyadenylated transcripts. Depletion of DHX9, DDX5 and DDX17 had no effect on levels of total *SMN* transcripts (Figure 7C). Consistent with the results of MESDA (Figure 7B), results of qPCR confirmed substantial increase in the levels of Δ3–5 transcripts in samples with DHX9 depletion but not in samples with the depletion of either DDX5 or DDX17 (Figure 7C). Interestingly, DHX9 depletion caused an increase in the levels of transcripts containing novel intergenic exons of *SMN* (Figure 7C). These results are consistent with the recent finding that DHX9 depletion causes readthrough downstream of the poly(A) signal (67).

For the assessment of *SMN* circRNAs impacted by depletion of DHX9, DDX5 and DDX17, we prepared cDNAs using RNase R-treated RNAs as template and RT primers annealing to exons 3 and 6. We then performed PCR employing divergent primer pairs annealing to each of these exons. While we expected variations in the levels of some circRNAs due to sample-specific sensitivity to RNase R treatment, we were able to capture significant changes in the expression of several circRNAs upon DHX9 depletion. Using divergent primers located in exon 3, we observed upregulation of C3-4 when DHX9 was knocked down (Figure 7D, lanes 3 and 4). This finding is consistent with the enhanced skipping of exons 3 and 4, since C3-4 could be generated from the lariat intermediate harboring skipped exons 3 and 4. Compared to C3-4, levels of C2B-3-4 were substantially downregulated upon depletion of DHX9 (Figure 7D, lanes 3 and 4). This result is anticipated based on the logic that the 5′ss of exon 4 competes with the 3′ss of exons 2B and 3 for the generation of C2B-3-4 and C3-4, respectively. In agreement with the enhanced skipping of exons 5 and 6, depletion of DHX9 produced increased expression of three type 3 circRNAs, C6-6B, C6-10 and C5-6 (Figure 7E, lanes 3 and 4). Depletion of DHX9 also trigged abnormally high expression of circRNAs harboring intergenic exons, especially the Grp2 circRNAs (Figure 7E, lanes 3 and 4). In order to determine whether the increase in Grp2 circRNAs was due to an increase in any specific events or whether it was a general increase, we sequenced 10 clones from control and DHX9 knockdown samples. In control samples, the Grp2 population was relatively evenly distributed between C6-6B-7-8 (four clones), C6-7-8A-9tr1 (four clones), and C6-7-8A-10 (two clones). In contrast, in DHX9 knockdown samples, 6 of the 10 clones were C6-7-8A-9tr1 and 1 was C6-7-8A-9, for a total of seven clones using the 5′ss of exon 9/9tr1 and the 3′ss of exon 6 for backsplicing.

### Effect of transcription elongation inhibition on generation of *SMN* circRNAs

**Figure 8.**
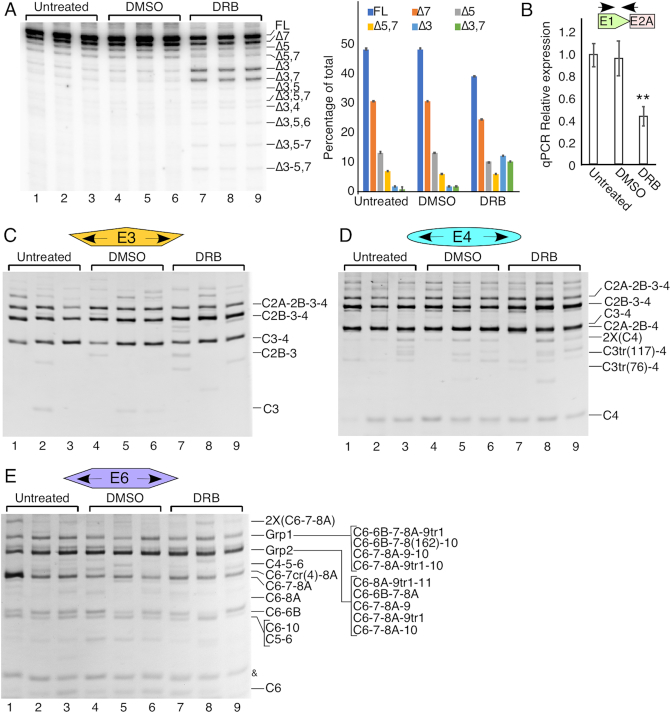
DRB treatment for 8 h impacts only linear transcripts of *SMN*. (**A**) Left panel: MESDA depicting splicing of the linear *SMN* pre-mRNA with and without DRB treatment. Treatment type is indicated at the top of the gel. Each treatment was performed in triplicate. Band identities are marked on the right side of the gel. ‘Δ’ indicates *SMN* transcripts with skipped exon(s). Right panel: quantification of relative band intensities. (**B**) Expression levels of total *SMN* transcripts relative to the mean of the untreated samples. Error bars represent SEM. **P < 0.01 compared to untreated samples. The location of primers used for qPCR is indicated. (**C**) Ethidium bromide stained gel of divergent RT-PCR using primers annealing to exon 3, which produces mostly type 1 circRNAs. Treatment type is indicated at the top of the gel. Band identities are marked on the right side of the gel. (**D**) Ethidium bromide stained gel of divergent RT-PCR using primers annealing to exon 4, which produces mostly type 1 circRNAs. Labeling is the same as in (C). (**E**) Ethidium bromide stained gel of divergent RT-PCR using primers annealing to exon 6, which produces mostly type 3 circRNAs. Labeling is the same as in (C).

DRB is one of the most widely used compounds to inhibit transcription elongation as well as to study coupling between transcription elongation and splicing (68–70). Depending upon the context, DRB has been found to promote both inclusion and skipping of exons (68–70). To examine the effect of transcription elongation inhibition on splicing of *SMN* exons, we treated HeLa cells with 80 μM DRB for four timepoints: 4, 8, 12 and 24 h. We then monitored the splicing of all internal exons of *SMN* employing MESDA. As expected, DRB treatment caused a noticeable reduction in the full-length *SMN* transcripts (Supplementary Figure S18). Surprisingly, DRB treatment also caused an increase in exon 3 skipping, suggesting a coupling between *SMN* transcription elongation rate and exon 3 splicing (Supplementary Figure S18). For subsequent analyses, we chose 8-hour duration of DRB treatment, at which point we observed ∼50% reduction in the full-length *SMN* transcripts and maximum skipping of exon 3 (Figure 8A and B). Results of MESDA at 8-hour DRB treatment revealed two prominent bands of near equal intensity corresponding to exon 3 skipping. One of these bands (top band) harbored exon 7, whereas, the other band (bottom band) lacked exon 7 (Figure 8A). We also detected faint bands corresponding to co-skipping of exon 3 with other exons (Figure 8A). Interestingly, while we observed an increase in the relative abundance of Δ3,5, and Δ3,7 transcripts, we noticed a reduction in the levels of Δ5 and of Δ7 transcripts (Figure 8A).

We next analyzed the relative abundance of *SMN* circRNAs in HeLa cells treated with 80 μM DRB for 8 h. In particular, we wanted to see if the levels of the exon 3-lacking circRNAs (C2B-4 and C2A-2B-4) were elevated in DRB-treated samples. We also expected reduction in the relative abundance of C3-4 and C2A-2B-3-4 transcripts. However, we observed no noticeable difference in the relative abundance of any of the *SMN* circRNAs upon DRB treatment (Figure 8C–E). These results suggested that the inhibition of transcription elongation did not disproportionally impact the splice site pairing for backsplicing. These findings are consistent with a recent report supporting that the generation of circRNAs is mostly a posttranscriptional event (71). We also employed an ISS-N1-targeting ASO (Anti-N1) to capture the effect of the enhanced inclusion of exon 7 on levels of various *SMN* circRNAs. We observed no appreciable change in the relative abundance of any *SMN* circRNAs upon ASO-mediated induction of exon 7 inclusion in both HeLa and GM03813 cells (Supplementary Figure S19). These results suggest that the splicing of exon 7 is not a limiting factor for the generation of type 3 circRNAs, most of which harbor exon 7.

### Type 1 circRNAs cross-regulate their own expressions

**Figure 9.**
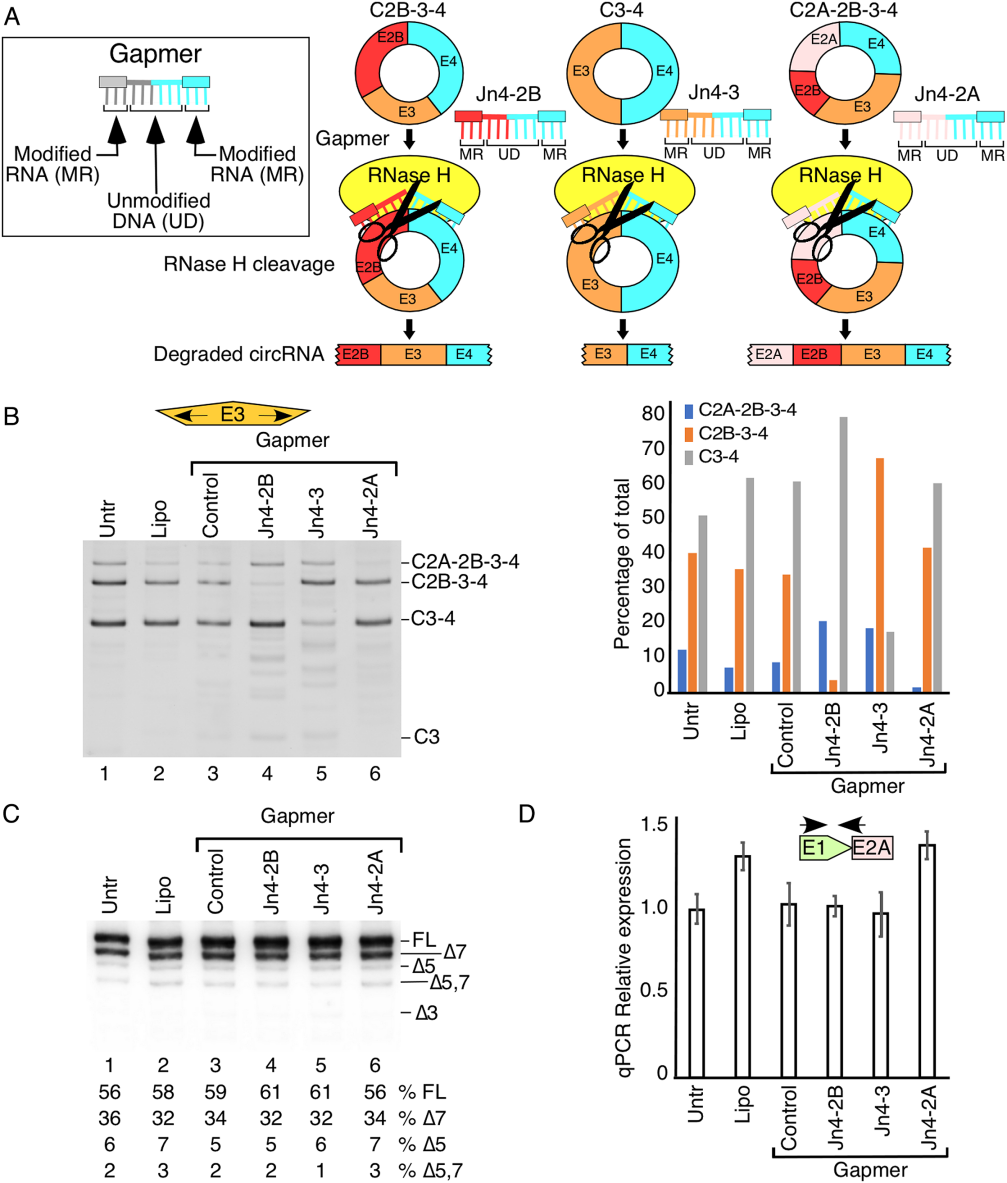
Knockdown of specific circRNAs using gapmers. (**A**) Diagrammatic representation of circRNA degradation caused by gapmers. Inset on top left shows design of a gapmer. Gapmer ASOs anneal to their respective targets and trigger degradation by an RNase H-mediated process. (**B**) Ethidium bromide stained gel of divergent RT-PCR using primers annealing to exon 3. Treatment/transfection type is indicated above each lane. Band identities are marked on the right side of the gel. Right panel: quantification of relative band intensities. (**C**) MESDA depicting the splicing pattern of linear *SMN* pre-mRNA. Band identities are marked on the right side of the gel. ‘Δ’ indicates *SMN* transcripts with skipped exon(s). (**D**) Expression levels of total *SMN* transcripts in different treatment. Expression levels are expressed relative to the mean of the untransfected (Untr) samples. Error bars represent SEM. The locations of primers used for qPCR are indicated by arrows. Abbreviations: Jn, Junction; Lipo, Lipofectamine 2000.

We next examined the effect of depletion of C2B-3-4, one of the most predominantly expressed circRNAs, on the expression of other type 1 circRNAs. We meticulously engineered a RNase H compatible gapmer ASO, Jn4-2B, that annealed to the exon 4-exon 2B junction (Figure 9A).

We then transfected HeLa cells with Jn4-2B to deplete C2B-3-4. For circRNA analysis, we used RNase R-treated total RNA as template followed by RT-PCR using divergent primers annealing to exon 3. As expected, Jn4-2B gapmer almost completely depleted C2B-3-4. A control gapmer ASO with scrambled sequence had no appreciable effect on the relative expression of C2B-3-4. Depletion of C2B-3-4 upregulated C3-4 and C2A-2B-3-4, although the ratio between C3-4 and C2A-2B-3-4 appeared to be unchanged (Figure 9B, lanes 3 and 4). We performed complementary experiments to assess the effect of the gapmer-assisted depletion of either C3-4 or C2A-2B-3-4 on other type 1 circRNAs. While depletion of C3-4 disproportionately upregulated C2B-3-4 (Figure 9B, lanes 3 and 5), depletion of C2A-2B-3-4 upregulated both C3-4 and C2B-3-4, although the ratio between C3-4 and C2B-3-4 remained unchanged (Figure 9B, lanes 3 and 6). Overall, our results suggest that the absence of a given type 1 circRNA has a stabilizing effect on other type 1 circRNAs. Alternatively, it is also possible that the presence of a given type 1 circRNA interferes with the generation of other type 1 circRNAs. We performed MESDA to examine if the depletion of one of the abundantly expressed type 1 circRNAs has any effect on the relative abundance of the linear *SMN* splice isoforms. Results of MESDA showed no appreciable effect of the depletion of C2B-3-4, C3-4 and C2A-2B-3-4 on any of the linear splice isoforms of *SMN* (Figure 9C). We observed no significant effect of the depletion of any of the above expressed type 1 circRNAs on the overall levels of the *SMN* transcripts (Figure 9D).

## DISCUSSION

The diversity and abundance of circRNAs present within the human transcriptome is currently beyond the realms of accurate prediction due to the non-colinear nature of circRNAs coupled with the unpredictable usage of novel splice sites to generate circRNAs. Thus, a tailored approach provides a powerful alternative to uncover the entire spectrum of gene-specific circRNAs. Employing such an approach, here we report the surprising diversity of exon-containing circRNAs generated by human *SMN* genes that code for SMN, an essential protein implicated in several housekeeping functions. While the coding sequence of *SMN* is highly conserved in mammals, frequent insertions of Alu elements in primates have introduced substantial differences in the intronic, promoter and intergenic regions of human *SMN* genes (Figure 1). Considering that IARs are strong drivers of circRNA generation (19,29–31), *SMN* offers an ideal system to test the higher limit of circRNAs generated by a single gene. We analyzed *SMN* circRNAs after treatment of total RNA with RNase R, which selectively removes linear transcripts. To maximize the chances of identification of most exon-containing circRNAs, we generated cDNA libraries using RT primers annealing to each internal exon followed by PCR using divergent primers annealing to their respective exons. To further enhance the chances of capturing the full spectrum of *SMN* circRNAs, we performed these experiments in multiple cell types and postmortem human tissues. We conclusively validated 53 *SMN* circRNAs belonging to four broad types based on the identities of the included exon(s). Among validated circRNAs, 16, 12, 23 and 2 belonged to types 1, 2, 3 and 4 categories, respectively (Figures 2 and 3). In addition to confirming the presence of all known exons in one or more circRNAs of *SMN*, our results uncovered several novel exons that were incorporated into *SMN* circRNAs. We also validated the expression of most *SMN* circRNAs in RNA samples from ten postmortem human tissues, including brain, spinal cord, heart, liver, lung, kidney, testis, uterus, and smooth and skeletal muscles (Figure 5).

The exceptional diversity of *SMN* circRNAs was facilitated by backsplicing employing canonical U2 type (GU/AG) splice sites of various exons. Our results suggested competition between neighboring and distant splice sites for the incorporation into circRNAs. Consistently, we identified circRNAs harboring as few as one exon and as many as six exons. C2A-2B-3-4, C2B-3-4 and C3-4 were the most abundant circRNAs detectable by RPA, an amplification-free approach (Supplementary Figure S12). The 5′ and the 3′ss used to produce C3-4 and C2A-2B-3-4 are separated by 413- and 4031-nt, respectively, suggesting that the distance between splice sites used for backsplicing did not play a significant role in the generation of the most abundant circRNAs. Supporting this argument, RNA structure and not sequence has been implicated as one of the major drivers for backsplicing (7). Generation of C2B-3-4 is expected to be facilitated by bringing the 3′ss of exon 2B closer to the 5′ss of exon 4 through an extensive complementarity between intron 2A and exon 4 (Supplementary Figure S6). Similarly, formation of C2A-2B-3-4 is likely aided by bringing the 3′ss of exon 2A closer to the 5′ss of exon 4 through an extensive complementarity between intron 1 and exon 4 (Supplementary Figure S7). With significance to the generation of C3-4, the predicted secondary structure encompasses extensive complementarity between introns 2B and 4 (Supplementary Figure S5). Consistent with transcription coupled splicing regulation (37), secondary structures may affect the outcome of backsplicing through pausing of the pol II during transcription elongation (38). However, there is also evidence to support that the generation of circRNAs is mostly a posttranscriptional event (71). In agreement with this hypothesis, inhibition of transcription elongation by DRB did not alter the relative abundance of *SMN* circRNAs (Figure 8).

We unexpectedly identified four circRNAs encompassing three novel cryptic exons derived from intron 1 (Supplementary Figure S2). Three of these circRNAs, CI1(NE2-79)-2A, CI1(NE1-98)-2A and CI1(NE3-33)-2A, were formed using the 5′ss of exon 2A, the first internal exon. These results underscored the mutually competing nature of dynamic structures formed by IARs, allowing pairing of a single 5′ss with different cryptic 3′ss within intron 1. Formation of these three circRNAs is likely facilitated by events that make the 5′ss of exon 2A available for pairing with the upstream 3′ss. The most probable among these events is C2B-3-4 generation, which cleaves and releases the transcript upstream of intron 2A. Interestingly, one of the cryptic exons, I1(NE3-33), located towards the 3′-end of intron 1, was incorporated into two different circRNAs, CI1(NE3-33)-2A and CI1(NE3-33)-2B-3-4. Formation of CI1(NE3-33)-2A-2B-3-4 suggested that the backsplicing of a cryptic exon within intron 1 is not limited to the 5′ss of the first internal exon. Our results also confirmed that exon 4 is the farthest exon to be backspliced with one of the cryptic exons within intron 1. We observed two type 1 circRNAs, C1tr1-2A and C1tr1-2A-2B, which used a novel 3′ss located close to the translation start site within the first exon (Figure 2, Supplementary Figure S9). Formation of C1tr1-2A-2B is possibly enabled by delayed removal of intron 2B in transcripts that have already undergone removal of introns 1 and 2A. Also, production of C1tr1-2A-2B is likely facilitated by C3-4 generation, which results in cleavage and release of the transcript upstream of intron 2B. In general, it is possible that the biogenesis of a vast majority of type 1 circRNAs is enhanced by the generation of type 3 circRNAs and vice versa. Such a mode of regulation of circRNA formation would be consistent with an efficient cellular machinery that is geared to produce multiple products from a single precursor RNA.

Among the most surprising findings reported in this study is the discovery of four intergenic exons, namely exons 9, 10, 11 and 12, located downstream of exon 8 (Figures 1 and 3, Supplementary Figure S3). Out of four intergenic exons, exon 9 is derived from an Alu element. Our results confirmed incorporation of each of these intergenic exons into both linear and circular transcripts. However, we did not capture any linear or circular transcript containing all four intergenic exons (Figures 3 and 4). Of note, since we could not validate the presence circRNAs harboring intergenic exons by RPA, we infer that they are expressed at very low levels under normal conditions (Supplementary Figure S12). Another surprising finding was the frequent incorporation of exon 8A into both circular and linear transcripts due to activation of a cryptic 5′ss at the 142nd position of exon 8 (Figures 3 and 4). We also identified extended versions of exon 8A, generated by less frequent activation of yet other cryptic sites at the 162^nd^ and the 167^th^ positions of exon 8. In addition, we observed several rare circRNAs harboring truncated sequences of exon 8. Generation of most of these rare circRNAs were enabled by employment of the highly unusual 5′ss throughout exon 8 (Supplementary Figure S9). While usage of such splice sites has been recently reported (72), it is also possible that the actual splice junction sequences are lost for some of these rare circRNAs due to slippage by reverse transcriptase. Of note, slippage by reverse transcriptase causes deletion of sequences between two near identical motifs (73). In most cases where we observed usage of unusual splice sites, we identified duplicate sequences (not shown). Future studies employing slippage-proof reverse transcriptases would address discrepancies with respect to the usage of the non-canonical splice sites.

Lariat intermediates harboring skipped exons enhance the chances of circRNA formation due to elimination of a large number of competing splice sites present within the linear transcript. Generation of circRNAs via lariat intermediate could be particularly feasible for exons with weak splice sites. The 5′ss of the 201-nt-long exon 3 is one such example. This site was used to produce a single, poorly detectable circRNA, C3. Considering exon 3 undergoes alternative splicing, it is likely that C3 is generated from the lariat intermediate harboring skipped exon 3. Similar to exon 3, exon 5 is also an alternatively spliced exon. However, this 96-nt-long exon was the only internal exon for which the 5′ss was not used for backsplicing. We hypothesize that it is due to the small size of exon 5, when it may not be feasible to generate a circRNA from a lariat intermediate. We captured two circRNAs, C6B-7 and C5-6-7, that used the 5′ss of yet another alternatively spliced *SMN* exon, exon 7. It is likely that C6B-7 is generated from lariat intermediate harboring skipped exons 6B and 7. We have recently shown that low levels of a transcript lacking exons 3, 4, 5, 6, 7 is generated in all tissues (74). Therefore, it is also possible that C5-6-7 is produced from a lariat intermediate harboring skipped exons 3, 4, 5, 6, 7.

For an assessment of whether human *SMN* circRNAs are evolutionarily conserved, we examined mouse *Smn* circRNAs expressed in the motor-neuron-like NSC-34 cells. Compared to human *SMN*, mouse *Smn* produces a lesser variety of circRNAs (Figure 6). This is expected due to the low SINE content of the mouse *Smn* and a lack of transcription elongation into the intergenic region. Similar to human *SMN*, each internal mouse *Smn* exon is present at least in one type of circRNA. Except for the identification of the novel *Smn* exon 6B, our results did not reveal inclusion of any cryptic exons into *Smn* circRNA. While mouse *Smn* exon 6B shares no homology with human *SMN* exon 6B, we confirmed that similar to human exon 6B, mouse exon 6B is incorporated in both circular and linear transcripts (Figure 6, Supplementary Figure S16). Although nature and abundance of most mouse *Smn* circRNAs were quite distinct from those expressed by human *SMN*, we observed several circRNAs that were expressed by both mouse and human genes, suggesting common regulatory mechanisms for the generation of these circRNAs. Consistently, the precursor of mC2A-2B-3-4, the most predominantly expressed *Smn* circRNA, shares several structural features with those of the precursor of human C2A-2B-3-4 (Supplementary Figures S7 and S15). In particular, in both there is an extensive base pairing between intron 1 and exon 4. Mouse and human exons 8, which encompass the 3′-end processing site including polyadenylation and cleavage motifs, share poor homology. However, mouse *Smn* just like human *SMN* generates circRNAs employing cryptic splice sites within exon 8. It is likely that delayed cleavage and polyadenylation promotes circRNA production through activation of these random cryptic splice sites within exon 8 (Supplementary Figures S9 and S14). Supporting this argument, a recent study suggests that inhibition of transcription termination via depletion of cleavage and polyadenylation machinery enhances circRNA production (75).

To gain mechanistic insight into the generation of circRNAs we depleted three RNA helicases, DHX9, DDX5 and DDX17. While DHX9 is known to specifically disrupt secondary structures associated with IARs (32), DDX5 and DDX17 disrupt and reorganize more diverse RNA structures (76). Our results of MESDA revealed a significant effect of depletion of DHX9 on splicing of various *SMN* exons. In particular, we observed skipping of many exons in different combinations. These results represent the first example of the effect of an Alu-associated helicase on regulation of *SMN* splicing. As expected, enhancement in the formation of secondary structures associated with IARs due to DHX9 depletion caused upregulation of a select group of circRNAs, including C3-4, C6, C5-6, C6-6B and C6-7-8A-9. Based on the pattern of skipped exons, we infer that the lariat intermediates harboring exons are the likely source of the increase in circRNA production upon DHX9 depletion. Interestingly, DHX9, also known as RNA helicase A (RHA), interacts with SMN and SMA-causing mutations of SMN disrupt the DHX9-SMN interaction (77). In addition, recent reports implicate the role of SMN and DHX9 in transcription termination and R-loop resolution (78–80). It would be interesting to see if the low SMN levels affecting R-loop resolution also impact generation of specific circRNAs of *SMN*. In addition to the aberrantly high expression of *SMN* circRNAs, depletion of DHX9 caused more than 5-fold increase in the readthrough of the polyadenylation site within exon 8 (Figure 7C). It remains to be seen if the enhanced production of the readthrough transcripts of *SMN* has any physiological significance. Unlike the case with DHX9, we did not observe any significant effect of depletion of DDX5 and DDX17 on linear or circular transcripts of *SMN*. This is likely due to the presence of other DEAD-box helicases with redundant functions.

Depletion experiments supported cross-regulation of expression and/or levels of various type 1 circRNAs. Exon 3 independently folds into rigid secondary structure due to high sequence complementarity within this exon (Supplementary Figures S5–S7). The independent folding of exon 3 is fully conserved in pre-mRNAs that generate C2B-3-4, C3-4 and C2A-2B-3-4 (Supplementary Figures S5–S7). It is likely that exon 3 present within any of the abundantly expressed type 1 circRNAs disrupts the intramolecular structure of exon 3 of *SMN* pre-mRNA through formation of the intermolecular structures between complementary sequences within exon 3. This could be one of the possible mechanisms by which levels of various type 1 circRNAs are cross-regulated. It is also likely that the interacting proteins stabilize type 1 circRNAs. In this scenario, proteins released upon depletion of one type 1 circRNA are likely to associate with other type 1 circRNAs and stabilize them.

Thus far, most functions of the *SMN* genes have been associated with the SMN protein and its isoforms (39,81). Two recent reports suggest a role for non-coding antisense transcripts generated by *SMN* (82,83). A vast repertoire of circRNAs generated by Alu-rich *SMN* reported in this study opens up a new frontier that has not yet been explored. Despite low abundance of individual circRNAs, the sum of all circRNAs is likely to account for a sizeable fraction of the total transcripts generated by *SMN*. Since the approach adopted in this study was tailored to identify only exon-containing circRNAs, the number and expression levels of intron-only-containing circRNAs of *SMN* remains to be explored. Furthermore, considering circRNAs sponge miRNAs and proteins as well as regulate transcription (4,5,7), our findings are likely to expand the role of *SMN* in global regulation of translation, transcription and macromolecular trafficking. Based on recent reports supporting the generation of short peptides by circRNAs (6), *SMN* has a potential to produce a vast array of peptides longer than 50 amino acids through translation of its diverse circRNAs. Exons 4 and 6 contain translation initiation codons and all internals exons except exons 6B and 7 lack translation termination codons. Future studies will determine if the initiation of translation within exon 4 or exon 6 generates large protein(s) from one or more of the abundant *SMN* circRNAs encompassing these exons. An overwhelming majority of type 3 circRNAs harbored exon 7. However, we did not find a direct correlation between an enhanced exon 7 inclusion and the relative abundance of type 3 circRNAs (Supplementary Figure S19). It likely that the type 3 circRNAs are generated mostly after exon 7 inclusion. Many SMA patients have deletions of *SMN1* exon 7 and/or exon 8 as well as mutations in splice sites that facilitates exon 7 skipping (39,53,84). These deletions and/or mutations will affect the generation of type 3 circRNAs, including the most predominant C6-7-8A. Further investigations will establish if the loss of C6-7-8A contributes towards SMA pathogenesis. *SMN* is an essential gene for survival of all organisms belonging to the animal kingdom. Notably, a large fraction of circRNAs generated by *SMN* are specific to primates. Hence, any role linked to these circRNAs will underscore their evolutionary significance as well as improve our understanding of *SMN* gene function in primates.

## DATA AVAILABILITY

All novel linear and circular RNAs sequences of human *SMN* and mouse *Smn* can be accessed through NCBI; accession numbers are listed in Supplementary Tables S2 and S3, respectively.

## Supplementary Material

Supplementary Data
