# Supplementary material for: Human Survival Motor Neuron genes generate a vast repertoire of circular RNAs
Source: Nucleic Acids Res. 2019 Jan 30;47(6):2884–905. doi: 10.1093/nar/gkz034 (PMC6451121; doi:10.1093/nar/gkz034)
Supplement: Supplementary Data [file gkz034_supplemental_files.pdf]

SUPPLEMENTARY DATA

Supplementary materials and methods

Antisense oligonucleotides transfection

ASOs were synthesized by Dharmacon Inc., which incorporated 2′-O-methyl modification and phosphorothioate modification of the backbone.Transient transfections of cells with ASOs were performed using Lipofectamine 2000 following the manufacturer's recommendations. Twenty four hours prior to transfection, GM03813 and HeLa cells were seeded as ~2.3×10^5 cells per one well and 0.5×10^6 cells per one well in six-well plates, respectively, so that they will reach ~80% confluency on the day of transfection. For each ASO transfection, ASOs were prepared with 6.25 μL Lipofectamine 2000 and then added to pre-plated cells so that the final concentration in the culture medium was 10 nM. Cells were collected in TRIzol reagent for total RNA isolation at 24 h after transfection.

RNase Protection Assay

To generate probes complementary to exon junctions, sequences immediately upstream and downstream of the junction of interest were cloned into the pUC19 vector in the reverse orientation along with a T7 polymerase promoter. Probes were transcribed from linearized plasmids using T7 polymerase (NEB) in the presence of 50 μCi [α-^{32}P]-UTP (Perkin-Elmer) and a limiting concentration of cold UTP (12 μM) per 20 μl transcription reaction. RNase protection was performed as previously described (62) using 20 μg HeLa RNA (treated or mock treated with RNase R) and 1×10^5 counts per minute of labeled antisense probe. Products were resolved on 5% denaturing Urea-PAGE and exposed to phosphor screens. Screens were scanned in using a FujiFilm FLA-5100 system and analyzed using MultiGauge software (Fuji).

Supplementary Figure legends

Supplementary Figure S1. Relative expression of *SMN*-derived circRNAs. (A) As an estimate of total linear *SMN*, an ethidium bromide stained gel of MESDA of *SMN* is shown. The number of PCR cycles each sample underwent is indicated at the top of the gel. (B) As an estimate of each circRNA produced by *SMN*, ethidium bromide stained gels of divergent PCR for each internal exon of *SMN* are shown. Primer locations and PCR cycle numbers are indicated at the top of each gel. Band labeling is the same as Figure 5.

Supplementary Figure S2. *SMN* genes produce 3 novel exons from intron 1. An overview of the genomic arrangement of the *SMN* genes is given. Previously identified exons are shown as colored shapes, introns as lines. Exon sizes are indicated by numbers in black below each exon box, intron sizes are indicated by numbers in grey above each intron. Within intron 1, selected sequences are shown. In particular, three newly identified exons are presented in uppercase letters in white boxes. For exon I1(NE1-98), 20 upstream and downstream bases are shown in lowercase letters. The entire region is derived from an LTR9D element and is colored green. For exon I1(NE1-76), the entire L1 sequence is given and indicated with colored arrows. An additional 10 bases downstream are derived from an AluSp element. For exon I1(NE3-33), which does not overlap with any repeat, 20 upstream and downstream bases are shown in lowercase letters. Numbers in grey above connecting lines indicate number of bases between each sequence within intron 1.

Supplementary Figure S3. *SMN* genes produce 4 novel exons from downstream intergenic space. An overview of the genomic arrangement of the *SMN* genes is shown. Exons are shown as colored shapes, introns as lines. Exon sizes are indicated by numbers in black below each exon box, intron sizes are indicated by number in grey above each intron. Selected sequences located downstream of exon 8 are shown. Numbers in grey shown above connecting lines indicate number of bases between each indicated sequence. Sequences of the newly identified exon 9, 10, 11 and 12 are shown in uppercase letters and are boxed and highlighted in different colors. The upstream and downstream intronic sequences are shown in lowercase letters. In the cases where novel exons overlap with repeat sequences (exons 9-11), the entire repeat sequence is given and indicated with colored arrows. For exon 12, which does not overlap with any repeat, 20 flanking bases on each side are given.

Supplementary Figure S4. Identities of additional PCR products derived from divergent amplification of exons 2A and 2B. (A-B) Larger versions of the first two panels of Figure 2B are shown. Additional weaker bands and PCR products with nonstandard (no GU or AG) backsplice sites are indicated. Asterisks indicate unidentified bands.

Supplementary Figure S5. Secondary structure of intron 2B through intron 4 supporting formation of C3-4. Secondary structure of the RNA region from intron 2B to intron 4 is predicted by mfold. 5′ss of exon 4 and 3′ss of exon 3 are brought together by base pairing between intron 4 and sequences from exons 3 and 4 and in the context of double-stranded RNA formed by AluY and AluSx1. The sequence of *SMN* exons 3 and 4 and relevant portions of introns 2B and 4 are given. Exonic sequences are shown as colored uppercase and are boxed. Intronic sequences are shown as lowercase letters or as lines. AluY and AluSx1 sequences are

highlighted. Red arrows indicate direction of Alu insertion. Numbering of nucleotides starts from the beginning of exon 3. The 5′ and 3′ splice sites are shown with turquoise colored arrows.

Supplementary Figure S6. Secondary structure of intron 2A through intron 4 supporting formation of C2B-3-4. Secondary structure of the RNA region from intron 2A to intron 4 is predicted by mfold. 5′ss of exon 4 and 3′ss of exon 2B are brought together by base pairing between intron 2A and exon 4. The sequence of *SMN* exons 2B, 3, 4 and adjacent introns 2A and 4 are given. Exonic sequences are shown as colored uppercase letters and are boxed. Intronic sequences are shown as lowercase letters or as lines. Numbering of nucleotides starts from the beginning of exon 2B. 5′ and 3′ splice sites are shown with turquoise colored arrows.

Supplementary Figure S7. Secondary structure of intron 1 through intron 4 supporting formation of C2A-2B-3-4. Secondary structure of the RNA region from intron 1 to intron 4 is predicted by mfold. 5′ss of exon 4 and 3′ss of exon 2A are brought together by base pairing between intron 1 and exons 2B, 3 and 4. The sequence of *SMN* exons 2A, 2B, 3, 4 and adjacent introns 1 and 4 are given. Exonic sequences are shown in colored uppercase letters and are boxed. Intronic sequences are shown in lowercase letters or as lines. Numbering of nucleotides starts from the beginning of exon 2A. 5′ and 3′ splice sites are shown with turquoise colored arrows.

Supplementary Figure S8. Backsplicing events involving novel splice sites identified in circRNAs (A) Novel splicing events identified in type 1 circRNAs. Genomic overviews of areas involved in circRNA formation plus one flanking exon on each side are shown. Previously identified exons are shown as colored shapes, introns as black lines. Exon sizes are indicated by numbers in black below each exon, intron sizes by numbers in gray above each intron. The backsplicing events for each circRNA utilizing novel splice sites is shown with colored arrows. Newly identified splice sites internal to known exons are indicated by a black vertical line marked by arrows, and 10 bases flanking each side are given. Novel exons within intron 1 are shown as white boxes and 10 bases flanking each side of the novel 5′ and 3′ splice sites are given. Exonic sequences are given in uppercase, intronic sequences lowercase. A base that was mutated compared to the NCBI annotated version are given in red. (B) Novel splicing events identified in type 2 circRNAs. Labeling and coloring are the same as in (A). (C) Novel splicing events identified in type 3 circRNAs. labeling and coloring are the same as in (A).

Supplementary Figure S9. Variants of human *SMN* exons identified in circRNAs. Each square contains the sequence of one exon with exon name given on the top left. The locations where the variants start or end are marked with arrows. Exon variants are labeled in black, whereas circRNAs bearing the variants are indicated in blue within square brackets. Black dots under the sequences mark 10-nt length.

Supplementary Figure S10. Trans-splicing events lead to circRNAs containing sequences from other genes. Diagrammatic representation of the hypothetical mechanism showing two type 4 circRNAs formation. Exons are depicted by colored boxes and introns by lines. *SMN* is shown with intron 2A removed and *ERBIN* is shown with intron 2 removed. Repeat elements within 2 kb of the backsplice junction sites are marked by colored arrows, where arrow direction indicates the orientation of the repeat element. Base pairing between complementary sequences

of repeat elements is shown by stacked lines. Trans-splicing events between splice sites of two genes are shown by broken lines. (A) To form C2A-2B-SERF1A/B(E2), the 5′ss of *SMN* exon 2B was trans-spliced to the 3′ss of *SERF1A/B* exon 2, while 5′ss of *SERF1A/B* exon 2 was trans-spliced to 3′ss of *SMN* exon 2A. Exons 2B of *SERF1A* and *SERF1B* genes are identical, here we labeled as SERF1A/B (E2). (B) In order to generated C2A-2B-ERBIN(E2-E3), the 5′ss of *SMN* exon 2B was trans-spliced to the 3′ss of *ERBIN* exon 2, while the 5′ss of *ERBIN* exon 3 was trans-spliced to the 3′ss of *SMN* exon 2A.

Supplementary Figure S11. Secondary structure of intron 5 through intron 8A supporting formation of C6-7-8A. Secondary structure of the RNA region from intron 5 to intron 8A is predicted by mfold. 5′ss of exon 8A and 3′ss of exon 6 are brought together by base pairing between intron 5 and exons 6, 7 and 8A and in the context of double-stranded RNA formed by AluSq. The sequence of *SMN* exons 6, 7, 8A and adjacent introns 8 and 8A are given. Exonic sequences are shown in colored uppercase letters and are boxed. Introns are shown in lowercase letters or as lines. AluSq and FLAM C sequences are highlighted. Red arrows indicate direction of Alu insertion. Numbering of nucleotides starts from the beginning of exon 6. The 5′ and 3′ splice sites are shown with turquoise colored arrows.

Supplementary Figure S12. RNase protection assays support formation of backsplicing junctions. (A) Detection of linear *SMN* mRNA and survey of all major backsplicing junctions. Above each gel panel, schematic of each RNA detected and probe annealing locations are shown. Sample treatments are indicated at the top of each gel. Digested band identities are shown at the right of each gel. For undigested probes (first lane of each gel panel), samples were diluted 1:200 before loading. (B) Treatment of each detected isoform by RNase R. Labeling is the same as in (A). For undigested probes (first lane of each gel panel), samples were diluted 1:1000 before loading. For mock-treated HeLa samples (third lane of each gel panel), samples were diluted 1:5 before loading.

Supplementary Figure S13. Mouse *Smn* contains numerous B elements. A scale depiction of the mouse *Smn* gene and surrounding intergenic sequences is given. Exons are represented by colored boxes and outlined in red. Repeat sequences as identified by Repeatmasker are depicted by colored arrows. Arrow direction indicates the orientation of the repeat sequence.

Supplementary Figure S14. Exon variants of mouse *Smn* identified in circRNAs. Each square contains the sequence of one exon with exon name given on the top left. The locations where the variants start or end are marked with arrows. Exon variants are labeled in black, whereas, circRNAs bearing the variants are in blue within square brackets. Black dots under the sequences mark 10-nt length. Nomenclature of exon variant and circRNAs is same as in Figure 6.

Supplementary Figure S15. Secondary structure of mouse *Smn* intron 1 through intron 4 supporting formation of mC2A-2B-3-4. Secondary structure of the RNA region from intron 1 to intron 4 is predicted by mfold. 5′ss of exon 4 and 3′ss of exon 2A are brought together by base pairing between intron 1 and exons 2A, 2B, 3 and 4. The sequence of mouse *Smn* exons 2A, 2B, 3, 4 and adjacent introns 1 and 4 are given. Exonic sequences are shown in colored uppercase letters and are boxed. Intronic sequences are shown in lowercase letters or as lines. Numbering

of nucleotides starts from the beginning of exon 2A. The 5′ and 3′ splice sites are shown with colored arrows.

Supplementary Figure S16. Linear mouse *Smn* transcripts with a novel exon 6B. (A) Alignment of mouse *Smn* intron 6 and human *SMN1* intron 6 region containing exon 6B. Numbering starts from the beginning of exon 6B. Black arrows indicate the 5′ss and the 3′ss and a red arrow indicates the position and direction of ID4. Sequences were aligned by ClustalW algorithm using MacVector software. (B) Alignment of amino acids sequences encoded by mouse *Smn* exon 6B and human *SMN1* exon 6B. (C) Annealing positions of primers used for amplification of mouse *Smn* exon 6B-containing transcripts are shown. Transcripts identities are indicated on the right and left of the gel.

Supplementary Figure S17. Potential mechanism of formation of an intron-containing mouse *Smn* transcript. (A) Formation of an intron-containing mouse *Smn* transcript, mCI4(756)-5-6-I6(237). Left panel: Divergent amplification of mouse *Smn* exon 5 (Figure 6F). The identity of circRNAs is indicated on the right side of the gel. Right panel: A diagrammatic representation of mCI4(756)-5-6-I6(237) (boxed in green) and flanking exons. The splicing event during which the 5′ss of exon 4 is brought to the vicinity of the branch point in intron 6 is indicated by a broken arrow. Exons are shown as colored shapes, introns as lines. Relevant intronic sequences are presented using lowercase letters. The identical sequences within intron 4 and 6 are boxed. Blue color letters indicate that the sequence is located within intron 4, letters in red indicate that the sequence is located within intron 6. Primers used for RT and PCR are shown as arrows. The location of the splice sites and the branch point is given as well. Abbreviations: BP, branch point. (B) Potential mechanism of RT slippage during cDNA generation. Lariat intermediate is formed during pre-mRNA splicing (step 1). RT begins to generate cDNA starting from the RT primer site in exon 5 (step 2). A potential RNA structure may bring the identical sequences shown as box 1 and 2 in close proximity facilitating RTase to jump from “box 1” to “box 2” (step 3). As a result, cDNA molecules would bear only one “copy” of the identical sequence. White boxes 1 and 2 correspond to the identical sequences (CUGGUCU) within intron 4 and 6, respectively. Intron 4 is shown as a blue line and intron 6 as a red line. Exons are shown as colored shapes. RT is shown as a pink oval. Black box indicates RT primer that anneals in exon 5.

Supplementary Figure S18. DRB treatment impacts *SMN* transcripts at different time points. (A) Left panel: a schematic presentation of experimental time frame. Right panel: An overview of numbered treatments. Each number indicates a different treatment. (B) Left panel: MESDA depicting splicing pattern of the linear *SMN* pre-mRNA after DRB treatment. Sample treatment and time are indicated at the top of the ethidium bromide stained gel. “Δ” indicates skipping of the indicated exon(s). Right panel: quantification of relative band intensities. Sample treatment is indicated at the bottom of the graph.

Supplementary Figure S19. Effect of Anti-N1 on the expression levels of circRNAs in HeLa and GM03813 cells. (A) Diagrammatic representation of ASOs used to affect splicing of *SMN*. Location and base pair of ASOs in ISS-N1 region within *SMN* intron 7. ISS-N1 region is highlighted in a color box. Untr: untransfected. (B, E) Top panel: MESDA depicting splicing of linear *SMN* pre-mRNA after ASO transfection. Sample treatment is indicated at the top of the

gel. Each treatment was performed in duplicate. "Δ" indicates skipping of the indicated exon(s). Bottom panel: quantification of relative band intensities. (C, F) Ethidium bromide stained gel of divergent RT-PCR using primers annealing to exon 3, which produces mostly type 1 circRNAs, after ASO transfection. Sample treatments are indicated at the top. Band identities are labeled on the right side of gel. (D, G) Ethidium bromide stained gel of divergent RT-PCR using primers annealing to exon 6, which produces mostly type 3 circRNAs, after ASO transfection. Sample treatments are indicated at the top. Band identities are labeled on the right side of gel.

Supplementary Figure S1

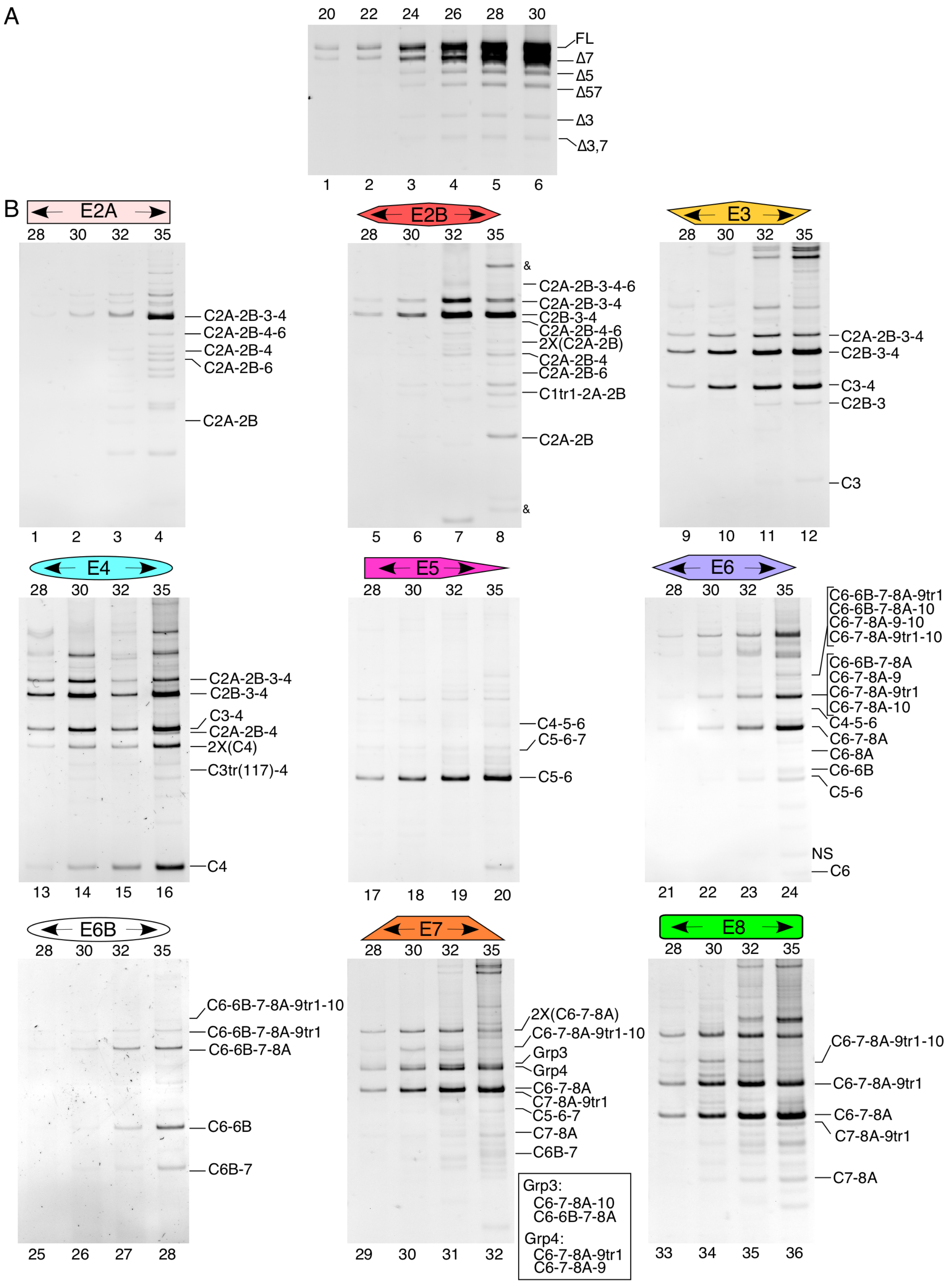

Supplementary Figure S2

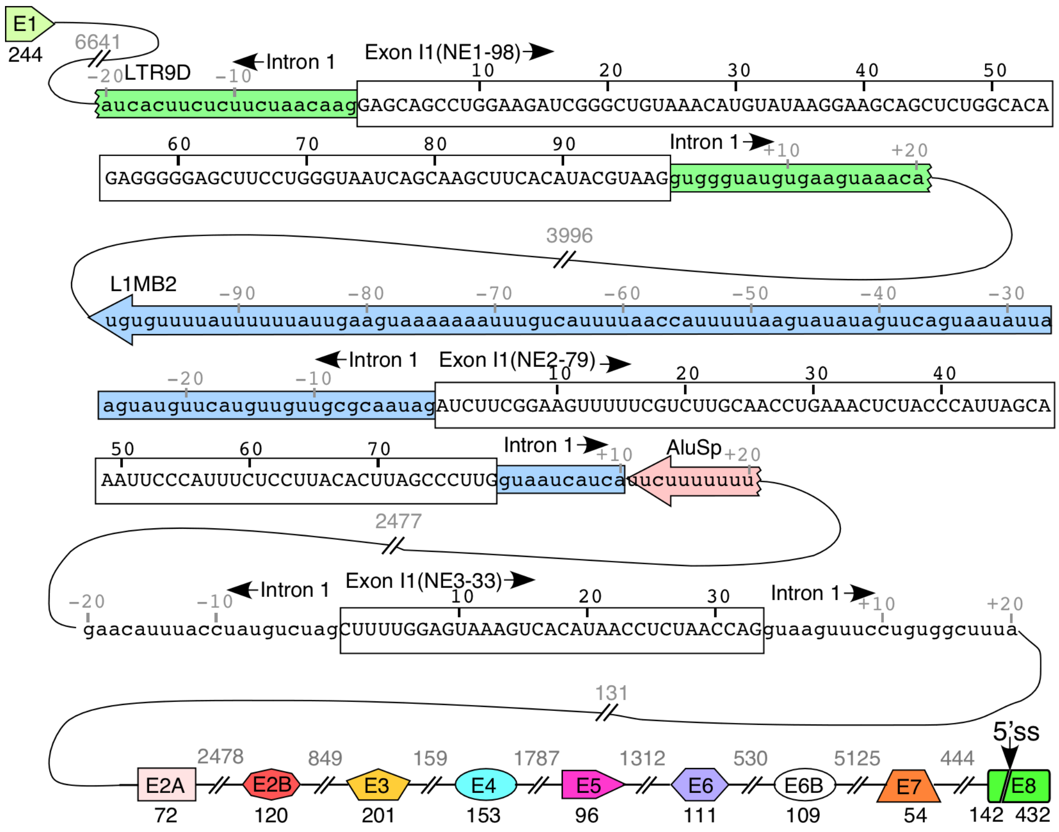

Supplementary Figure S3

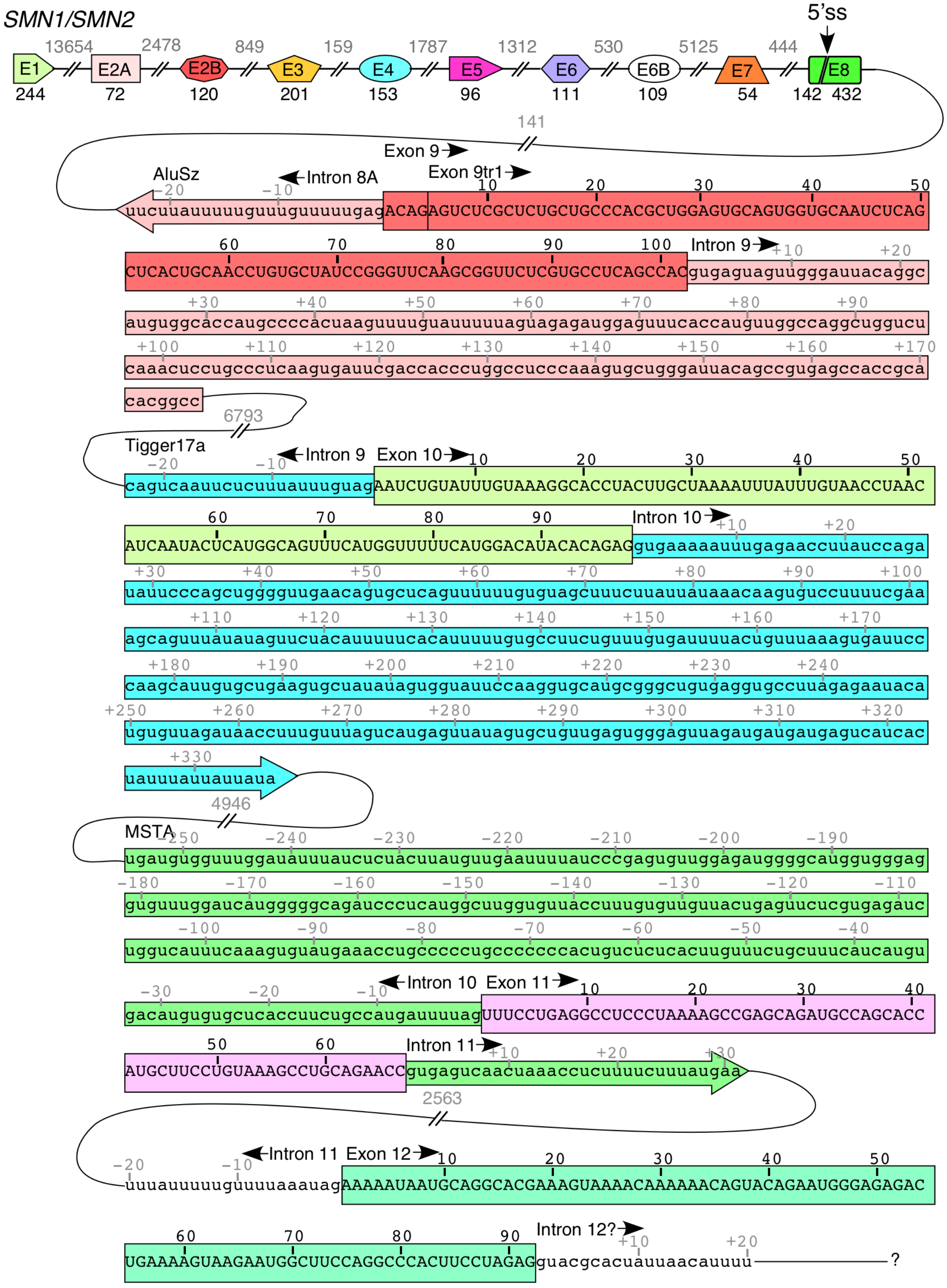

Supplementary Figure S4

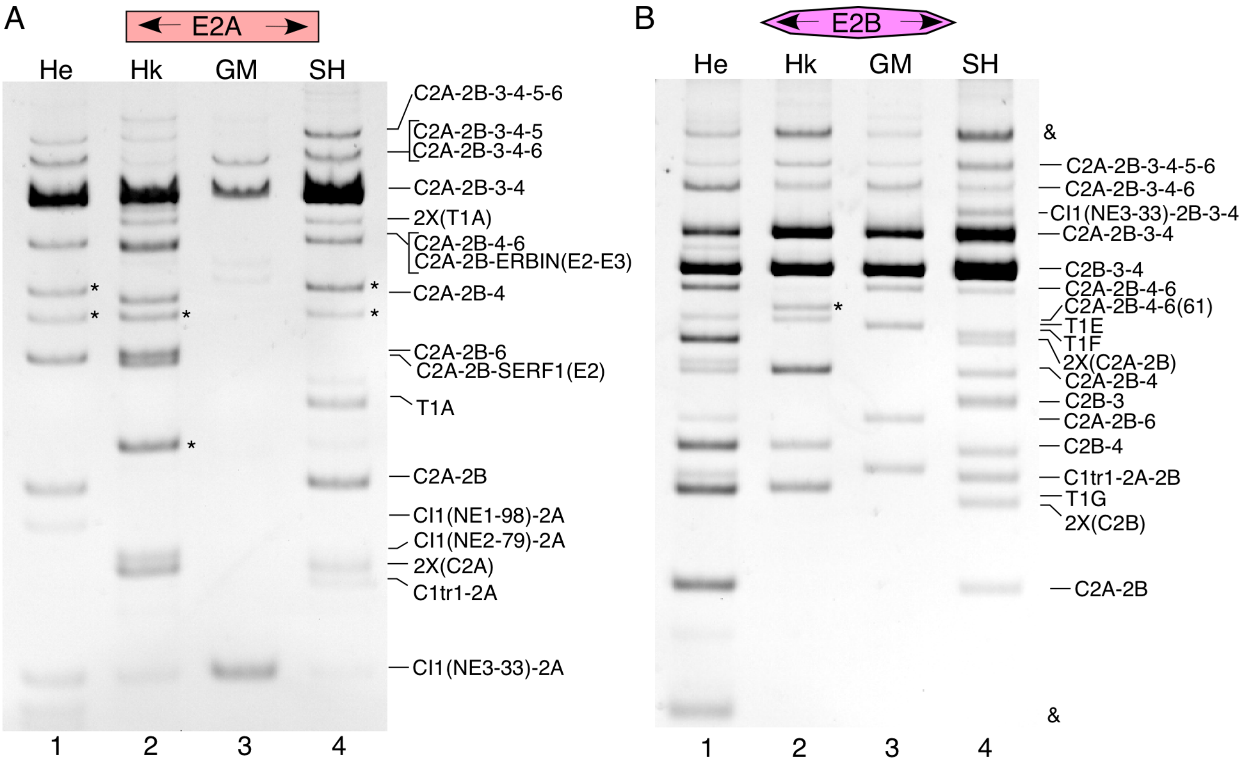

Supplementary Figure S5

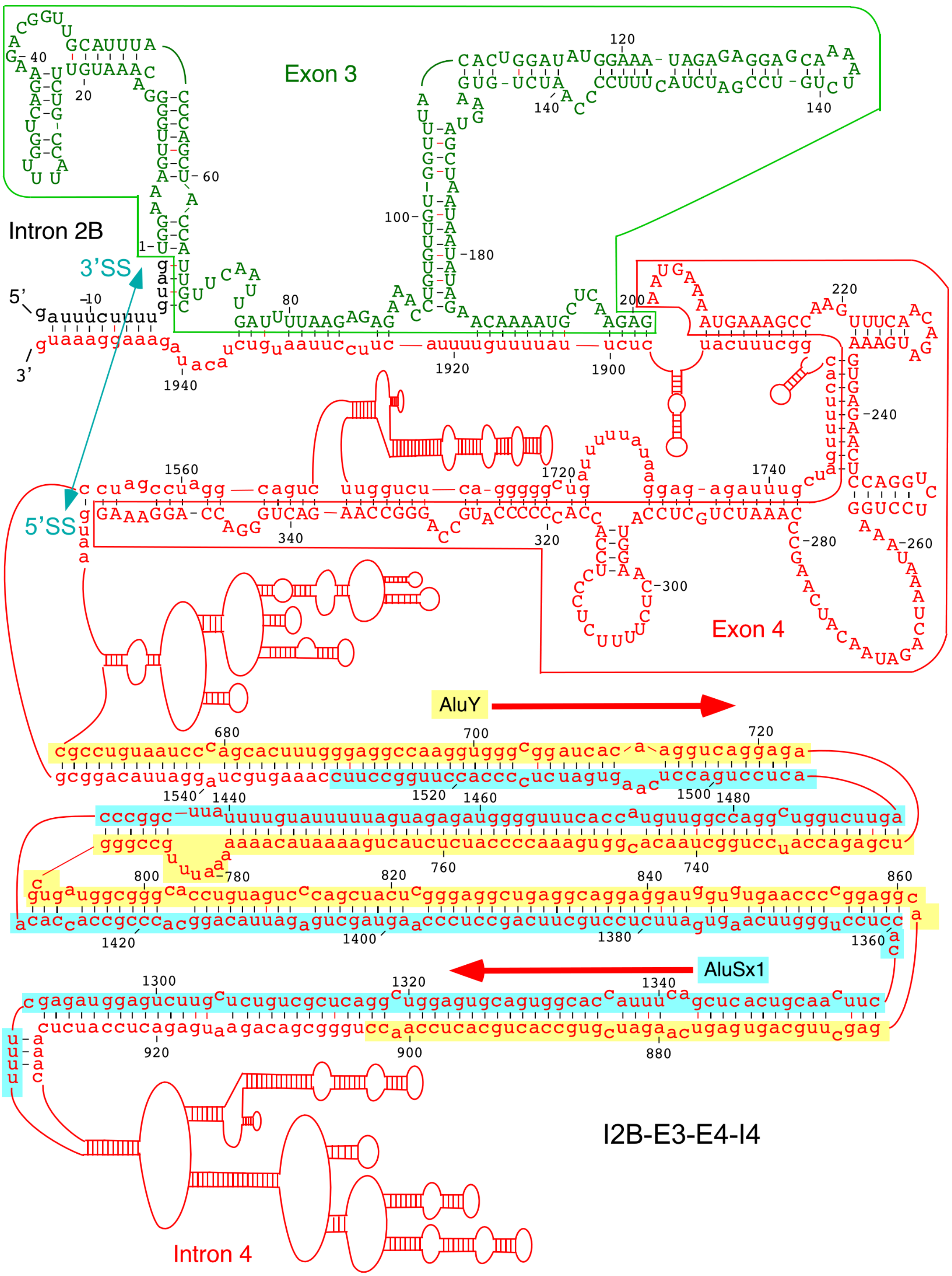

Supplementary Figure S6

I2A-E2B-E3-E4-I4

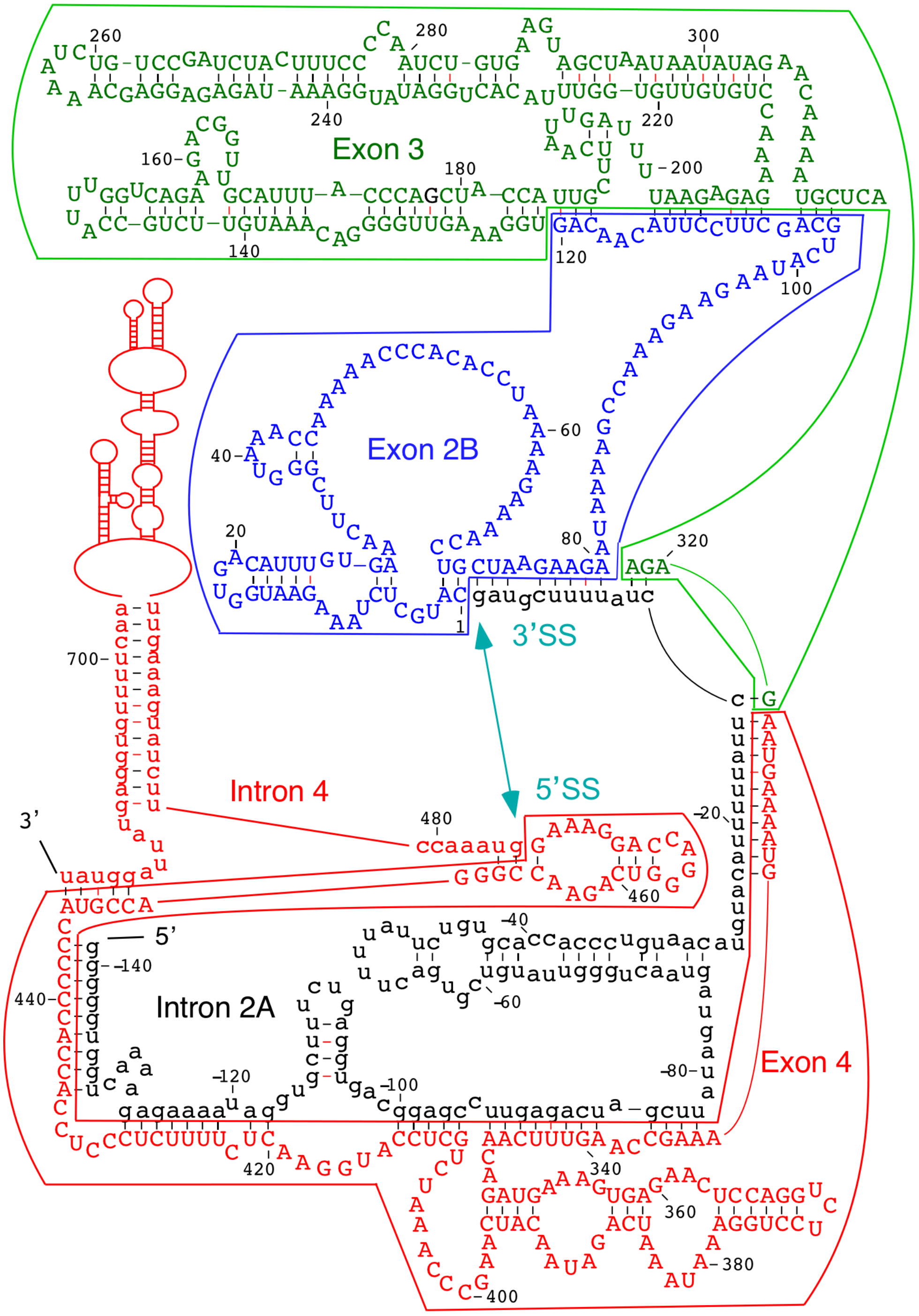

Supplementary Figure S7

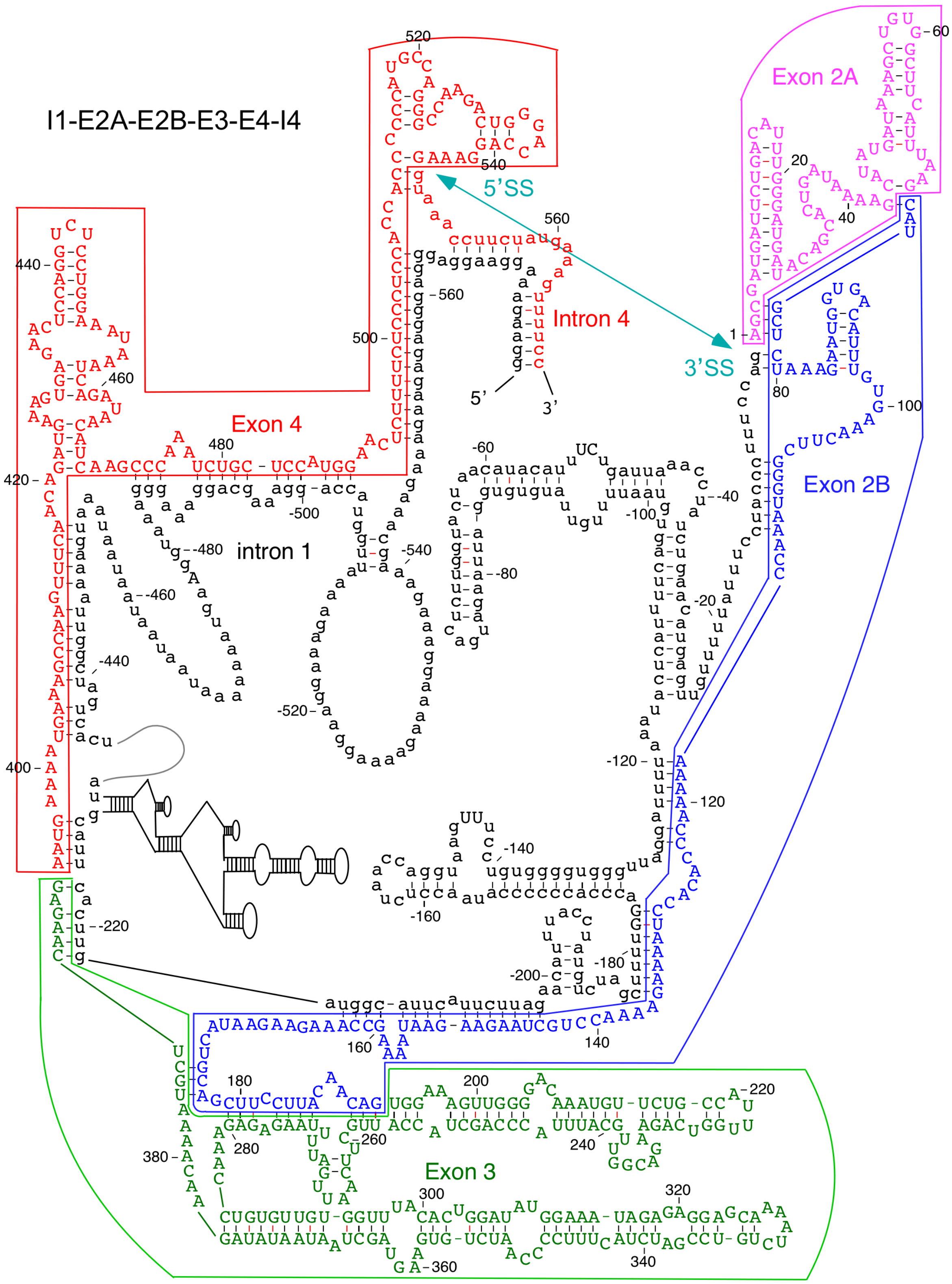

Supplementary Figure S8

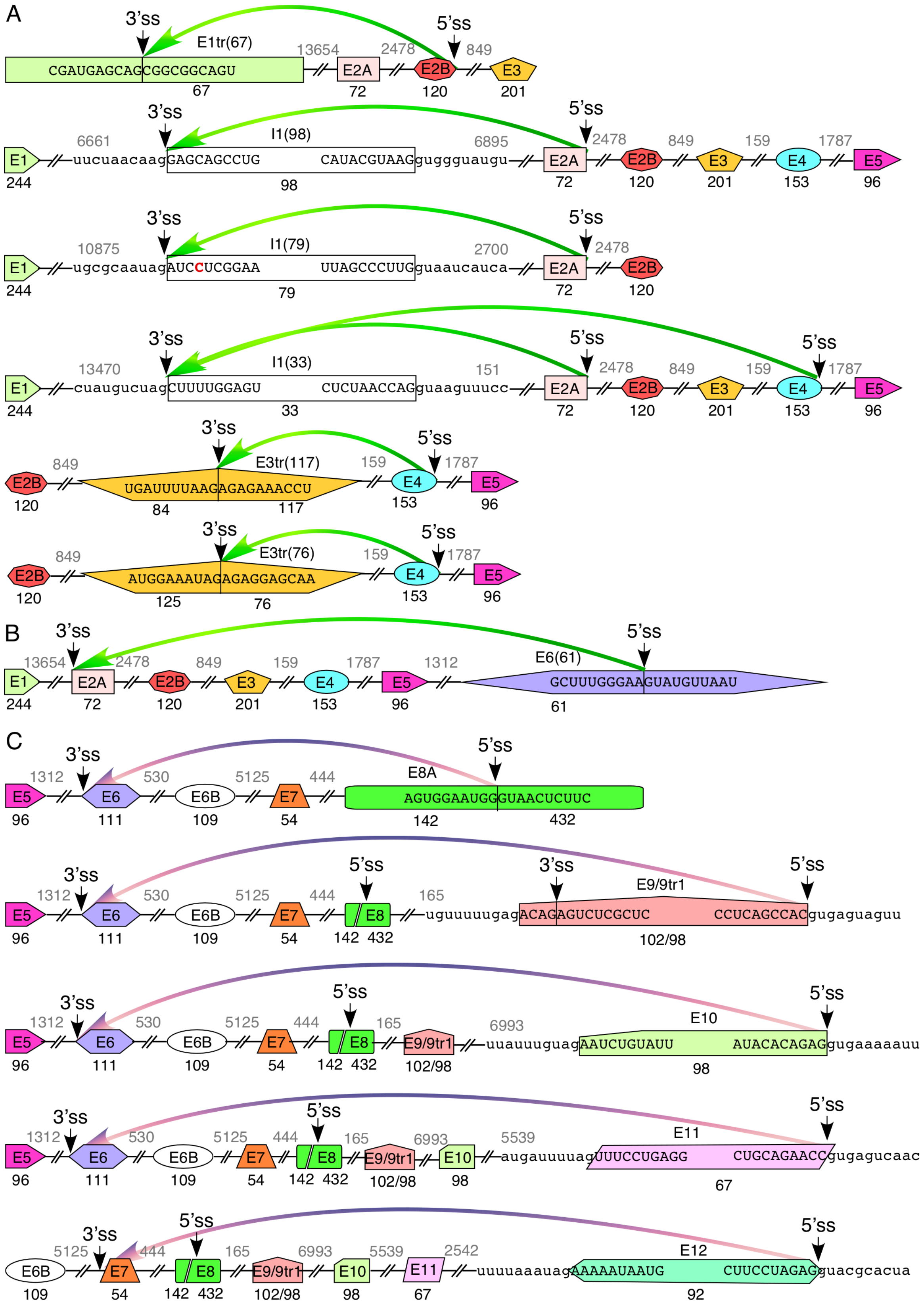

Supplementary Figure S9

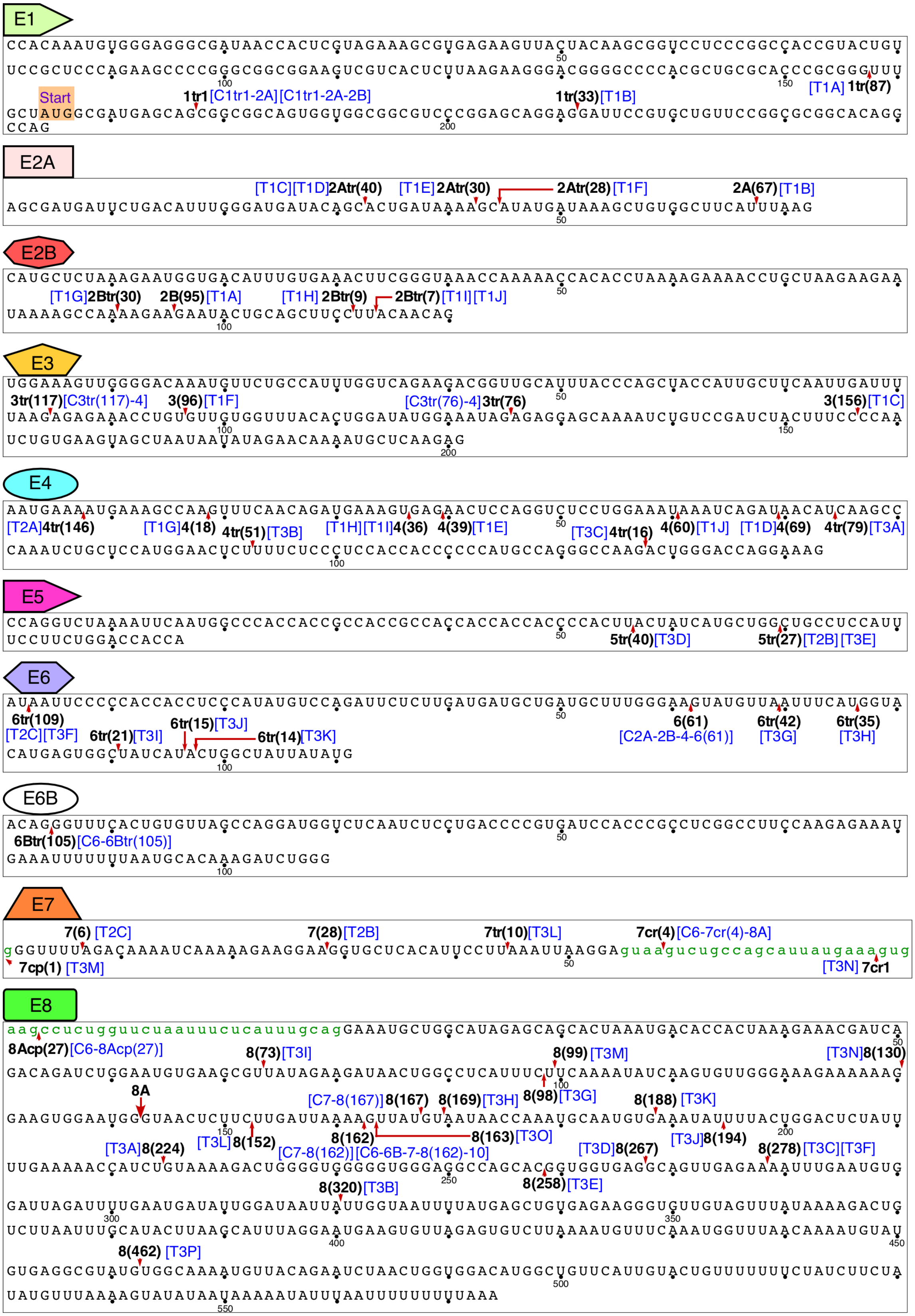

Supplementary Figure S10

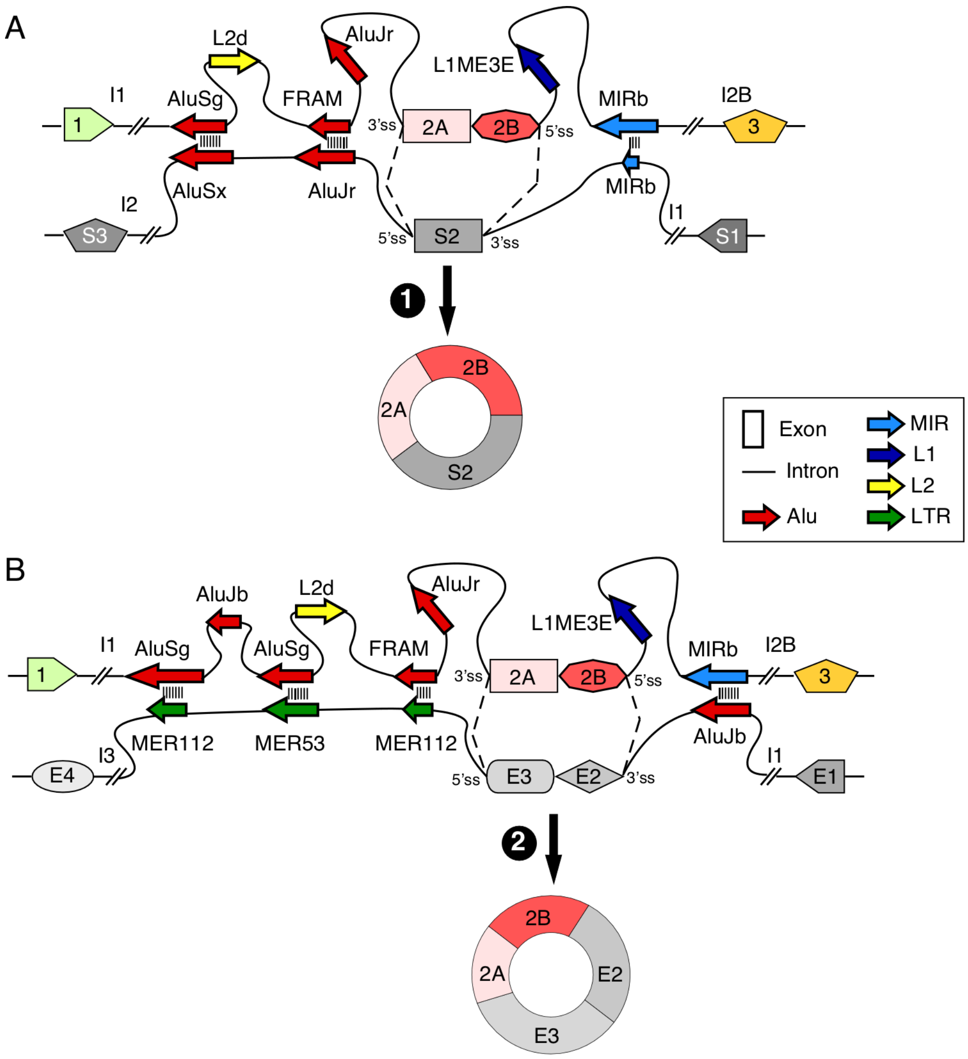

Supplementary Figure S11

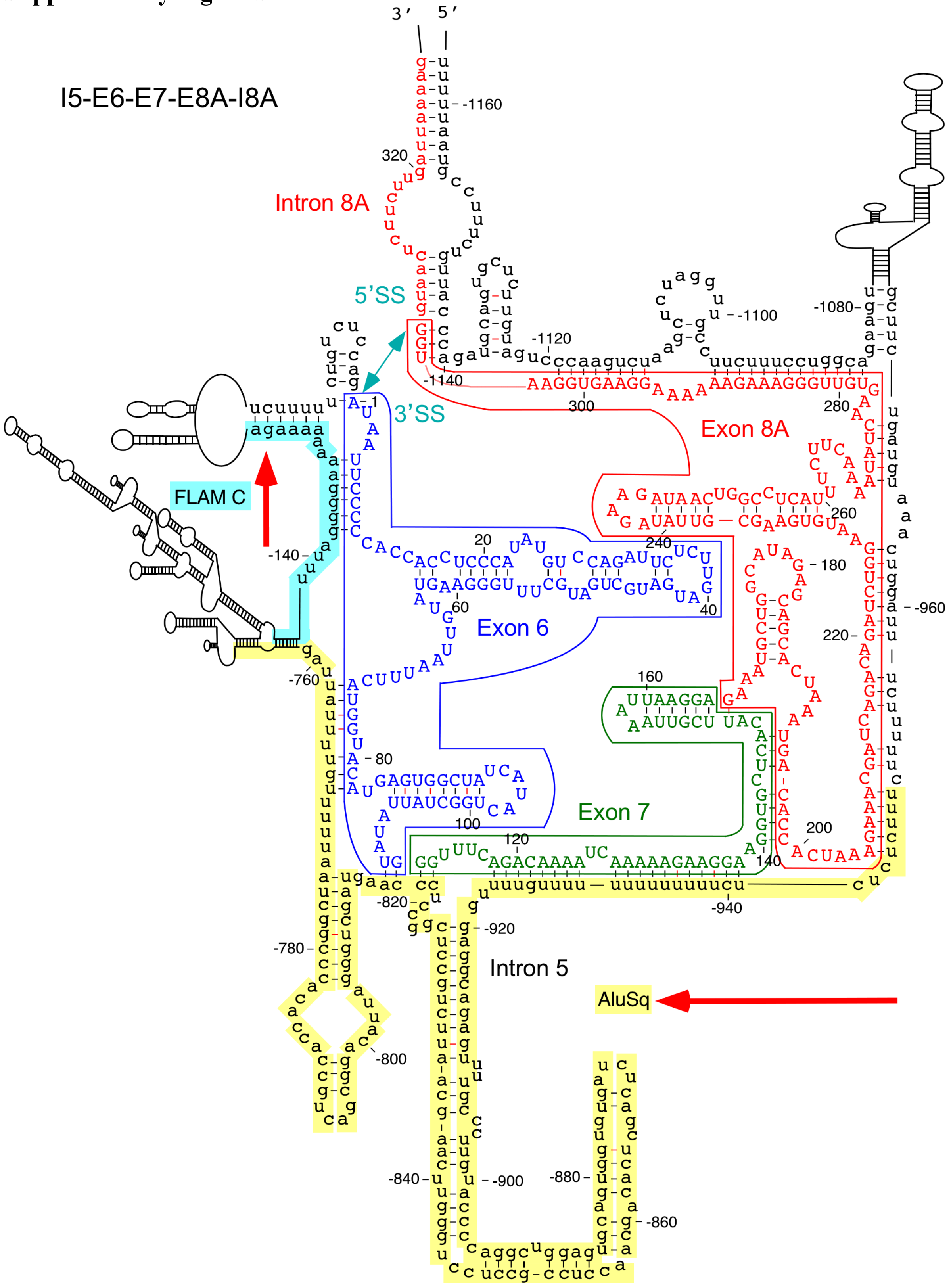

Supplementary Figure S12

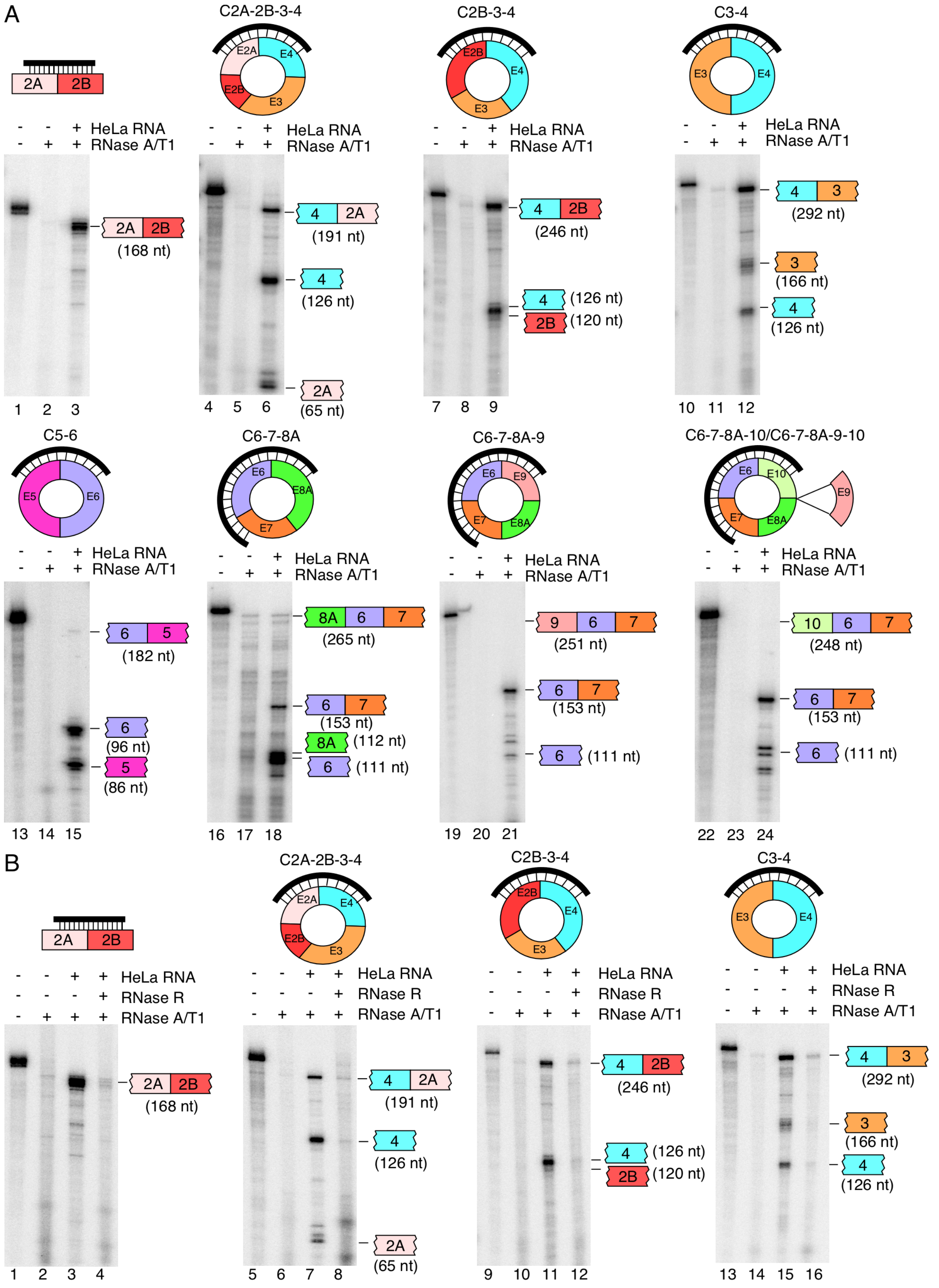

Supplementary Figure S13

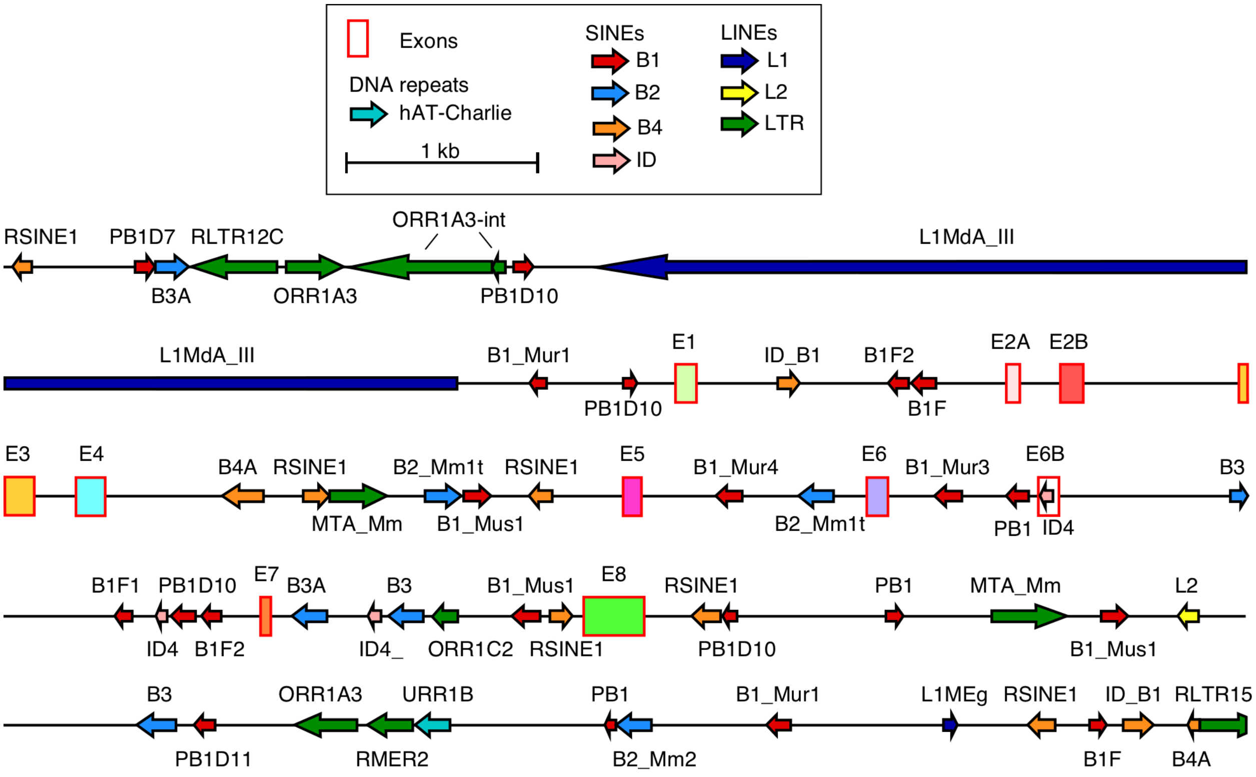

Supplementary Figure S14

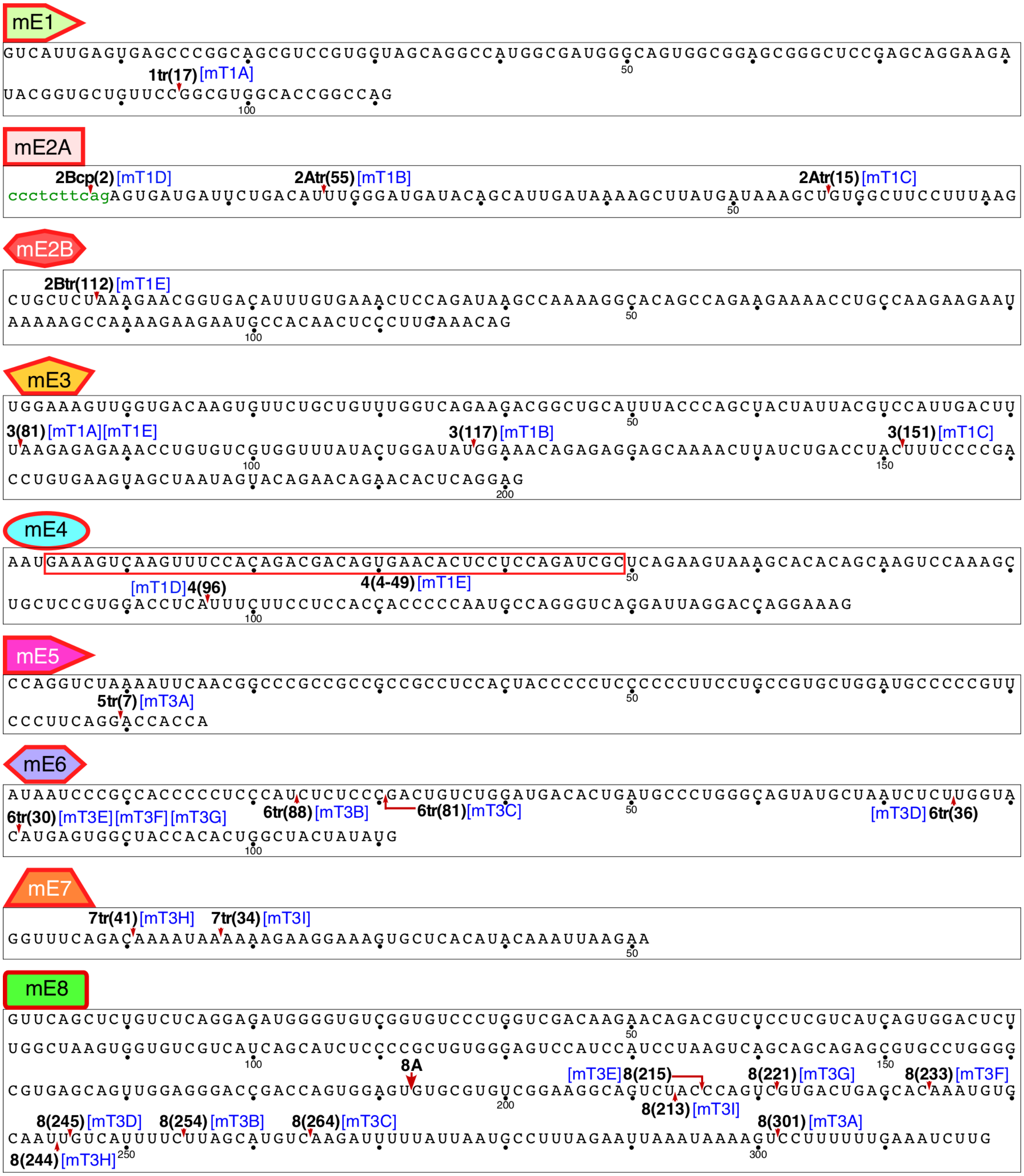

Supplementary Figure S15

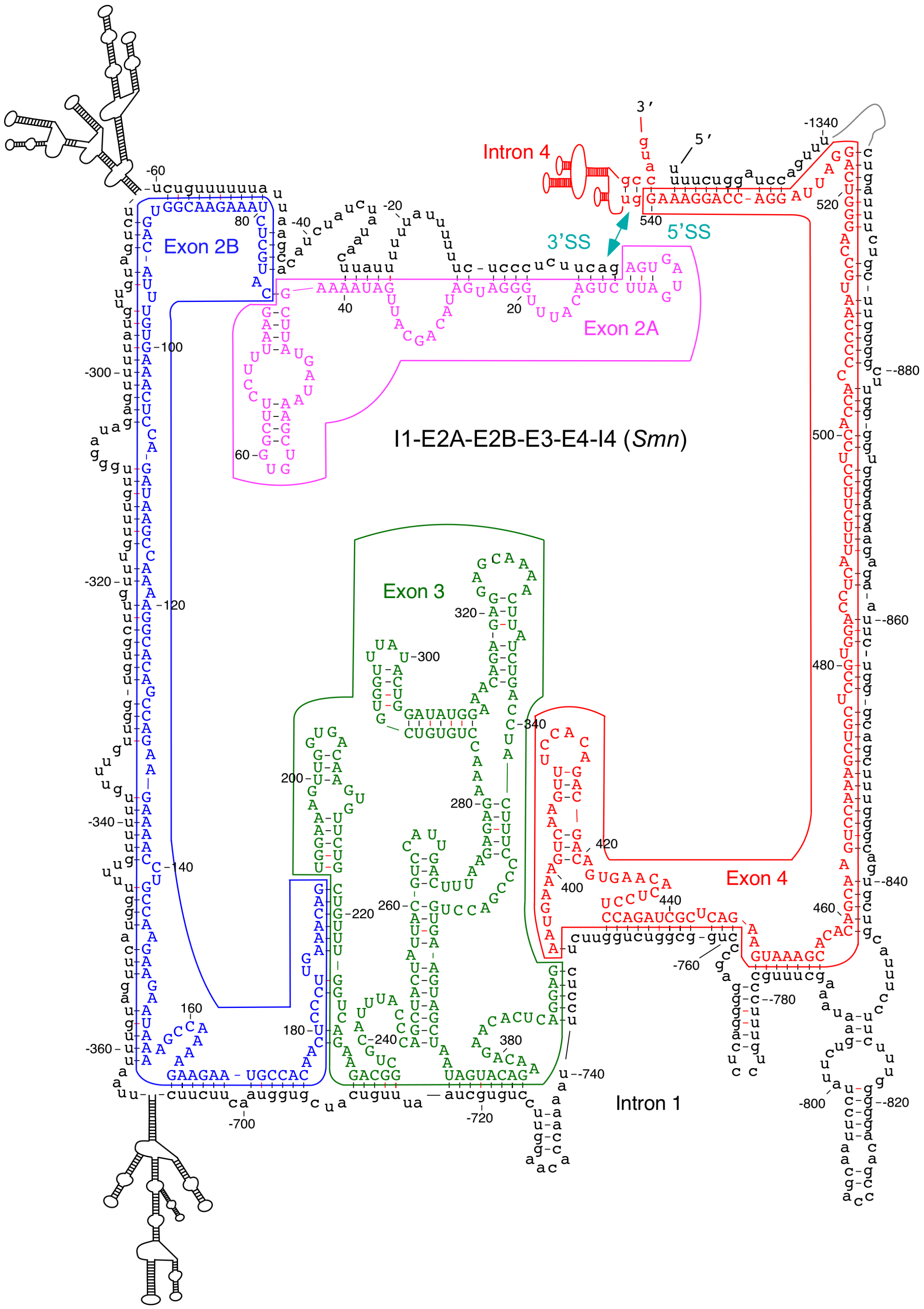

Supplementary Figure S16

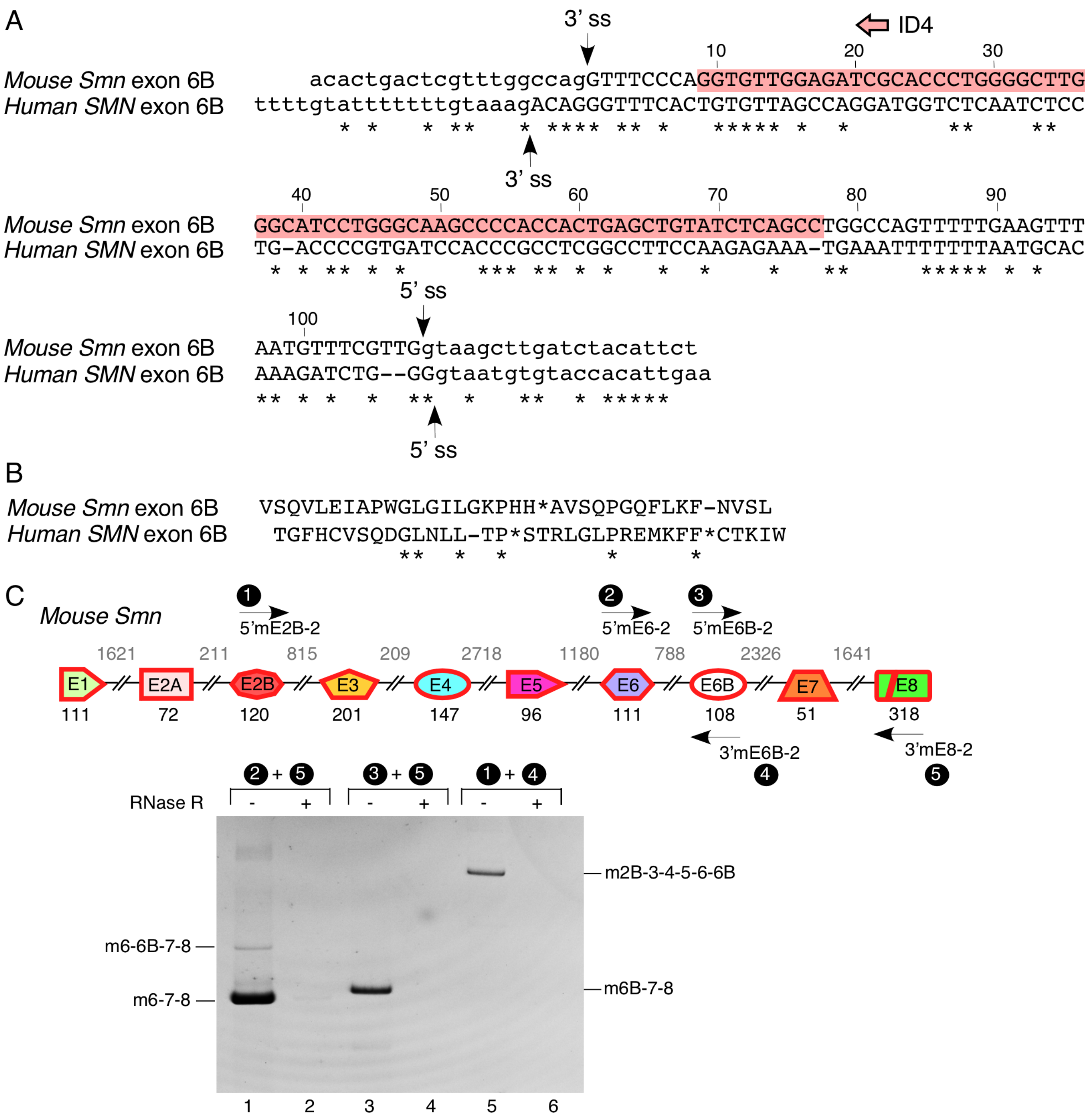

Supplementary Figure S17

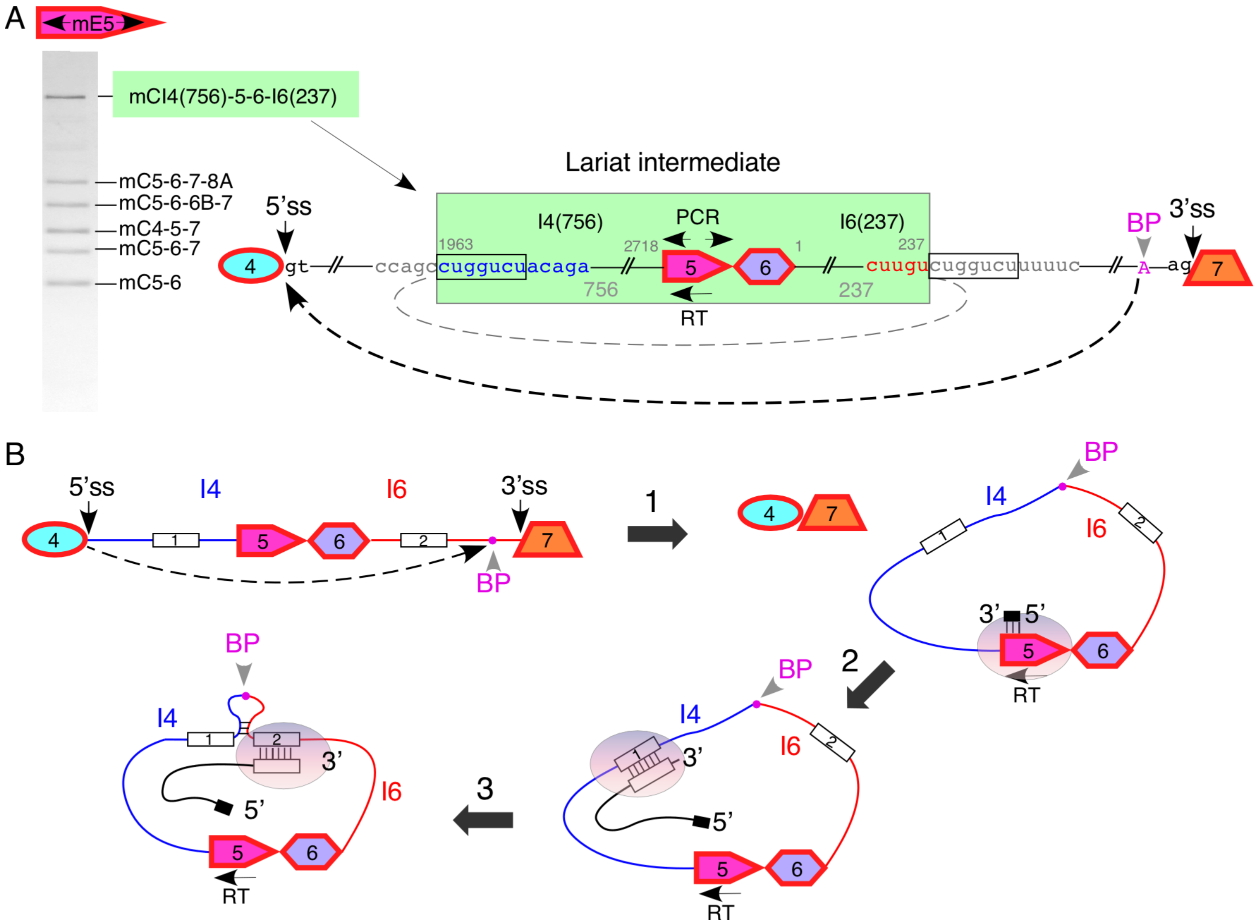

Supplementary Figure S18

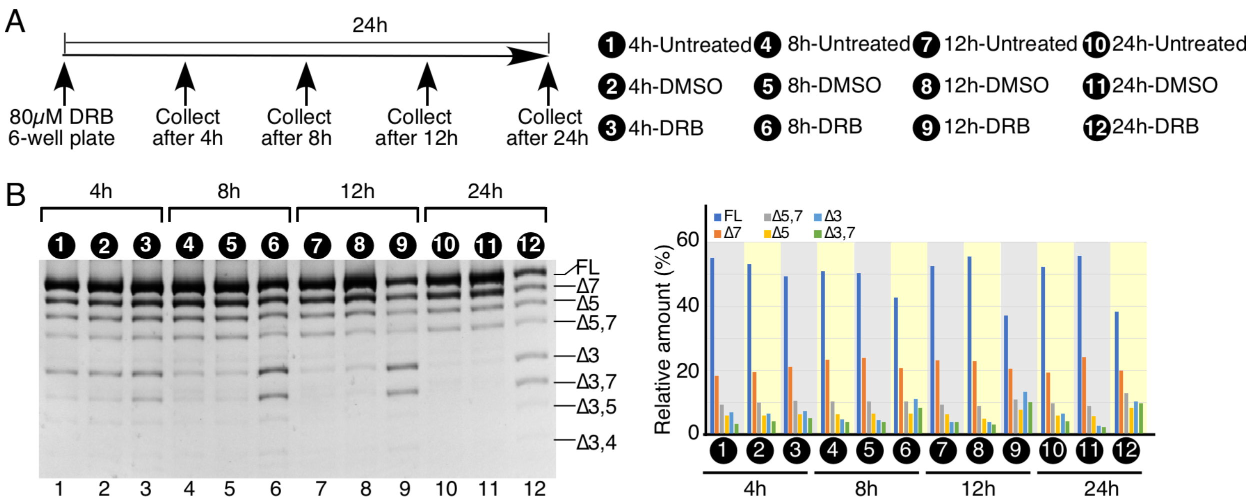

Supplementary Figure S19

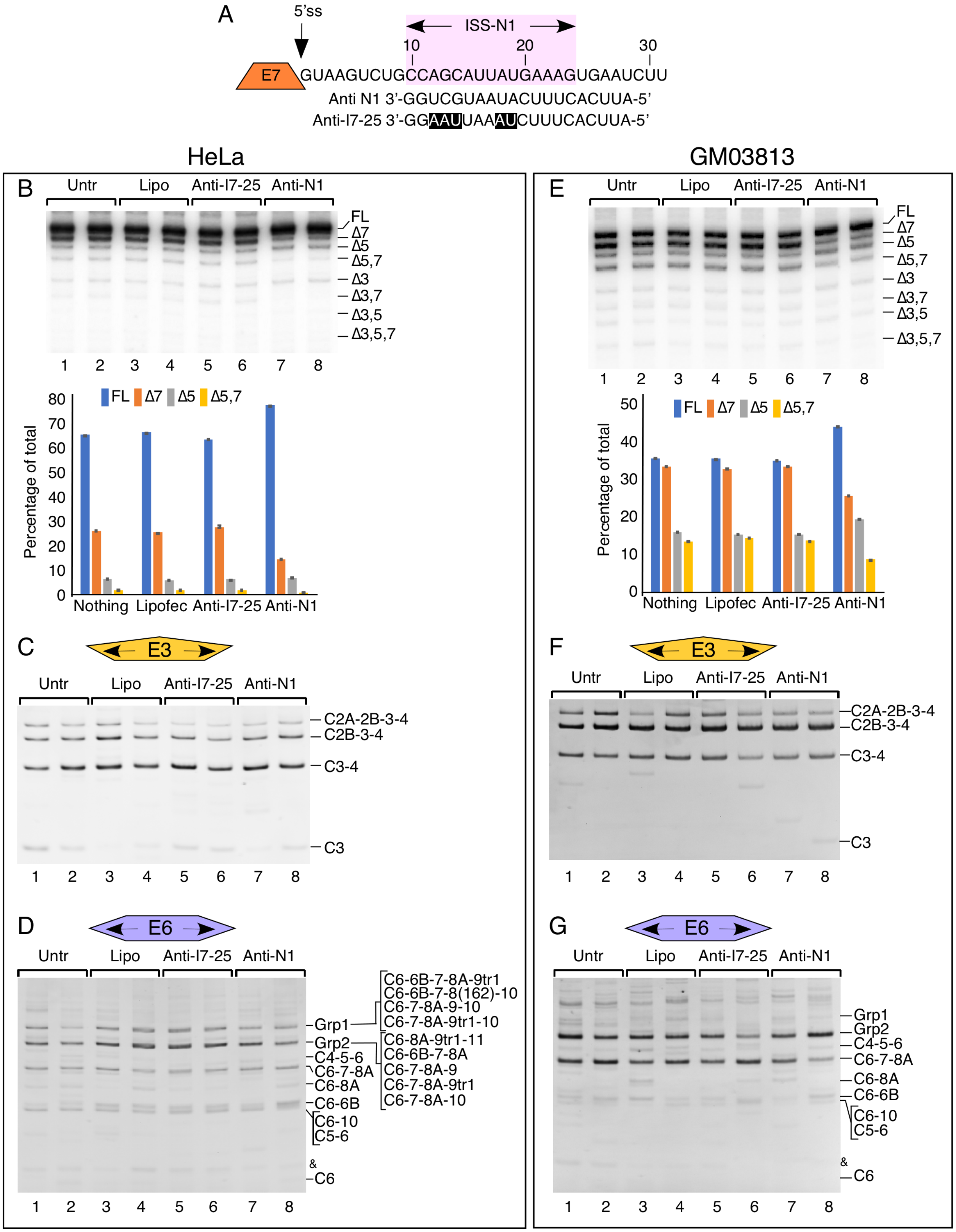

Supplementary Tables

Table S1. List of primers used for PCR and qPCR

	Primer name	Species	Target exon	Primer sequence		
1	5'E2A-End	Human	2A	5'-	GCATATGATAAAGCTGTGGGCTTC	-3'
2	3'E2A-Start	Human	2A	5'-	CCCAAATGTCAGAATCATCGC	-3'
3	3'E2A-RT	Human	2A	5'-	TGCTTTTATCAGTGCTGTATCAT	-3'
4	5'E2B-End	Human	2B	5'-	GAATACTGCAGCTTCCTTACAAC	-3'
5	3'E2B-Start	Human	2B	5'-	CAAATGTCACCATTCTTTAGAGC	-3'
6	3'E2B-RT	Human	2B	5'-	GGCTTTTATTCTTCTTAGCAGG	-3'
7	5'E3-End	Human	3	5'-	CTTTCCCCAATCTGTGAAGTAGCTA	-3'
8	3'E3-Start	Human	3	5'-	CAACCGTCTTCTGACCAAATGG	-3'
9	3'E3-RT	Human	3	5'-	TCCATATCCAGTGTAAACCACAAC	-3'
10	5'E4-End	Human	4	5'-	GGCCAAGACTGGGACCAGG	-3'
11	3'E4-Start	Human	4	5'-	TCACTTTCATCTGTTGAAACTTGG	-3'
12	3'E4-RT	Human	4	5'-	GTTATCTGATTTATTTCCAGGAGACC	-3'
13	5'E5-End	Human	5	5'-	CTGCCTCCATTTCCTTCTGGAC	-3'
14	3'E5-Start	Human	5	5'-	GTGGTGGGCCATTGAATTTTAGAC	-3'
15	3'E5-RT	Human	5	5'-	CATGATAGTAAGTGGGGTGGTG	-3'
16	5'E6-End	Human	6	5'-	TCATGGTACATGAGTGGCTATCA	-3'
17	3'E6-Start	Human	6	5'-	CATATGGGAGGTGGTGGG	-3'
18	3'E6-RT	Human	6	5'-	ATTAACATACTTCCCAAAGCATCAG	-3'
19	5'E6B-End	Human	6B	5'-	ATGAAATTTTTTTAATGCACAAAGATCTG	-3'
20	3'E6B-Start	Human	6B	5'-	GGCTAACACAGTGAAACCCTG	-3'
21	3'E6B-RT	Human	6B	5'-	GATCACGGGGTCAGGAGATTG	-3'
22	5'E7-End	Human	7	5'-	GTGCTCACATTCCTTAAATTAAGG	-3'
23	3'E7-Start	Human	7	5'-	CTTCTTTTTGATTTTGTCTGAAACC	-3'
24	3'E7-RT	Human	7	5'-	GCACCTTCCTTCTTTTTGATTTTG	-3'
25	5'E8A-End	Human	8	5'-	GTTGGGAAAGAAAAAAGGAAGTGG	-3'
26	3'E8-Start	Human	8	5'-	GGTGTCATTTAGTGCTGCTCTAT	-3'
27	3'E8-RT	Human	8	5'-	CTTCACATTCCAGATCTGTCTG	-3'
28	3'E10-Start	Human	10	5'-	TAGCAAGTAGGTGCCTTTACAA	-3'
29	5'E11-End	Human	11	5'-	CCTGTAAAGCCTGCAGAACC	-3'
30	3'E11-Start	Human	11	5'-	TCGGCTTTTAGGGAGGCC	-3'
31	3'E11-RT	Human	11	5'-	AAGCATGGTGCTGGCATC	-3'
32	5'E12-end	Human	12	5'-	AGTAAGAATGGCTTCCAGGC	-3'
33	3'E12-Start	Human	12	5'-	TGTTTTACTTTCGTGCCTGC	-3'
34	3'E12-RT	Human	12	5'-	CAGTCTCTCCCATTCTGTACTG	-3'
35	5'E1	Human	1	5'-	CGCGGGTTTGCTATGGCGAT	-3'
36	3'E8-25	Human	8	5'-	TAGTGCTGCTCTATGCCAGCATTTC	-3'
37	5'mE2A	Mouse	2A	5'-	CTTATGATAAAGCTGTGGCTTCCT	-3'
38	3'mE2A	Mouse	2A	5'-	TATCATCCCAAATGTCAGAATCATCA	-3'
39	3'mE2A-RT	Mouse	2A	5'-	CTTTATCATAAGCTTTTATCAATGC	-3'
40	5'mE2B	Mouse	2B	5'-	AAAGCCAAAAGAAGAATGCCACAA	-3'
41	3'mE2B	Mouse	2B	5'-	CTGGAGTTTCACAAATGTCACCG	-3'
42	3'mE2B-RT	Mouse	2B	5'-	CTTCTTGGCAGGTTTTCTTCTGG	-3'
43	5'mE3	Mouse	3	5'-	ACAGAGAGGAGCAAAACTTATCTGAC	-3'
44	3'mE3	Mouse	3	5'-	TCTCTCTTAAAGTCAATGGACGTAATAG	-3'
45	3'mE3-RT	Mouse	3	5'-	TCCTGAGTGTTCTGTTCTGTACTA	-3'
46	5'mE4	Mouse	4	5'-	CCAATGCCAGGGTCAGGATTA	-3'
47	3'mE4	Mouse	4	5'-	TTTACTTCTGAGCGATCTGGAGG	-3'

48	3'mE4-RT	Mouse	4	5'-	AGAAATGAGGTCCACGGAGC	-3'
49	5'mE5	Mouse	5	5'-	CCGTTCCCTTCAGGACCAC	-3'
50	3'mE5	Mouse	5	5'-	CGGGCCGTTGAATTTTAGACCT	-3'
51	3'mE5-RT	Mouse	5	5'-	CATCCAGCACGGCAGGAAGG	-3'
52	5'mE6	Mouse	6	5'-	ATCTCTTGGTACATGAGTGGCTACCAC	-3'
53	3'mE6	Mouse	6	5'-	GTGTCATCCAGACAGTCGGGAGAG	-3'
54	3'mE6-RT	Mouse	6	5'-	CAGGGCATCAGTGTCATCCAG	-3'
55	5'mE6B	Mouse	6B	5'-	CTGTATCTCAGCCTGGCCAG	-3'
56	3'mE6B	Mouse	6B	5'-	GTGCGATCTCCAACACCTG	-3'
57	3'mE6B-RT	Mouse	6B	5'-	GAGATACAGCTCAGTGGTGGG	-3'
58	5'mE7	Mouse	7	5'-	GGAAAGTGCTCACATACAAATTAAGA	-3'
59	3'mE7	Mouse	7	5'-	TTCTTTTTTATTTTGTCTGAAACC	-3'
60	3'mE7-RT	Mouse	7	5'-	CTTAATTTGTATGTGAGCACTTTCCTTC	-3'
61	5'mE8	Mouse	8	5'-	TCCATCCTAAGTCAGCAGCAG	-3'
62	3'mE8	Mouse	8	5'-	CCCATCTCCTGAGACAGAGC	-3'
63	3'mE8-RT	Mouse	8	5'-	GTTCTTGTCGACCAGGGACACC	-3'
64	5'mE2B-2	Mouse	2B	5'-	GAAGAATGCCACAACTCCCTTGA	-3'
65	5'mE6-2	Mouse	6	5'-	GAGTGGCTACCACACTGGCTAC	-3'
66	5'mE6B-2	Mouse	6B	5'-	GTATCTCAGCCTGGCCAGTTTTTG	-3'
67	3'mE6B-2	Mouse	6B	5'-	CAAAAACTGGCCAGGCTGAGATAC	-3'
68	3'mE8-2	Mouse	8	5'-	CACGCTCTGCTGCTGACTTAGG	-3'
69	3'JxnE1/E2A	Human	1/2A	5'-	CAGAATCATCGCTCTGGCCTGT	-3'
70	5'GAPDH	Human	8	5'-	AACAGCGACACCCACTCCTC	-3'
71	3'GAPDH	Human	8	5'-	CATACCAGGAAATGAGCTTGACAA	-3'
72	5'E8A	Human	8	5'-	CGATCAGACAGATCTGGAATGTG	-3'
73	3'Jxn E8A/E10	Human	8/10	5'-	GTGCCTTTACAAATACAGATTCCATT	-3'
74	5'Jxn E8A/E11	Human	8/11	5'-	GAAAAAAGGAAGTGGAATGGTTTCC	-3'
75	3'E11-end	Human	11	5'-	GGTTCTGCAGGCTTTACAGG	-3'
76	5'Jxn E9/E10	Human	9/10	5'-	CTCGTGCCTCAGCCACAATCT	-3'
77	3'E10-RT	Human	10	5'-	CATGAAACTGCCATGAGTATTGATGTTAGG	-3'
78	5'Jxn E2B/E6	Human	2B/6	5'-	CTGCAGCTTCCTTACAACAGATAAT	-3'
79	3'E6	Human	6	5'-	GCCAGTATGATAGCCACTCATG	-3'
80	5'18S	Human	-	5'-	GGCCCTGTAATTGGAATGAGTC	-3'
81	3'18S	Human	-	5'-	CCAAGATCCAACTACGAGCTT	-3'

Table S2. Description of linear splice variants incorporating novel *Smn* and *SMN* exons.

Exon	Gene	Cell line	GenBank accession number
mE6-6B-7-8	Mouse *Smn*	NSC34	MH378773
E6-7-8A-9tr1-11-12	Human *SMN1*	HeLa	MH500447
E6-7-8A-11-12	Human *SMN1*	HeLa	MH500448
E6-7-8A-9tr1-10	Human *SMN1*	HeLa	MH500449
E6-7-8A-10	Human *SMN1*	HeLa	MH500450

Table S3. List of circRNAs identified in this study.

#	Primer location	Name of circRNA	Cell line	GenBank Accession number
1	KE2A	CI1(NE2-79)-2A	HEK-293	MH500413
2	KE2A	C2A-2B-3-4	HEK-293	MH500408
3	KE2A	C2A-2B-4-6	HEK-293	MH500410
4	KE2A	C2A-2B-4	HEK-293	MH500411
5	KE2A	C2A-2B-6	HEK-293	MH500412
6	KE2A	C2A-2B-SERF1A/B(E2)	HEK-293	MH500526
7	KE2A	C2A-2B-ERBIN(E2-E3)	HEK-293	MH500409
8	SE2A	C1tr1-2A	SH-SY5Y	MH500414
9	SE2A	C1tr(87)-2A-2B(95)(2X)	SH-SY5Y	MH500529
10	SE2A	C1tr(87)-2A-2B(95)	SH-SY5Y	MH500530
11	SE2A	C2A-2B-3-4-5-6	SH-SY5Y	MH500514
12	SE2A	C2A-2B-3-4-5	SH-SY5Y	MH500515
13	SE2A	C2A-2B-3-4-6	SH-SY5Y	MH500516
14	HE2A	C1tr(33)-2A(67)	HeLa	MH500415
15	HE2A	CI1(NE1-98)-2A	HeLa	MH500525
16	GE2A	CI1(NE3-33)-2A	GM03813	MH500517
17	KE2B	C2A-2B-3-4-6	HEK-293	MH500416
18	KE2B	C2A-2B-3-4	HEK-293	MH500417
19	KE2B	C2B-3-4	HEK-293	MH500418
20	KE2B	C2A-2B-4-6	HEK-293	MH500419
21	KE2B	C2A-2B-4	HEK-293	MH500420
22	KE2B	C2A-2B-6	HEK-293	MH500421
23	KE2B	C2A-2B	HEK-293	MH500422
24	SE2B	CI1(NE3-33)-2A-2B-3-4	SH-SY5Y	MH500533
25	SE2B	C2A-2B-3-4-5-6	SH-SY5Y	MH500518
26	SE2B	C2A-2B-3-4	SH-SY5Y	MH500534
27	SE2B	C2Atr(40)-2B-3(156)	SH-SY5Y	MH500535
28	SE2B	C2Atr(40)-2B-4(69)	SH-SY5Y	MH500424
29	SE2B	C2B-4	SH-SY5Y	MH500522
30	SE2B	C2B(2X)	SH-SY5Y	MH500523
31	SE2B	C2B-3	SH-SY5Y	MH500425
32	HE2B	C1tr1-2A-2B	HeLa	MH500423
33	HE2B	C2Atr(28)-2B-3(96)	HeLa	MH500532
34	HE2B	C2A-2B-4-6(61)	HeLa	MH500531
35	HE2B	C2A-2B(2X)	HeLa	MH500521

36	HE2B	C2B	HeLa	MH500426
37	KE3	C2A-2B-3-4	HEK-293	MH500427
38	KE3	C2B-3-4	HEK-293	MH500428
39	KE3	C2B-3	HEK-293	MH500430
40	KE3	C2Btr(9)-3-4(36)	HEK-293	MH500431
41	KE3	C3-4	HEK-293	MH500429
42	KE3	C3	HEK-293	MH500432
43	SE3	C2Btr(7)-3-4(60)	SH-SY5Y	MH500433
44	SE3	C2Btr(30)-3-4(18)	SH-SY5Y	MH500434
45	SE3	C2Btr(7)-3-4(36)	SH-SY5Y	MH500435
46	KE4	C2A-2B-3-4	HEK-293	MH500436
47	KE4	C2B-3-4	HEK-293	MH500437
48	KE4	C3-4	HEK-293	MH500438
49	KE4	2xC4	HEK-293	MH500439
50	KE4	C3tr(117)-4	HEK-293	MH500440
51	KE4	C4	HEK-293	MH500441
52	HE4	C2A-2B-4	HeLa	MH500442
53	GE4	C3tr(76)-4	GM03813	MH500443
54	KE5	C4-5-6	HEK-293	MH500444
55	KE5	C5-6-7	HEK-293	MH500446
56	KE5	C5-6	HEK-293	MH500445
57	SE5	C2A-2B-4-5-6	SH-SY5Y	MH500519
58	SE5	C3-4-5	SH-SY5Y	MH500451
59	SE5	C4tr(15)-5-6-8(279)	SH-SY5Y	MH500537
60	SE5	C4tr(146)-5	SH-SY5Y	MH500452
61	SE5	C5-6(2X)	SH-SY5Y	MH500536
62	GE5	C4tr(51)-5-6-7-8(320)	GM03813	MH500453
63	GE5	C4tr(79)-5-6-8(224)	GM03813	MH500454
64	KE6	C6-6B	HEK-293	MH500462
65	KE6	C6-7-8A-9-10	HEK-293	MH500456
66	KE6	C6-7-8A-9tr1-10	HEK-293	MH500457
67	KE6	C6-7-8A-9	HEK-293	MH500460
68	KE6	C6-7-8A-9tr1	HEK-293	MH500459
69	KE6	C6-10	HEK-293	MH500464
70	SE6	C5tr(40)-6-7-8(267)	SH-SY5Y	MH500468
71	SE6	C6-6B-7-8A-9tr1	SH-SY5Y	MK313129
72	SE6	C6-7cr(4)-8A	SH-SY5Y	MH500508
73	HE6	C2A-2B-4-6	HeLa	MK131041
74	HE6	C2A-2B-6	HeLa	MK131044

75	HE6	C4-5-6	HeLa	MH500467
76	HE6	C5tr(27)-6-7(28)	HeLa	MH500465
77	HE6	C6 (2x)	HeLa	MK131047
78	HE6	C6-6Btr(105)	HeLa	MK131048
79	HE6	C6-6B-7-8(162)-10	HeLa	MH500520
80	HE6	C6-6B-7-8A	HeLa	MH500507
81	HE6	C6-7	HeLa	MK131045
82	HE6	C6-7 (2X)	HeLa	MK131043
83	HE6	C6-7-8A	HeLa	MH500461
84	HE6	C6-7-8(162)	HeLa	MK131042
85	HE6	C6tr(109)-7(6)	HeLa	MH500466
86	HE6	C6-8A-9tr1-11	HeLa	MH500505
87	HE6	C6-7-8A-10	HeLa	MH500506
88	GE6	2xC6-7-8A	GM03813	MH500455
89	GE6	C6-8A	GM03813	MH500463
90	KE6B	C6-6B-7-8A-9tr1-10	HEK-293	MH500469
91	KE6B	C6-6B-7-8A-9tr1	HEK-293	MH500470
92	KE6B	C6-6B-7-8A	HEK-293	MH500471
93	KE6B	C6-6B	HEK-293	MH500472
94	KE6B	C6B-7	HEK-293	MH500473
95	KE7	C5-6-7	HEK-293	MH500481
96	KE7	C6-6B-7-8A	HEK-293	MH500475
97	KE7	C6-7-8A-9tr1-10	HEK-293	MH500474
98	KE7	C6-7-8A-10	HEK-293	MH500476
99	KE7	C6-7-8A-9tr1	HEK-293	MH500478
100	KE7	C6-7-8A-9	HEK-293	MH500479
101	KE7	C6-7-8A	HEK-293	MH500480
102	KE7	C6-7cr1-8(130)-10	HEK-293	MH500477
103	KE7	C6tr(35)-7-8(169)	HEK-293	MH500488
104	KE7	C6B-7	HEK-293	MH500483
105	KE7	C7-8A-9tr1	HEK-293	MH500486
106	KE7	C7-8A	HEK-293	MH500482
107	KE7	LiI6B/136-E7-I7/354	HEK-293	MH500489
108	HE7	C6tr(21)-7-8(73)	HeLa	MH500485
109	HE7	C7cp(1)-8(99)	HeLa	MH500484
110	SE7	C6tr(42)-7-8(98)	SH-SY5Y	MH500487
111	GE7	C6-7-8A(2X)	GM03813	MH500524
112	KE8A	C6-7-8A-9tr1	HEK-293	MH500490
113	KE8A	C6-7-8A-9	HEK-293	MH500493

114	KE8A	C6-7-8A	HEK-293	MH500495
115	KE8A	2xC6-7-8A	HEK-293	MH500498
116	KE8A	C6-8A-9	HEK-293	MH500500
117	KE8A	C6tr(109)-8(278)	HEK-293	MH500499
118	KE8A	C7-8A-9tr1	HEK-293	MH500496
119	KE8A	C7tr(10)-8(152)	HEK-293	MH500501
120	KE8A	C7-8(162)	HEK-293	MH500540
121	KE8A	C7-8(163)	HEK-293	MK313130
122	KE8A	C8A	HEK-293	MH500510
123	HE8A	C6-7-8A-10	HeLa	MH500502
124	HE8A	C7-8(167)	HeLa	MH500509
125	HE8A	C8A-9tr1	HeLa	MH500513
126	HE8A	C8(462)	HeLa	MH500539
127	GE8A	C6-7-8A-11	GM03813	MH500511
128	GE8A	C7-8A-11-12	GM03813	MH500512
129	KE10	C6-7-8A-9tr1-10	HEK-293	MH500503
130	KE10	C6-7-8A-10	HEK-293	MH500504
131	mE2A	mC2A-2B-3-4	NSC-34	MH500541
132	mE2A	mC2A-2B(2X)	NSC-34	MH500542
133	mE2A	mC2A-2B-4	NSC-34	MH500543
134	mE2A	mC1tr(17)-2A-2B-3(81)	NSC-34	MH500544
135	mE2A	mC2A-2B	NSC-34	MH500545
136	mE2B	mC2Atr(15)-2B-3(151)	NSC-34	MH500546
137	mE2B	mC2Atr(55)-2B-3(117)	NSC-34	MH500547
138	mE2B	mC2A-2B-3-4-5	NSC-34	MH500548
139	mE2B	mC2A-2B-3-4	NSC-34	MH500549
140	mE2B	mC2Btr(112)-3(81)-4Atr(4-49)(2X)	NSC-34	MH500550
141	mE2B	mC2Bcp1-3-4(96)	NSC-34	MH500551
142	mE2B	mC2Btr(112)-3(81)-4Atr(4-49)	NSC-34	MH500552
143	mE3	mC2A-2B-3-4	NSC-34	MH500553
144	mE3	mC2B-3	NSC-34	MH500554
145	mE4	mC2A-2B-3-4	NSC-34	MH500555
146	mE4	mC4(2X)	NSC-34	MH500556
147	mE4	mC4	NSC-34	MH500557
148	mE5	mCI4(756)-5-6-I6(237)	NSC-34	MH500558
149	mE5	mC5-6-7-8A	NSC-34	MH500559
150	mE5	mC5-6-6B-7	NSC-34	MH500560

151	mE5	mC4-5-7	NSC-34	MH500561
152	mE5	mC5-6-7	NSC-34	MH500562
153	mE5	mC5-6	NSC-34	MH500563
154	mE6	mC2A-2B-3-4-5-6-7-8A	NSC-34	MH500564
155	mE6	mC2A-2B-3-4-5-6	NSC-34	MH500565
156	mE6	mC3-4-5-6-7	NSC-34	MH500566
157	mE6	mC5tr(7)-6-7-8(301)	NSC-34	MH500567
158	mE6	mC6-6B-7	NSC-34	MH500568
159	mE6	C6(2X)	NSC-34	MH500569
160	mE6	mC6-6B	NSC-34	MH500570
161	mE6	mC4-5-6-7	NSC-34	MH500571
162	mE6	mC6	NSC-34	MH500572
163	mE7	mC6tr(81)-7-8(264)	NSC-34	MH500573
164	mE7	mC6tr(36)-7-8(245)	NSC-34	MH500574
165	mE7	mC6tr(30)-7-8(233)	NSC-34	MH500575
166	mE7	mC6tr(30)-7-8(215)	NSC-34	MH500576
167	mE7	mC7-8A	NSC-34	MH500577
168	mE8	mC6tr(88)-7-8(254)	NSC-34	MH500579
169	mE8	mC6tr(30)-7-8(221)	NSC-34	MH500580
170	mE8	mC7tr(41)-8(244)	NSC-34	MH500581
171	mE8	mC7tr(34)-8(213)	NSC-34	MH500582

K, HEK-293; H, HeLa; S, SH-SY5Y; G, GM03813; m, mouse NSC-34; E, exon

Table S4. Target sequences for siRNAs

siRNA Name	Target Sequence
siDHX9-1	GGAUUAAACUGCAAAUAUC
siDHX9-2	GGCUUUGGUUGUUGAAGUA
siDDX5-2	CAACCUACCUUGUCCUUGA
siDDX5-3	GCAUGUCGCUUGAAGUCUA
siDDX17-1	CAAGGAUGGUGGCCGGAGA
siDDX17-2	CGAUAGAGCUGGUUAUGCU

Table S5A. Unusual exon variants identified in human circRNAs.

Type	Generic name	Expanded name	Overall size	Note
1	T1A	C1tr(87)-2A-2B(95)	254	E1tr(87), last 87nt of E1; E2B(95), first 95nt of E2B
1		C1tr1-2A	139	E1tr1, last 67nt of E1, GT motif
1		C1tr1-2A-2B	259	E1tr1, last 67nt of E1, GT motif
1	T1B	C1tr(33)-2A(67)	100	E1tr2, last 33bp of E1, AG motif; E2A(67), first 67nt of E2A
1	T1C	C2Atr(40)-2B-3(156)	316	2Atr(40), last 40nt of E2A; 3(156), first 156nt of E3
1	T1D	C2Atr(40)-2B-4(69)	229	E2Atr(40), last 40nt of E2A;E4(69), first 69nt of E4. A overlapping at BS junction
1	T1E	C2Atr(30)-2B-3-4(39)	390	E2Atr(30), last 40nt of E2A;E4(39), first 39nt of E4.
1	T1F	C2Atr(28)-2B-3(96)	244	E2Atr(28), last 28nt of E2A; E3(96), first 96nt of E3, GT motif.
1	T1G	C2Btr(30)-3-4(18)	249	E2Btr(30) last 30nt of E2B; E4(18) first 18nt of E4;AAAGCCAA is overlapping at BS junction
1	T1H	C2Btr(9)-3-4(36)	246	4(36) first 36nt of E4; 2Btr(9) last 9nt of E2B?
1	T1I	C2Btr(7)-3-4(36)	244	E2Btr(7) last 7nt of E2B; E4(36) first 36nt of E4; T is overlapping at BS junction
1	T1J	C2Btr(7)-3-4(60)	268	E2Btr(7), last 7nt of E2B; E4D first 60nt of E4; A overlapping at BS junction
1		C3tr(117)-4	270	E3tr1 last 117nt of E3, AG motif
1		C3tr(76)-4	229	E3tr2 last 76nt of E3, AG motif
2	T2A	C4tr(146)-5	242	E4tr(146), last 146bp of E4
2		C2A-2B-4-6(61)	406	6A, first 61nt of E6, GT motif
2	T2B	C5tr(27)-6-7(28)	166	E5tr(27) last 27 of E5; E7(28) first 28 of E7
2	T2C	C6tr(109)-7(6)	115	E6tr(109) last 109nt of E6; E7(6) first 6nt of E7; TA overlapping at BS junction
2		CI6B(136)-E7-I7(354)	544	I7, 1-354nt of I7; I6B, 4990-5125(136nt) of I6B; tga overlapping at the joint
3	T3A	C4tr(79)-5-6-8(224)	510	E4tr(79), last 79nt of E4; E8(224), first 224nt of E8, GT motif; T overlapping at BS junction
3	T3B	C4tr(51)-5-6-7-8(320)	632	E4tr(51), last 51nt of E4; E8(320), first 320nt of E8; TT overlapping at BS junction
3	T3C	C4tr(16)-5-6-8(278)	501	E4tr(16), last 16nt of E4; E8(278), first 278nt of E8.
3	T3D	C5tr(40)-6-7-8(267)	472	E5tr(40), last 40nt of E5; E8(267) first 267nt of E8;
3	T3E	C5tr(27)-6-7-8(258)	450	E5tr(27), last 27nt of E5; E8(258) first 258nt of E8
3	T3F	C6tr(109)-8(278)	388	E6tr(109), first 109nt of E6; E8(278), first 278t of E8; AATT overlapping at BS junction
3	T3G	C6tr(42)-7-8(98)	194	E6tr(42), last 42nt of E6; E8(98), first 98nt of E8;
3	T3H	C6tr(35)-7-8(169)	258	E6tr(35), last 35nt of E6; E8(169) first 169nt of E8

3	T3I	C6tr(21)-7-8(73)	148	E6tr(21) last 21nt of E6; E8(73), first 73nt of E8; TAT overlapping at BS junction
3	T3J	C6tr(15)-7-8(194)	263	E6tr(15), last 15nt of E6; E8(194), first 194nt of E8
3	T3K	C6tr(14)-8(188)	202	E6tr(14), last 14nt of E6; E8(188), first 188nt of E8
3		C6-6Btr(105)	170	E6Btr(105), last 105nt of E6B, AG motif
3	T3L	C7tr(10)-8(152)	162	E8(152), first 152nt of E8; E7tr(10), last 10nt of E7
3	T3M	C7cp(1)-8(99)	154	E7cp(1), "g" insertion at 3'ss
3	T3N	C6-7cr1-8(130)-10	416	E8F, first 130nt of E8; E7cr1, first 23nt of I7, Cr1, was used
3		C6-7cr(4)-8A	311	first 4nt of I7 included at 5′ ss , GT motif
3		C6-8Acp(27)	280	E8Acp1, the first 142nt of E8 is included, while 3′ ss 27nt(AG motif) upstream of E8 3′ ss in intron 7
3		C7-8(162)	216	E8(162), first 162nt of E8
3	T3O	C7-8(163)	217	E8(163), first 163 nt of E8, could be E8(162) splicing to E8cp(1)
3		C7-8(167)	221	E8(167), first 167nt of E8, GT motif
3	T3P	C8(462)	462	E8(462), first 462nt of E8

Table S5B. Unusual exon variants identified in mouse circRNAs.

Type	Generic name	Expanded name	Overall size	Note
1	mT1A	mC1tr(17)-2A-2B-3(81)	290	E1tr(17), last 17nt of E1; E3(81), first 81nt of E3
1	mT1B	mC2Atr(55)-2B-3(117)	292	E2Atr(55), last 55nt; E3(117) first 117nt; AT overlapping at BS junction
1	mT1C	mC2Atr(15)-2B-3(151)	286	E2Atr(15) last 15nt; E3(151) first 151nt
1	mT1D	mC2Bcp(2)-3-4(96)	419	E2Bcp(ag), "ag" insertion at 3'ss of E2B; E4(96), first 96nt
1	mT1E	mC2Btr(112)-3(81)-4(4-49)	249	E2Btr(112), last 112nt of E2B; E3(81), first 81nt of E3; E4(4-49), 4-49nt of E4
2		mCI4(756)-5-6-I6(237)	1200	I6, first 1-237nt; I4, last 756 (1963-2718) nt of I4; cuggucu overlapping at junction
3		mC5-6-7-8A	450	E8A, first 192nt of E8
3		mC2A-2B-3-4-5-6-7-8A	990	E8A, first 192nt of E8
3	mT3A	mC5tr(7)-6-7-8(301)	470	E5tr(7) last 7nt of E5; E8(301), first 301nt of E8
3	mT3B	mC6tr(88)-7-8(254)	393	E6tr(88), last 88nt of E6, AG motif; E8(254), first 254nt of E8
3	mT3C	mC6tr(81)-7-8(264)	396	C6tr(81), last 81nt of E6; C8(264), first 264nt of E8; C overlapping at BS junction
3	mT3D	mC6tr(36)-7-8(245)	341	C6tr(36), last 36nt of E6; C8(245), first 245nt of E8; T overlapping at BS junction
3	mT3E	mC6tr(30)-7-8(215)	296	C6tr(30), last 30nt of E6; C8(215), first 215nt of E8; TAC overlapping at BS junction

3	mT3F	mC6tr(30)-7-8(233)	314	C6tr(30), last 30nt of E6; C8(233), first 233nt of E8; ACA overlapping at BS junction
3	mT3G	mC6tr(30)-7-8(221)	302	E6tr(30), last 30nt of E6; E8(221), first 221nt of E8
3		mC7-8A	243	E8A, first 192nt of E8
3	mT3H	mC7tr(41)-8(244)	285	E7tr(41), last 41nt of E7; E8(244), first 244nt of E8
3	mT3I	mC7tr(34)-8(213)	248	E7tr(34), last 34nt of E7; E8(213), first 213nt of E8

Table S6A. Strength score of 5′ splice sites utilized in human circRNAs.

5′ss name	Sequence	MAXENT	MDD	MM	WMM	H bond
I1(NE1-98)	AAGGTGGGTAT	8.23	13.78	7.98	8.95	17.10
I1(NE2-79)	TTGGTAATCAT	3.39	5.68	2.93	3.87	12.00
I1(NE3-33)	CAGGTAAGTTT	10.86	15.08	12.75	13.07	21.30
1	CAGGTGAGGCA	10.07	13.38	10.18	10.76	17.40
2A	AAGGTATGAAA	9.11	12.58	8.40	8.42	14.00
2B	CAGGTTATTTT	3.49	6.98	2.75	4.22	12.10
3	GAGGTAAGGAT	10.28	13.38	10.08	10.94	18.60
4	AAGGTAAACCT	8.14	13.18	6.95	8.03	14.10
5	CCAGTAAGTAA	9.09	13.98	7.50	7.56	15.70
6	ATGGTAAGTAA	11.01	15.48	10.10	10.67	18.70
6(61)	GAAGTATGTTA	6.97	8.68	5.94	5.76	11.10
6B	GGGGTAATGTG	4.85	8.68	4.00	4.75	12.00
7	GGAGTAAGTCT	8.57	12.28	6.36	6.42	14.50
7cr(4)	TAAGTCTGCCA	-7.82	-1.62	-6.51	-0.82	3.00
7cr1	AAAGTGAATCT	0.76	4.18	3.31	4.71	5.70
8A	TGGGTAACTCT	4.34	6.78	4.36	4.92	12.20
8(162)	AAAGTTATGTA	-8.54	0.78	-3.90	-0.25	2.30
8(167)	TATGTAATAAC	-3.72	-2.12	-0.12	1.67	9.40
9	CACGTGAGTAG	9.30	13.38	8.93	8.84	15.90
10	GAGGTGAAAAA	2.91	9.18	5.71	5.69	10.70
11	ACCGTGAGTCA	9.40	12.38	7.69	6.13	12.10
12	GAGGTACGCAC	9.40	12.48	9.46	7.66	14.00

Table S6B. Strength score of 3′ splice sites utilized in human circRNAs.

3′ss name	Sequence	MAXENT	MM	WMM
I1(NE1-98)	ATCACTTCTCTTCTAACAAGGAG	5.73	5.67	6.14
I1E79	TTCATGTTGTTGCGCAATAGATC	5.73	6.15	5.25
I1E33	GAACATTTACCTATGTCTAGCTT	2.84	3.59	3.20
1tr1	TTTGCTATGGCGATGAGCAGCGG	-1.43	-0.63	-1.39
1tr(33)	GTGGCGGCGTCCCGGAGCAGGAG	0.52	-0.32	-0.19
2A	TTATTTCTTACCCTTTCCAGAGC	8.98	9.61	12.88
2B	TTTTATTCCTATTTTCGTAGCAT	6.66	8.62	10.20
3	ATCTTTGATTTCTTTTGTAGTGG	7.29	8.88	10.80
3tr(117)	TTGCTTCAATTGATTTTAAGAGA	4.68	3.43	2.78
3tr(76)	TACACTGGATATGGAAATAGAGA	-1.11	-0.78	-5.27

4	CATATTTTTTCTTTTTAAAGAAT	6.79	7.69	10.48
5	CTTTGAAATATTCCTTATAGCCA	5.44	5.00	4.81
6	ATATCTTTTTCTGTCTCCAGATA	10.90	11.73	13.41
6B	TTTTGTATTTTTTTGTAAAGACA	3.64	5.19	6.96
6Btr(105)	GTATTTTTTTGTAAAGACAGGGT	-0.90	1.75	3.27
7	TTCCTTTATTTTCCTTACAGGGT	10.92	13.08	15.51
8A	TTCTAATTTCTCATTTGCAGGAA	10.77	10.86	10.52
8Acp(27)	TTTAACTGGAATTCGTCAAGCCT	-1.62	-1.30	-2.09
9	TATTTTTGTTTGTTTTTGAGACA	0.41	3.30	3.06
9tr1	TTTGTTTGTTTTTGAGACAGAGT	1.41	3.87	5.97
10	TCAATTCTCTTTATTTGTAGAAT	8.25	9.35	10.53
11	ACCTTCTGCCATGATTTTAGTTT	6.87	6.06	5.43
12	TTTATTTTTGTTTTAAATAGAAA	8.08	8.53	7.69

Note:
For 5′ss, 11-nt sequence at splice site is shown, of which the first 3 nts are considered as exonic sequence and the latter 8 nts as intronic sequence.
For 3′ss, 23-nt sequence at splice site is shown, of which the first 20 nts are considered as intronic sequence and the last 3 nts as exonic sequences.

Abbreviations:
MaxEnt – Maximum Entropy Model
MDD – Maximum Dependence Decomposition Model
MM – First-order Markov Model
WMM – Weight Matrix Model
H bond – H bond score for 5′ss

Table S7A. Strength score of 5′ splice sites utilized in mouse circRNAs.

5′ss name	Sequence	MAXENT	MDD	MM	WMM	H bond
1	CAGGTGAGGTC	10.07	13.38	10.18	10.76	17.40
2A	AAGGTATGAAA	9.11	12.58	8.40	8.42	14.00
2B	CAGGTGATAGA	4.58	9.88	6.51	6.44	12.40
3	GAGGTGAGGCC	8.41	11.78	9.20	9.56	15.70
4	AAGGTAAATGT	8.88	13.38	7.72	9.25	14.90
5	CCAGTAAGTAC	9.09	13.98	7.50	7.56	15.70
6	ATGGTAAGGAC	9.33	13.08	8.41	9.74	15.20
6B	TTGGTAAGCTT	8.72	13.88	7.52	7.77	15.00
7	GAAGTAAGTCT	9.82	13.98	8.09	8.70	14.90
8A	AGTGTGCGTGT	3.14	6.68	4.14	1.94	7.90

Table S7B. Strength score of 3′ splice sites utilized in mouse circRNAs.

3′ss name	Sequence	MaxEnt	MM	WMM
2A	TATTTTTTCTCCCTCTTCAGAGT	10.58	12.06	14.98
2B	TTTATTTACTGTTTTCATAGCAT	5.88	7.40	9.27
3	AACTTTCTTTTCTTTTGTAGTGG	8.15	10.44	13.19
4	GTTTCCTTTTCTTTTTAAAGAAT	7.98	8.51	11.76
5	CTTGGAAATATTCTTTATAGCCA	3.29	3.91	3.45
6	TGGATTTTTTCCATTTGCAGATA	10.56	11.11	10.86
6B	ACACTGACTCGTTTGGCCAGGTT	6.10	5.81	4.00
7	TTTATATGCTCTCTTTACAGGGT	11.13	11.24	11.78
8	GTTTTTTCTTTCAAATTCAGGTT	10.40	10.09	10.24

Note:
For 5′ss, 11-nt sequence at splice site is shown, of which the first 3 nts are considered as exonic sequence and the latter 8 nts as intronic sequence.
For 3′ss, 23-nt sequence at splice site is shown, of which the first 20 nts are considered as intronic sequence and the last 3 nts as exonic sequences.

Abbreviations:
MaxEnt – Maximum Entropy Model
MDD – Maximum Dependence Decomposition Model
MM – First-order Markov Model
WMM – Weight Matrix Model
H bond – H bond score for 5′ss
